# Post-malnutrition growth and its associations with child survival and non-communicable disease risk: a secondary analysis of the Malawi ‘ChroSAM’ cohort

**DOI:** 10.1017/S1368980023000411

**Published:** 2023-08

**Authors:** Natasha Lelijveld, Sioned Cox, Kenneth Anujuo, Abena S Amoah, Charles Opondo, Tim J Cole, Jonathan CK Wells, Debbie Thompson, Kimberley McKenzie, Mubarek Abera, Melkamu Berhane, Marko Kerac, CHANGE study collaborators group

**Affiliations:** 1 Department of Population Health, Faculty of Epidemiology and Population Health, London School of Hygiene and Tropical Medicine, London WC1E 7HT, UK; 2 Emergency Nutrition Network (ENN), Oxford, UK; 3 Centre for Maternal, Adolescent & Reproductive Child Health (MARCH), London School of Hygiene & Tropical Medicine, London, UK; 4 Malawi Epidemiology and Intervention Research Unit, Lilongwe, Malawi; 5 Department of Medical Statistics, Faculty of Epidemiology & Population Health, London School of Hygiene & Tropical Medicine, London, UK; 6 Population Policy and Practice Research and Teaching Department, UCL Great Ormond Street Institute of Child Health, London, UK; 7 Caribbean Institute for Health Research, The University of the West Indies, Kingston, Jamaica; 8 Jimma University, Jimma, Ethiopia

**Keywords:** Rapid weight gain, Severe acute malnutrition, Developmental Origins of Health and Disease, Non-communicable diseases, Catch-up growth, Malawi

## Abstract

**Objective:**

To explore patterns of post-malnutrition growth (PMGr) during and after treatment for severe malnutrition and describe associations with survival and non-communicable disease (NCD) risk 7 years post-treatment.

**Design:**

Six indicators of PMGr were derived based on a variety of timepoints, weight, weight-for-age z-score and height-for-age z-score (HAZ). Three categorisation methods included no categorisation, quintiles and latent class analysis (LCA). Associations with mortality risk and seven NCD indicators were analysed.

**Setting:**

Secondary data from Blantyre, Malawi between 2006 and 2014.

**Participants:**

A cohort of 1024 children treated for severe malnutrition (weight-for-length z-score < 70 % median and/or MUAC (mid-upper arm circumference) < 110 mm and/or bilateral oedema) at ages 5–168 months.

**Results:**

Faster weight gain during treatment (g/d) and after treatment (g/kg/day) was associated with lower risk of death (adjusted OR 0·99, 95 % CI 0·99, 1·00; and adjusted OR 0·91, 95 % CI 0·87, 0·94, respectively). In survivors (mean age 9 years), it was associated with greater hand grip strength (0·02, 95 % CI 0·00, 0·03) and larger HAZ (6·62, 95 % CI 1·31, 11·9), both indicators of better health. However, faster weight gain was also associated with increased waist:hip ratio (0·02, 95 % CI 0·01, 0·03), an indicator of later-life NCD risk. The clearest patterns of association were seen when defining PMGr based on weight gain in g/d during treatment and using the LCA method to describe growth patterns. Weight deficit at admission was a major confounder.

**Conclusions:**

A complex pattern of benefits and risks is associated with faster PMGr. Both initial weight deficit and rate of weight gain have important implications for future health.

Undernutrition in childhood and adult non-communicable diseases (NCD) are important global public health problems. In all its forms, malnutrition accounts for some 45 % of all mortality in children under 5 years^(1)^. Severe malnutrition, particularly wasting, threatens the survival of an estimated 47 million children under five in low- and middle-income countries^(2)^. In parallel, obesity-related NCD are emerging as a leading cause of death among adults in these settings, with nearly three quarters of all NCD deaths occurring in low- and middle-income countries^(3)^. The need to tackle this ‘double burden’ of malnutrition is increasingly urgent^(4)^.

There is convincing evidence that the two major types of malnutrition, undernutrition and overnutrition, are linked via the Developmental Origins of Health and Disease hypothesis. This posits that prenatal undernutrition results in increased NCD risk later in life, and that severe malnutrition during postnatal periods of developmental plasticity is linked to increased risk of NCD^(5–7)^. Mechanisms include the ‘capacity-load’ model whereby the metabolic capacity for homoeostasis is developed in the first 1000 d of life, and any disturbance or limitation during this period results in reduced capacity to deal with metabolic stressors later in life^(8)^. This reduced homoeostatic capacity manifests as increased later-life NCD risk.

Evidence from high-income countries on low birth weight, a marker of prenatal undernutrition, links the rate of catch-up growth in undernourished infants with childhood risk markers for NCD^(9,10)^. For example, infants born small-for-gestational age who were given a nutrient-enriched formula to promote faster weight gain had higher blood pressure 6–8 years later than those given control formula that resulted in slower weight gain^(9)^. In high-income country contexts, where the short-term risk of death from other morbidities is low and environmental stressors such as infectious disease load are relatively few, promoting slower post-malnutrition growth (PMGr) can thus be seen as preferable.

In contrast, in low- and middle-income country contexts with high risks of short-term mortality, the balance of short- and long-term risks associated with PMGr is unknown. Given the strong associations between severe undernutrition (as assessed by low weight and other anthropometric deficits) and high child mortality^(11)^, many treatment programmes prioritise fast recovery to ‘normal’ weight. Children suffering from severe wasting and/or nutritional oedema are currently treated with high-calorie, high-fat ready-to-use therapeutic food (RUTF) or therapeutic milks (f100) that promote rapid weight gain^(12)^. The rationale is that this will reduce the immediate mortality risks associated with being small. However, this priority has been questioned in wider public health literature^(13,14)^. The long-term risk associated with rapid PMGr for survivors of severe malnutrition is currently unknown. More data are needed to find the right balance between a rate of post-malnutrition weight and height gain which optimises survival while minimising long-term NCD risk.

To address this research gap, we must consider what constitutes adequate *v*. too rapid *v*. too slow PMGr and how to define this. There is currently no standard definition. Data are also needed on what effect PMGr may have on later-life NCD risks. Our objectives in this analysis were therefore: (1) to explore rates of PMGr based on a variety of weight- and height-related indicators; (2) to develop different ways of categorising these PMGr indicators into faster and slower growth and (3) to examine associations and patterns between the different indicators and categorisations of PMGr with NCD risk markers.

## Methods

This was a secondary analysis of data collected from children treated for severe wasting and/or nutritional oedema in 2006/7 in Malawi and followed up 7 years later to assess risk of NCD.

The data come from a prospective cohort of severely malnourished children recruited into a randomised controlled trial of pre- and probiotics (PRONUT study)^(15)^. The intervention in this trial showed no overall effect on nutritional recovery nor weight gain. The same children were subsequently followed up at 1-year post-discharge (FuSAM study)^(16)^ and again at 7-year post-discharge (ChroSAM study), where the focus was on longer term growth and on indicators of NCD risk^(6)^.

All children originally admitted for treatment during the recruitment period were eligible for inclusion into this current analysis. Hence, our sample size was constrained by this limit: *a priori* formal sample size calculations for our analyses was not done. Admission criteria at the time of the original PRONUT study were weight-for-length z-score < 70 % median (using the NCHS reference) and/or MUAC (mid-upper arm circumference) < 110 mm and/or bilateral oedema. All patients received initial inpatient care for there was no stand-alone outpatient-based Community Management of Acute Malnutrition (CMAM) programme in the area at that time. Children were initially stabilised with F75 therapeutic milk. As clinical status and appetite improved, they moved to a ‘transition phase’ diet where RUTF was introduced alongside F75 milk. After continued improvement, they moved to a ‘rehabilitation phase’ in which they were transferred home with a 2-week ration of RUTF to continue their recovery as outpatients, returning to the ward for monitoring and to collect another ration every 2 weeks until recovery. Children were discharged from the programme after 2 consecutive visits at or above the target weight of 80 % NCHS median weight-for-height. Minimum time in outpatient care was therefore 4 weeks, with a maximum time of 10 weeks before classification as a ‘non-responder’. Anthropometric data were thus available at: admission to treatment; in-treatment minimum weight; at transfer from hospital to home-based care; at programme discharge and at 1- and 7-year post-discharge.

### Indicators of post-malnutrition growth (exposure variables)

At present, there is no one standard definition of PMGr and we thus explored a range of possible indicators. Six were created, based on the available data and assessment methods commonly used by programmers and policy makers working in this area (Table 1).

**Table 1:** Indicators of PMGr

PMGr indicator number	Abbreviated title	Time period	Definition
1	Δ WAZ/d	From minimum weight during treatment to programme discharge (i.e. maximum weight during treatment)	Change in WAZ per day
2	g/kg per d		Change in grams per kilogram of body weight per day (g/kg per d)
3	g/d		Change in grams per day (g/d)
4	Δ WAZ month	From programme discharge to 1-year post-discharge	Change in WAZ per month
5	g/kg per month		Change in grams per kilogram of body weight per month
6	Δ HAZ month		Average change in HAZ per month

PMGr, post-malnutrition growth; WAZ, weight-for-age z-score; HAZ, height-for-age z-score.

Three indicators focus on the in-treatment period, from the point when children were at their minimum weight during treatment, which was often at admission unless oedema first needed to subside, until discharge from care, which was also their point of maximum weight during treatment. These indicators are based on weight-for-age z-score (WAZ) (using WHO 2006 growth standards), grams per kilogram (g per kg) of body weight and absolute weight in grams. Height-for-age z-score (HAZ) or other measures of height change were not included since there was minimal if any gain in height during the few weeks of in-programme treatment. Since the length of stay until discharge criteria were met varied by individual, gains in weight indicators were calculated ‘per day’ to capture rates of gains.

Three other PMGr indicators were based on the post-treatment period, between discharge and 1-year post-discharge. We expected a more ‘natural’ level of catch-up growth during this time since high-energy additional foods were no longer being provided. Again, this included WAZ and g per kg, and an additional metric: HAZ (WHO 2006). Growth increment was calculated per month to account for any differences in the follow-up period.

Each indicator of PMGr was subsequently categorised using each of three different methods:

1. No categorisation (continuous data)
2. Quintiles (Q1 slowest and Q5 fastest)
3. Latent class analysis (LCA) machine learning (six classes).

### Survival (outcome variable)

Survival was defined as the child being seen or reliably reported to be alive at 7 years after the original discharge. Deaths were defined as those who were reliably reported to have died by a family member anytime between admission and 7-year post-discharge. Most of the children who died did so either during treatment or within the 1st year post-discharge.

### Non-communicable disease risk indicators (outcome variables)

Seven NCD indicators were selected as outcomes of interest and assessed 7-year post-discharge from treatment. These were blood pressure (systolic and diastolic), handgrip strength, waist circumference, waist/hip ratio, lean mass index (LMI), fat mass index and HAZ. These indicators are all commonly associated with NCD risk in adulthood and were previously analysed as part of the ChroSAM study^(17–21)^. Blood pressure was assessed using an Omron digital device with a paediatric cuff. Muscle strength was measured with a Takei Grip-D device (Takei, Niigata, Japan). LMI and fat mass index were assessed by bioelectrical impedance analysis using a Quadscan 4000 device (Bodystat). Usually, the bioelectrical impedance analysis output impedance (Z) is adjusted for height (HT) to give the impedance index (HT^2^/Z), which is then used to predict total body water or lean mass via population-specific empirical equations. Lean mass can then be divided by height-squared, to give LMI in the same kg/m^2^ units as BMI. Obtained via impedance, however, LMI is essentially HT^2^/Z/HT^2^, which simplifies to 1/Z. In the absence of a population-specific equation, 1/Z can therefore be used as a valid marker of LMI, as it differs only by two constants^(22)^. A proxy for fat mass index can then be calculated as the regression residual of BMI on LMI, on the assumption that greater BMI for a given LMI value indicates increased adiposity^(22)^. Lastly, while HAZ change during early post-treatment period was an exposure variable in these analyses, its assessment at 7-year post-discharge was also an outcome indicator of NCD risk^(23)^.

### Statistical analyses

Data were analysed using Stata SE 17 (StataCorp LLC). Implausible z-scores exceeding +/– 10 were set as missing. This is more generous than the WHO recommendation for cleaning survey data since these are treatment data where children are known to have very extreme deficiencies. For the ‘no-categorisation’ method, each PMGr indicator was assessed for its association with death using data from the full prospective cohort, using logistic regression. We used the mean change as well as the minimum and maximum change in each of the six PMGr indicators to describe the growth patterns of survivors. We also explored the association of each PMGr indicator with original admission WAZ using linear regression analysis as it is important to know which indicators may be affected by possible confounding of greater weight deficits at admission. Associations between PMGr indicators and NCD outcomes were analysed using only data from survivors, using linear regression. Age, sex and HIV status were included as *a priori* confounding factors in adjusted regression analyses, based on the literature and previous analyses^(6)^. HAZ at follow-up was also included as a potential confounder when examining blood pressure as the outcome, as a known confounder (associated with both rate of growth and blood pressure) in the literature. Adjusting for end-point BMI was explored but not included since it was considered to be on the causal pathway between rapid weight gain and NCD risk factors.

For the quintiles method, individuals were assigned to quintiles based on each of the PMGr indicators, quintile 1 being the smallest values and quintile 5 the highest. Associations between PMGr indicators and NCD outcomes were explored visually using boxplots and using linear regression where the NCD risk factor was a continuous outcome and the quintile number (1–5) was the predictor.

For the LCA method, latent classes were identified using a generalised structural equation model with Gaussian-distributed WAZ, weight and HAZ measurements across five timepoints (admission, minimum weight during treatment, discharge from inpatient care, discharge from care and 1-year post-discharge). Since LCA uses anthropometric values at multiple fixed timepoints to identify patterns, it does not account for different lengths of treatment. A balance of sample size and model fit according to Bayesian information criteria were used to determine the number of classes to extract; models with lower Bayesian information criteria and classes with at least eight individuals each were preferred. We also conducted additional LCA with WAZ, weight and HAZ at each timepoint standardised by admission WAZ/weight/HAZ, respectively, to adjust for the confounding influence of admission anthropometry.

The LCA-predicted PMGr classes were plotted against the relevant anthropometric measures to facilitate comparison. As with the quintile-based method, boxplots for each of the LCA-derived classes and NCD indictors were created to explore associations, and simple and multivariable linear regression analyses were conducted.

## Results

**Fig. 1:**
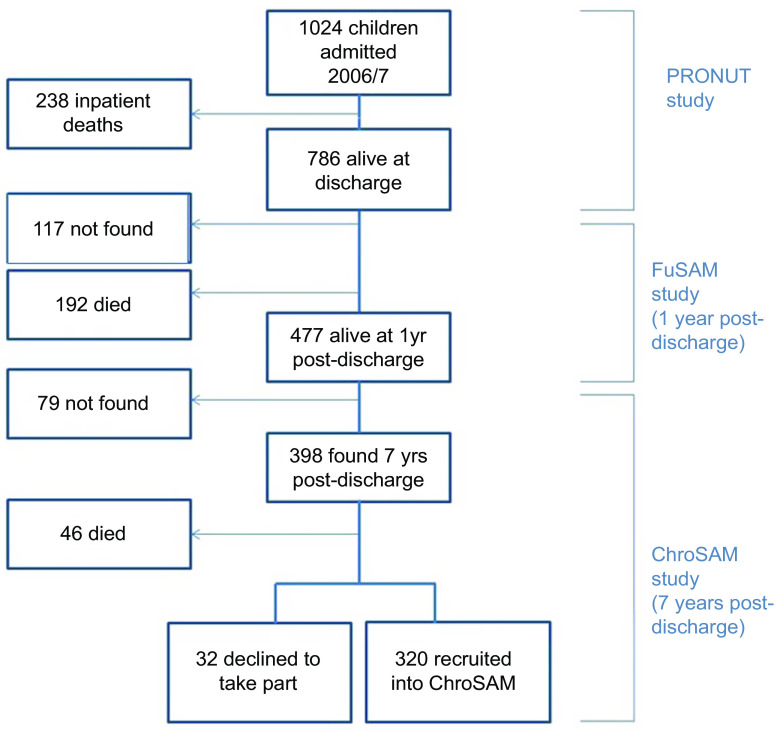
Participant flow diagram

Anthropometric data at the point of discharge from treatment were available for 786 children. Follow-up data were available for 477 children up until 1-year post-discharge. Data on NCD risk factors at 7-year post-discharge were available for 320 children. These data, which were used for defining PMGr, are described in Fig. 1.

### Descriptive analyses of survivors

**Fig. 2:**
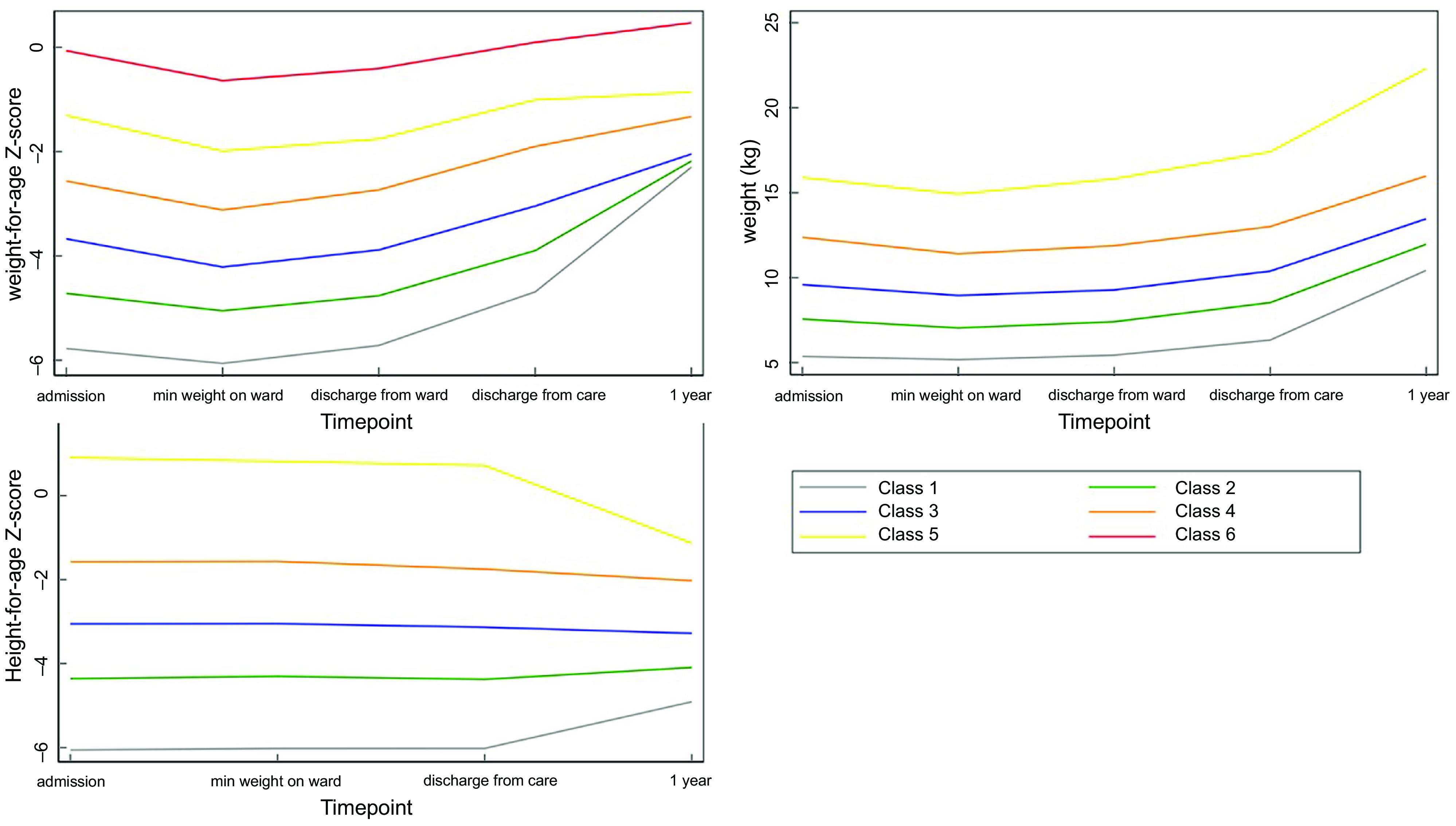
Growth patterns for weight-for-age z-score, weight and height-for-age z-score described based on LCA classes. *Class 1 = lowest initial admission weight and the steepest change. Classes 5 and 6 = least/shallowest change

A total of 320 survivors seen at 7-year post-discharge were included in this analysis (Fig. 1). At admission, the sample had a high prevalence of HIV (approximately 30 %) and 83 % had oedematous malnutrition (Table 2). Three-quarters were stunted (HAZ < -2) at admission (78 %) and a large proportion (41 %) remained stunted 7-year post-discharge. Baseline demographics of those lost-to-follow-up are presented in Annex Table 1. Across all six indicators of PMGr, children had wide ranging changes in growth status, including both positive and negative (Table 3, Annex Figure 1). The method of defining PMGr influenced the pattern of WAZ change over the course of the cohort timepoints (Annex Figure 2). Both the quintile categories and the LCA-defined categories (described in Fig. 2) highlight the link between greater anthropometric deficits at admission to treatment and rapid PMGr; this is especially true for post-discharge growth (Table 4). Those with larger WAZ at admission have slower PMGr after treatment (Table 4). The sample size for each of the LCA classes is presented in Annex Table 2.

**Table 2:** Descriptive demographics of survivors at different timepoints (*n* 320)

Variables	Admission			Discharge			1-year post-discharge			7-year post-discharge		
	%	Median	IQR	%	Median	IQR	%	Median	IQR	%	Median	IQR
Age (months)		24·0	16–34		25·7	18–35		41·5	33–50		111·4	104–124
% Males	54·6											
% HIV positive	29·5											
Anthropometry		Mean	sd		Mean	sd		Mean	sd		Mean	sd
% Oedema	83·1			0·0			0·32			0·0		
Weight (kg)		8·68	2·74		9·58	2·76		13·04	3·16		23·99	4·91
Height (cm)		78·23	10·2		78·71	9·88		88·38	11·3		124·99	9·03
MUAC (cm)		12·33	1·89		13·52	1·76		15·22	1·49		17·24	1·96
Anthropometric Indicators												
% Stunted (HAZ < –2)	78·2			81·7			81·9			41·1		
% Wasted by MUAC (< 12·5 cm)	51·9			26·0			2·9			0		
% Wasted by WHZ (< –2)	49·8			19·7			3·0			0		
% Underweight (WAZ < –2)	75·4			62·1			40·0			33·0		
		Median	IQR		Median	IQR		Median	IQR		Median	IQR
Days since admission		0			35	30–51		531	493–558		2682	2611–2755

HAZ, height-for-age z-score; WHZ, weight-for-height z-score WAZ, weight-for-age z-score; MUAC, mid-upper arm circumference.

Z-scores based on WHO 2006 and WHO 2007 references.

**Table 3:** Logistic regression of mortality on growth rate (PMGr as continuous predictor) (*n* 320)

Timing of PMGr	PMGr indicator	Survivors *n* 395		Deaths *n* 328		Death unadjusted OR		*P*	Death adjusted OR		*P*
		Mean	sd	Mean	sd	OR	95 % CI		Adjusted OR	95 % CI	
During treatment period	Δ WAZ per day	0·04	0·03	0·02	0·07	0·001	0·00002, 0·08	0·002	0·19	0·01, 6·34	0·35
	g/kg per d	5·50	4·21	4·11	11·7	0·98	0·96, 0·99	0·03	0·99	0·97, 1·01	0·55
	g/d	42·2	33·9	25·7	72·5	0·99	0·98, 0·99	< 0·001	0·99	0·99, 1·00	0·04
Between discharge and 1-year post-discharge	Δ WAZ per month	0·06	0·09	0·04	0·15	0·03	0·00, 12·1	0·25	0·01	0·00, 5·87	0·15
	g/kg per month	27·2	20·3	12·7	12·3	0·92	0·88, 0·96	< 0·001	0·91	0·87, 0·94	< 0·001
	Δ HAZ month	0·003	0·08	−0·012	0·12	0·07	0·0001, 44·2	0·42	0·01	9·7e–6, 20·2	0·25

PMGr, post-malnutrition growth; WAZ, weight-for-age z-score; HAZ, height-for-age z-score.

*Adjusted for age, sex and HIV status; HIV and, for PMGr1, younger age are independently associated with death.

**Table 4:** Mean, minimum and maximum change for each of the PMGr indicators, and their association with admission WAZ (*n* 320 survivors)

PMGr indicator	Mean change	sd	Minimum	Maximum	Change in growth rate per unit increase in admission WAZ*	95 % CI
1 Δ WAZ per day	0·03	0·03	−0·04	0·21	−0·001	0·93, –0·002
2 g/kg per d	5·31	3·66	−3·66	27·39	**−0·36**	**–0·63, –0·09**
3 g/d	40·6	27·1	−38·6	155·9	0·83	–1·05, 2·72
4 Δ WAZ per month	0·06	0·08	−0·14	0·39	**−0·03**	**–0·03, –0·02**
5 g/kg per month	26·3	18·1	−3·8	117·0	**−5·17**	**–6·48, –3·87**
6 Δ HAZ per month	−0·00	0·08	−0·24	0·29	**−0·02**	**–0·02, –0·01**

Change in PMGr1, 2 and 3 is from minimum weight during treatment to programme discharge; for PMGr4, 5 and 6 it is from programme discharge to 1-year post-discharge.

Note that admission WAZ is the exposure and change in PMGr is the outcome. WAZ, weight for age z-score; PMGr, post-malnutrition growth; HAZ, height-for-age z-score, as per the specific definitions. Bold = *P* < 0·05.

* Results of linear regression analysis, presented as coefficient (95 % CI).

### Survival as an outcome

Growth data were available for 395 survivors and 328 children who died. Using the ‘no categorisation’ method, faster growth during treatment (change in WAZ/d, g/kg per d and g/d) was associated with significantly reduced odds of death (crude OR 0·001, 0·98 and 0·99, respectively) (Table 3). However, after adjustment for age and HIV, only greater g/d was significantly associated with reduced odds of death (adjusted OR 0·99, 95 % CI 0·99, 1·00). Younger age and HIV were positively associated with increased odds of death, and HIV was also associated with slower weight gain during treatment. Between treatment and 1-year post-discharge, only faster growth in g/kg per month was associated with reduced odds of death, both before and after adjustment (adjusted OR 0·91, 95 % CI 0·87, 0·94, *P* < 0·001).

### Non-communicable disease outcomes

#### No categorisation method

There were mixed patterns of association between rapid PMGr and NCD outcomes in this young cohort; only a few indicators of PMGr were significantly associated with NCD indicators after adjusting for age, sex and HIV status (Table 5). In the unadjusted analysis, the general trend was that faster weight gain was associated with reduced blood pressure, increased waist:hip ratio and increased HAZ, with mixed associations across the other NCD outcomes. In the adjusted analysis, faster WAZ and g/kg per d gains during treatment were associated with a larger waist circumference (22·8, 95 % CI 5·73, 39·9, *P* = 0·009 (WAZ) and 0·12, 95 % CI 0·01, 0·24, *P* = 0·04 (g/kg per d)) and faster gains in g/d during treatment were associated with larger waist:hip ratio (0·02, 95 % CI 0·01, 0·03, *P* = 0·003). Faster gains in WAZ and g/d during treatment were associated with significantly larger HAZ at 7-year follow-up (6·62, 95 % CI 1·31, 11·9, *P* = 0·02 (WAZ) and 0·01, 95 % CI 0·001, 0·01, *P* = 0·003 (g/d)). Post-discharge, faster gains in HAZ were associated with a smaller waist circumference (-6·15, 95 % CI -12·1, -0·22, *P* = 0·04). See full panel of scatter plots for all NCD outcomes in Annex Figure 3a–h.

**Table 5:** Association between PMGr indicators and NCD risk outcomes in survivors at 7-year post-discharge (*n* 320) (linear regression)

NCD indicator	PMGr indicator	Unadjusted difference	*P*	95 % CI	Adjusted difference	*P*	95 % CI
Systolic blood pressure (mm Hg)	1	9·24	0·69	–33·2, 51·7	−14·7	0·50	–57·5, 28·1
	2	0·00	0·98	–0·29, 0·30	−0·04	0·79	–0·32, 0·24
	3	0·03	0·07	–0·00, 0·06	−0·01	0·76	–0·04, 0·03
	4	−19·3	0·008	–33·5, –5·04	−12·3	0·12	–27·9, 3·31
	5	−0·09	0·01	–0·15, –0·02	−0·04	0·28	–0·11, 0·03
	6	−5·95	0·46	–22·0, 10·1	−9·49	0·24	–25·4, 6·39
Diastolic blood pressure (mm Hg)	1	−30·93	0·12	–70·85, 9·00	−33·3	0·12	–75·5, 8·87
	2	−0·19	0·17	–0·46, 0·08	−0·18	0·20	–0·45, 0·09
	3	−0·01	0·51	–0·04, 0·02	−0·17	0·29	–0·05, 0·01
	4	−14·1	0·04	–27·8, –0·38	−13·4	0·09	–29·2, 2·34
	5	−0·05	0·11	–0·11, 0·01	−0·04	0·23	–0·11, 0·03
	6	2·94	0·71	–12·5, 18·4	−2·44	0·77	–18·5, 13·7
Hand grip (kg)	1	6·25	0·46	–10·3, 22·8	5·16	0·52	–10·4, 20·7
	2	−0·04	0·55	–0·16, 0·09	−0·00	0·98	–0·11, 0·11
	3	0·02	0·007	0·00, 0·03	0·01	0·26	–0·01, 0·02
	4	−3·62	0·19	–9·05, 1·81	1·34	0·65	–4·33, 7·01
	5	−0·04	0·008	–0·07, 0·00	0·00	0·82	–0·02, 0·03
	6	4·57	0·13	–1·37, 10·5	4·55	0·12	–1·25, 10·4
Waist circumference (cm)	1	21·3	0·02	3·78, 38·8	22·8	0·009	5·73, 39·9
	2	0·07	0·32	–0·06, 0·19	0·12	0·04	0·01, 0·24
	3	0·00	0·79	–0·00, 0·00	0·00	0·69	–0·00, 0·00
	4	−6·62	0·01	–11·9, –1·32	−4·60	0·17	–9·89, 1·76
	5	−0·04	0·004	–0·07, –0·01	−0·00	0·90	–0·03, 0·03
	6	−4·47	0·14	–10·5, 1·54	−6·15	0·04	–12·1, –0·22
Waist:hip ratio	1	0·09	0·54	–0·19, 0·37	0·11	0·46	–0·18, 0·39
	2	0·00	0·17	–0·00, 0·003	0·00	0·19	–0·00, 0·003
	3	0·03	< 0·001	0·02, 0·04	0·02	0·003	0·01, 0·03
	4	0·09	0·07	–0·01, 0·19	0·01	0·92	–0·10, 0·11
	5	0·01	0·02	0·00, 0·01	0·00	0·49	–0·00, 0·001
	6	0·02	0·76	–0·13, 0·09	−0·04	0·51	–0·15, 0·06
Lean mass index (BIA)	1	2·66	0·50	–5·15, 19·5	0·88	0·83	–7·22, 8·97
	2	−0·10	0·71	–0·06, 0·04	−0·01	0·64	–0·07, 0·04
	3	0·00	0·56	–0·00, 0·01	−0·00	0·66	–0·01, 0·00
	4	−2·36	0·08	–5·03, 0·32	−1·58	0·31	–4·66, 1·49
	5	−0·01	0·12	–0·02, 0·003	0·00	0·95	–0·01, 0·01
	6	−1·18	0·43	–4·15, 1·79	−0·69	0·66	–3·82, 2·43
Fat mass index (BIA)	1	0·53	0·84	–4·76, 5·82	2·02	0·47	–3·46, 7·49
	2	−0·00	0·89	–0·04, 0·03	0·01	0·54	–0·03, 0·05
	3	0·01	0·04	0·00, 0·01	0·00	0·09	–0·00, 0·01
	4	0·08	0·93	–1·69, 1,85	−0·03	0·97	–2·06, 1·99
	5	−0·01	0·21	–0·13, 0·003	−0·00	0·65	–0·01, 0·01
	6	0·08	0·94	–1·89, 2·04	−0·94	0·37	–2·99, 1·11
HAZ	1	8·11	0·002	2·89, 13·3	6·62	0·02	1·31, 119
	2	0·04	0·03	0·004, 0·08	0·03	0·13	–0·01, 0·06
	3	0·001	0·64	–0·00, 0·01	0·01	0·003	0·00, 0·01
	4	−0·32	0·72	–2·08, 1·44	−0·12	0·90	–2·04, 1·81
	5	0·01	0·06	–0·00, 0·02	0·00	0·34	–0·01, 0·01
	6	−0·64	0·51	–2·54, 1·26	0·32	0·75	–1·63, 2·28

PMGr, post-malnutrition growth; NCD, non-communicable disease; BIA, bioelectrical impedance analysis; HAZ, height-for-age z-score.

Linear regression, both simple and multivariable, adjusting for age, sex and HIV status. Blood pressure outcomes are also adjusted for height at the time of blood pressure measurement. Predictor variable is continuous values for PMGr indicators.

#### Quintiles method

**Fig. 3:**
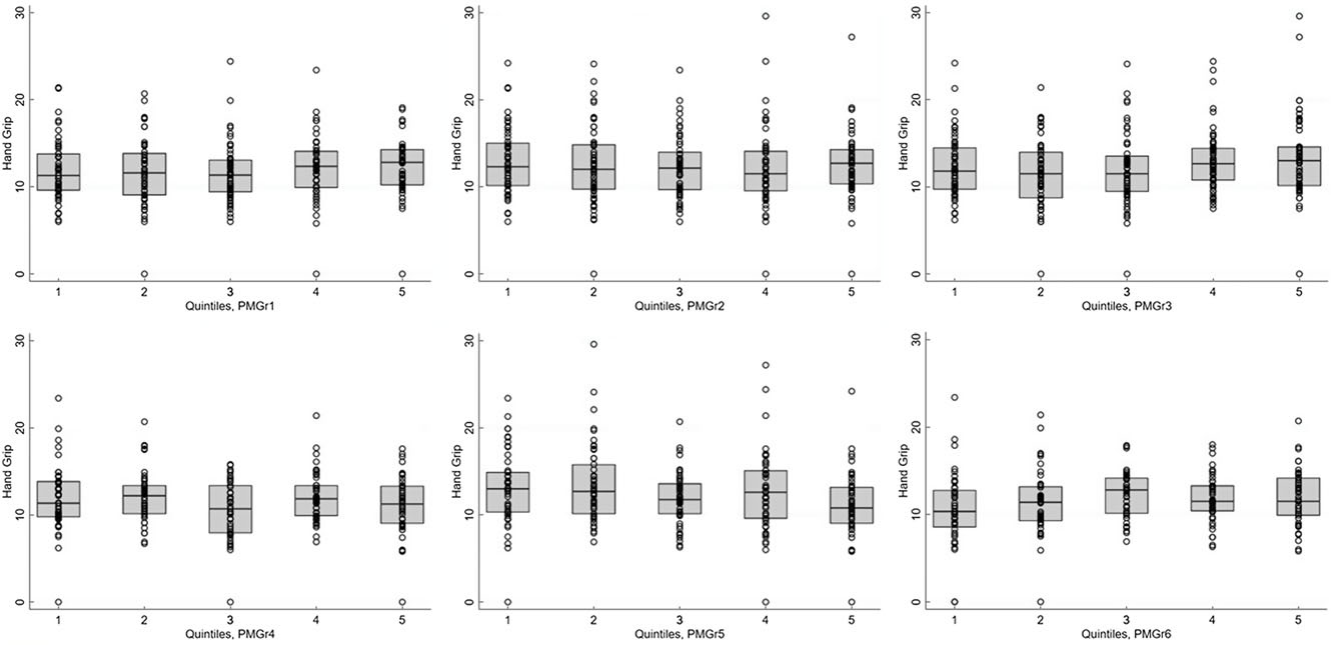
Boxplots for the association between each quintile of post-malnutrition growth (PMGr) and handgrip strength, for the 6 PMGr indicators. Note, quintile 1 is the slowest and quintile 5 is the fastest growth. PMGr1, 2 and 3 = during treatment, PMGr4, 5 and 6 = from discharge to 1-year post-discharge. PMGr1 = Δ WAZ/d, PMGr2 = g/kg per d, PMGr3 = g/d, PMGr4 = Δ WAZ month, PMGr5 = g/kg per month, PMGr6 = Δ HAZ month

**Fig. 4:**
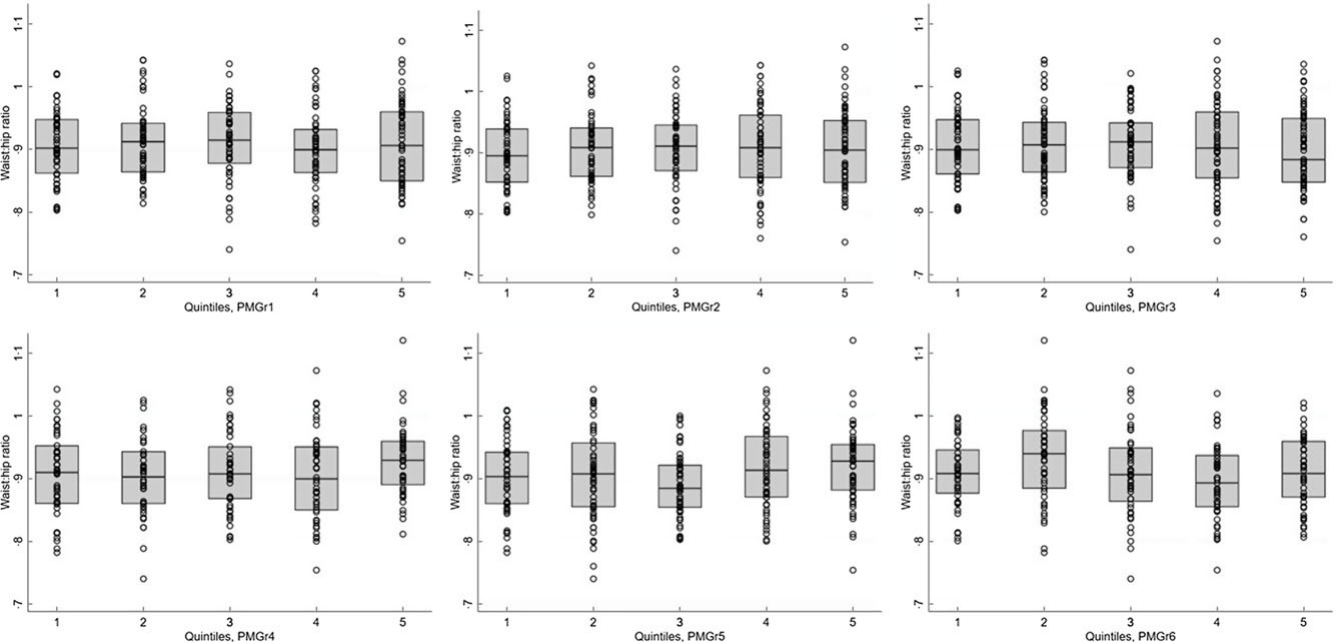
Boxplots for the association between each quintile of post-malnutrition growth (PMGr) and waist circumference, for the 6 PMGr indicators. Note quintile 1 is the slowest and quintile 5 is the fastest growth. PMGr1, 2 and 3 = during treatment, PMGr4, 5 and 6 = from discharge to 1 year post-discharge. PMGr1 = Δ WAZ/d, PMGr2 = g/kg per d, PMGr3 = g/d, PMGr4 = Δ WAZ month, PMGr5 = g/kg per month, PMGr6 = Δ HAZ month

**Fig. 5:**
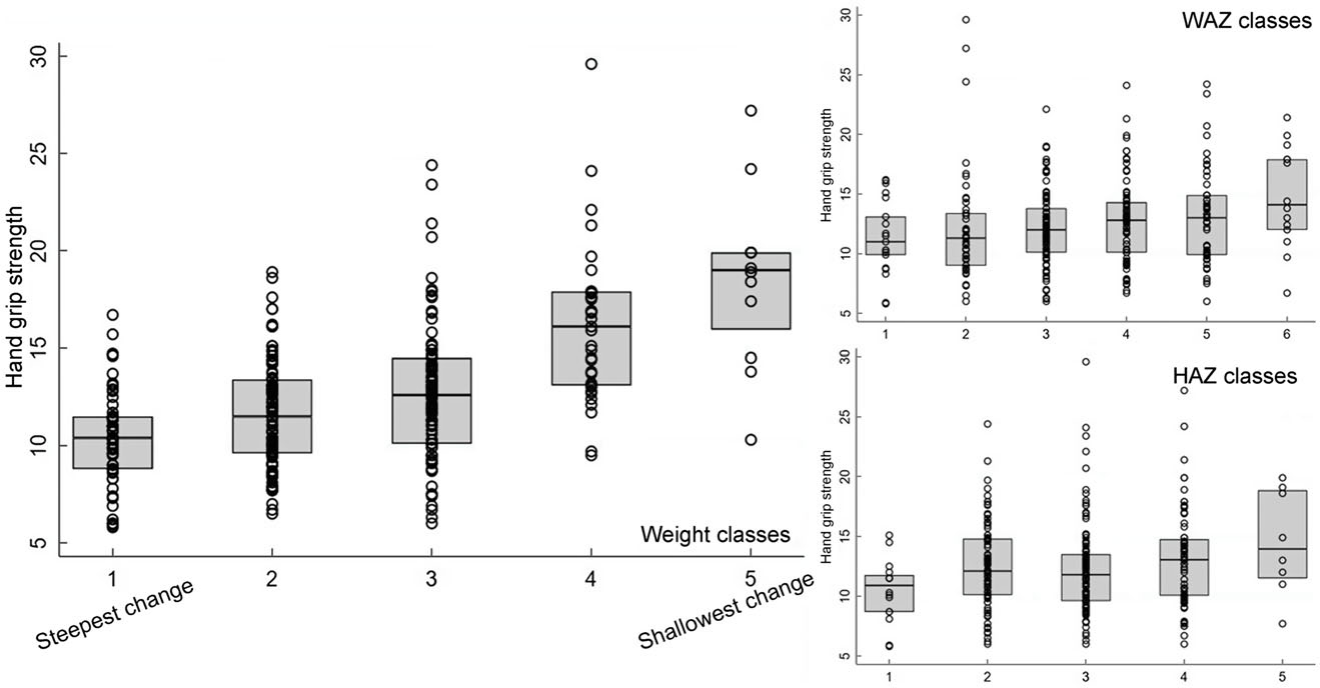
Boxplots for the association between latent class analysis classes and waist:hip ratio, for weight, weight-for-age z-score and height-for-age z-score. Note, class 1 has the lowest initial admission weight and the steepest change; class 5 has the lowest change in PMGr indicators

When the rate of PMGr was split into quintiles, there were few instances of association with NCD indicators, especially after adjustment for age, sex and HIV status (Annex Table 3; Figs 3 and 4). In general, faster PMGr after discharge was associated with lower blood pressure (PMGr4 crude difference –1·08, 95 % CI –1·92, –0·24, *P* = 0·01; PMGr5 crude difference -0·92, 95 % CI –1·75, –0·09, *P* = 0·03) and smaller waist circumference (PMGr4 crude difference –0·38, 95 % CI –0·69, –0·06, *P* = 0·02; PMGr5 crude difference –0·47, 95 % CI –0·82, –0·12, *P* = 0·01). After adjustment, only waist circumference was associated with PMGr defined by change in WAZ per day during treatment (adjusted difference 0·32, 95 % CI 0·01, 0·63, *P*-value 0·04) and change in g/d during treatment (adjusted difference 0·37, 95 % CI 0·06, 0·68, *P*-value 0·02) (Fig. 5). This was similar to findings from the method with no categorisation of PMGr. More boxplots are presented in Annex Figures 4 a–h.

#### Latent class analysis method

**Fig. 6:**
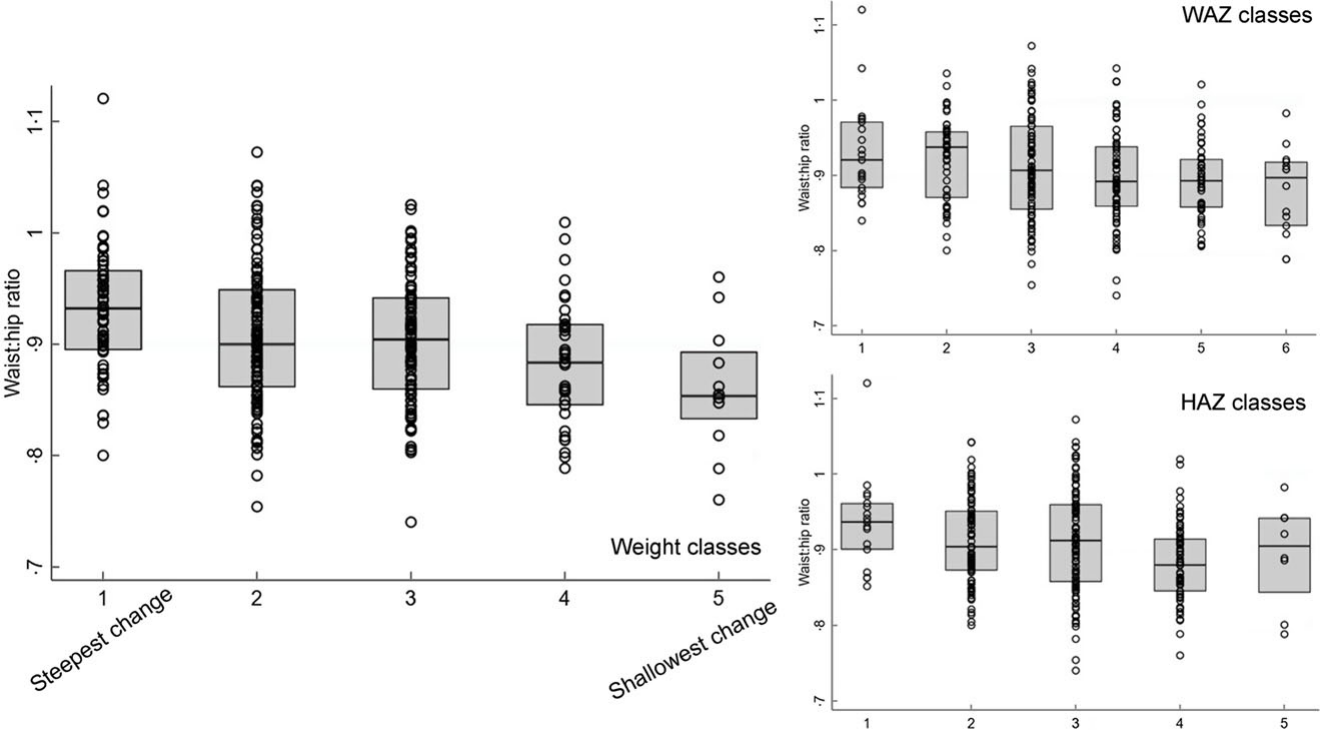
Boxplots for the association between latent class analysis classes and hand grip strength, for weight, weight-for-age z-score and height-for-age z-score. Note, class 1 has the lowest initial admission weight and the steepest change; class 5 has the lowest change in post-malnutrition growth indicators

The LCA method showed clear associations between growth patterns and NCD risk factors. Results from regression analysis of NCD indicators according to the latent classes are summarised in Annex Table 4. Latent class 1 had the lowest initial admission weight and the steepest change; class 5 had the lowest change in PMGr indicators. Faster PMGr was associated with lower systolic blood pressure (weight crude difference 2·37, 95 % CI 1·38, 3·36, *P*-value < 0·001; HAZ crude difference 1·18, 95 % CI 0·01, 2·36, *P* = 0·04), weaker hand grip strength (adjusted difference for WAZ: 0·58, weight: 0·99, HAZ: 0·96, all *P* < 0·001) (Fig. 6), smaller waist circumference (adjusted difference WAZ: 0·95, weight 1·16, HAZ: 1·22, all *P* < 0·001), larger waist-hip ratio (adjusted difference WAZ: -0·01, *P* < 0·001, weight: -0·01, *P* = 0·004, HAZ: -0·02, *P* < 0·001) (Fig. 5), more lean mass (adjusted difference WAZ: 0·29, *P* < 0·001, weight: 0·42, *P* = 0·002) and more fat mass (adjusted difference WAZ 0·17, *P* = 0·002, weight: 0·19, *P* = 0·03). No pattern of association with diastolic blood pressure was discernible (all boxplots are in Annex Figures 5 a–h). The LCA classes highlight the large influence of admission anthropometry on the subsequent growth pattern and on NCD risk factors (association between admission WAZ, weight (kg) and HAZ, and the NCD risk factors is presented in Annex Table 5). To try to adjust for the influence of initial admission WAZ, weight and HAZ on subsequent growth, we conducted an additional LCA after standardising initial admission WAZ/weight/HAZ, respectively, and found similar albeit slightly attenuated magnitudes of association (see Annex Figure 6 and 7 a–g).

## Discussion

This exploratory analysis of methods for describing PMGr and its impact on later NCD risk factors is the first of its kind for survivors of severe malnutrition. We found that faster weight gain was associated with better odds of survival. Faster/steeper weight gain was also associated with stronger hand grip strength and larger HAZ 7-year post-discharge, both indicators of better health. However, there was also an association between faster PMGr and increased waist:hip ratio in later childhood, which can be an indicator of risk for later-life heart disease and diabetes^(24)^.

We found that the definition of PMGr had a large impact on how children are categorised and their associations with later health indicators. The clearest patterns were seen with PMGr indicator 3 which was based on the measure of g/d during treatment. Also, the analysis method (i.e. the means of categorising growth) had clear impact, and the LCA method of categorising growth patterns showed the clearest effect. While the smooth regression (no categorisation) method and the quintiles method described the children’s growth velocity, the LCA method was more focused on the pattern of growth. The pattern seemed to have more of an influence on later health and was highly influenced by the severity of anthropometric deficits at admission. It is difficult to separate the risk of faster weight gain and greater weight deficits at admission to treatment, since the two are closely related^(25)^. Those who start the smallest have the furthest to catch-up, plus there is also regression to the mean. Oedematous malnutrition is also a possible important confounder in our cohort since oedematous admission is less severely wasted at admission and tends to have lower weight gain. It is also less frequently associated with low birth weight and fewer long-term consequences^(26)^. In an observational study such as ours, it is not possible to disentangle the independent impact of these factors.

There may be some difference in NCD associations based on the timing of PMGr. Faster in-treatment growth (indicators 1–3) is associated with larger waist circumference, whereas faster post-treatment growth (indicators 4–6) is associated with smaller waist circumference. The LCA patterns of growth show that the greatest changes in weight and height happen during the post-treatment period. It is important to note that larger waist:hip ratio in these survivors, and in children generally, may not have the same meaning as when applied to adults in high-income countries^(27)^. Most of the evidence that associates larger waist:hip ratio with NCD relates to the greater deposition of fat on the core body, close to vital organs (i.e. waist) rather than on the peripheral body (i.e. hips)^(28,29)^. Previous analyses of these data found that survivors of severe malnutrition tended to have smaller waists than controls, but even smaller hips. This results in larger waist:hip ratio, but rather than having extra fat on their waists they appear to lack gluteofemoral fat^(19)^. This may still have adverse health effects, as both peripheral fat and peripheral lean mass (i.e. larger hip circumference) are thought to be protective against NCD, both of which are lacking in SAM survivors generally and especially in those with the steepest PMGr patterns^(30)^. Fat distribution and proportion of visceral fat were also one of the key outcomes associated with rapid PMGr in a related analysis of adult SAM survivors in Jamaica^(31)^.

Other studies that have described PMGr and its associations with NCD risk factors have largely focused on prenatal or very early postnatal nutrition. Our indicators of PMGr were influenced by this literature as well as indicators currently used in severe malnutrition programming. For example, LCA of WAZ has been used to identify growth profiles in low birth weight and small-for-gestational age infants^(32,33)^. We include a measure of linear growth since ‘catch-up growth’ is commonly defined in small-for-gestational age infants as height velocity^(34)^. Other commonly used definitions of rapid weight gain or rapid catch-up growth in the literature include a change of WAZ > 0·67 or crossing of one centile line on a growth chart, both of which have been associated with later-life obesity when it occurs during infancy^(35)^. However, neither of these could be used in relation to those treated for severe malnutrition since all children exceed these definitions.

Another indicator we included was based on weight gain in g/kg per d, as this is often used in severe malnutrition programming. Standards for humanitarian programming recommend that ‘good’ weight gain should be at least 10 g/kg per d^(36)^. This is difficult to achieve for many programmes – the average in this dataset was 5·31g/kg per d. Such targets are not based on any hard evidence regarding healthiest rate of weight gain^(37)^. Since the advent of outpatient-focused CMAM treatment programmes, weight gain per day has been overall slower than in old-style inpatient-only programmes^(38)^. This is observed in spite of CMAM programme using high energy (high-fat, high sugar) RUTF. Reasons for this include longer in-programme stays. Also despite the use of high energy RUTF, there is currently no evidence that the type of weight gain (i.e. fat *v*. lean) associated with CMAM treatment programmes contains an excessive amount of fat tissue^(39,40)^. Our results suggest that both severity at admission and rate of weight gain need to be considered in future studies. Rate of in-programme gain can be easily controlled by food rations given at different energy doses (WHO recommends a range, although most programmes give doses at the upper end of this range^(41)^). Admission weight deficit is less easy to control – though this finding does reiterate the need for prevention and proactive case finding to catch children early in their deterioration. Programmes for moderate malnutrition are important in this prevention role, limiting and monitoring further weight loss^(42)^.

Experimental studies on diet in early infancy, such as breastfed *v*. formula-fed infants, or use of formula milks with a range of calorie or protein contents, suggest a causal link between early growth acceleration and childhood risk markers for NCD^(43)^. However, as in these results, they have also found some benefits of rapid infant weight gain, such as better neurodevelopment, and the risk-benefit balance is tipped in favour of the promotion of rapid growth in infants born preterm^(44)^. This level of experimental evidence to deduce where the balance of risks and benefits lie needs to be urgently replicated for children being treated for severe malnutrition. Recent studies exploring simplified protocols with reduced dosages of RUTF have observed slower weight gain but without any obvious ‘penalty’ in terms of increased mortality or reduced recovery rate from malnutrition^(45,46)^. In most settings globally, child mortality is falling and it may be that even in this most vulnerable group of severely malnourished children, the optimal risk/benefit balance of rapid PMGr is different today compared to even 10 years ago.

### Limitations

Among the key limitations of our secondary analysis is the impact of survivor bias. More than half of the original cohort are known to have died either during or since treatment. These were generally children who had slow initial in-programme growth. Had they lived they may have changed our results in either direction: they may have had more or less NCD risk than the ‘healthier’ survivors. Children lost-to-follow-up (196/1024, 19 %) have similar admission profile to those not lost; however, family characteristics differ, suggesting that those lost may have had higher socio-economic status than those remaining in the sample (see Annex Table 1). However, we feel that this presents low risk of bias since those family characteristics (mother died, parents’ level of education) are not associated with PMGr outcomes. Our results are also limited by the relatively young age at which NCD risk was assessed (median age 11 years). It could be that metabolic loads are not yet sufficient to manifest as risk factors for NCD. Both risk factors and NCD themselves will only become apparent as the cohort ages. Also important to acknowledge is that a risk factor for NCD does not necessarily mean that the NCD itself will develop^(47)^. Related to this, risk factors established in and applicable to high-income western populations may not apply equally to populations such as ours in Malawi^(48)^.

Since this is an observational analysis in which all children received similar diets, we cannot say from our data what would have happened had growth rate been controlled by different caloric regimes. There are many potentially confounding factors affecting weight gain that are not controlled for, such as breast-feeding status, diarrhoea and vomiting during treatment, and other concurrent illnesses and infections which would affect weight gain and survival^(49)^. Weight gain differences may also reflect individual variations in general health status and resilience to illness episodes. Lastly, our results may not be generalisable to current CMAM programmes nor to other setting, especially to those at a different stage of the nutrition transition, since the nutrition environment post-discharge is likely to have a large influence on later NCD risk factors^(50)^.

Addressing these limitations, we call for future intervention trials where different doses and types of therapeutic food for severe malnutrition are compared and where long-term as well as short-term benefits as well as risks are assessed. Valid measures, markers and biomarkers of long-term NCD risk are particularly important to develop since it is unlikely that any study will be able to follow up children to the point where NCD are directly measurable.

## Conclusion

Our study paints a complex picture of benefits and risks associated with faster PMGr. Different indicators of PMGr show different relationships with the outcomes, and our results indicate that simple measures of weight gain (g/d), initial growth deficit and the pattern of change have important implications for future health and NCD. To research this topic in the future, better understanding of NCD risk markers and biomarkers is needed; peripheral *v*. visceral weight distribution in particular requires further exploration.

## Supplementary material

For supplementary material accompanying this paper visit https://doi.org/10.1017/S1368980023000411. click here to view supplementary material
