# Supplementary material for: Post-malnutrition growth and its associations with child survival and non-communicable disease risk: a secondary analysis of the Malawi ‘ChroSAM’ cohort
Source: Public Health Nutr. 2023 Mar 6;26(8):1658–70. doi: 10.1017/S1368980023000411 (PMC10466107; doi:10.1017/S1368980023000411)
Supplement: Supplementary file 1 [file S1368980023000411sup001.pdf]

Annex Tables and Figures

Chrosam CHANGE study analysis

Annex Table 1: baseline demographics of those lost to follow up

	Survivors to 7 years post-discharge (n=320)	Loss to Follow Up (190)
**Basic Demographics**		
Median age /would be age (at 7 years post discharge) (years)	9.3 (range 7.4 – 20.1)	8 (range 7 - 19)
Males (gender)	174 (55%)	108 (57%)
Median Birth Order	2 (IQR 1-4)	2 (IQR 1-3)
**Clinical History**		
**HIV**		
Seropositive	90/320 (28%)	44/190(23%)
Seronegative	208/320(65%)	121/190(64%)
Unknown	22/320 (7%)	25/190 (13%)
History of TB	18/317 (5.7%)	4/173 (2%)
Weight for age z-score (mean (SD)) (minimum on ward)	-3.66 (1.39)	-3.66 (1.44)
Height for age z-score (mean (SD)) (minimum on ward)	-3.14 (1.43)	-3.13 (1.50)
**Family**		
Mother died	51/303 (17%)	8/159 (5%)
Mother HIV positive	83/222 (37%)	23/81 (28%)
**Socioeconomic status**		
Mother with no education	56/309 (18%)	13/155 (8%)
Father with no education	39/270 (14%)	5/122 (4%)

Annex Table 2: sample size for LCA-derived PMGr classes

LCA classes	WAZ LCA<br>N (%)	Weight LCA<br>N (%)	HAZ LCA<br>N (%)
Class 1	21 (6.7)	60 (18.8)	17 (5.4)
Class 2	54 (17.1)	111 (34.8)	96 (30.2)
Class 3	78 (24.7)	97 (30.4)	121 (38.1)
Class 4	53 (16.8)	38 (11.9)	75 (23.6)
Class 5	16 (5.1)	13 (4.1)	8 (2.5)
Class 6	94 (29.8)	N/A	N/A

Annex Figure 1: mean WAZ, weight, and HAZ over time

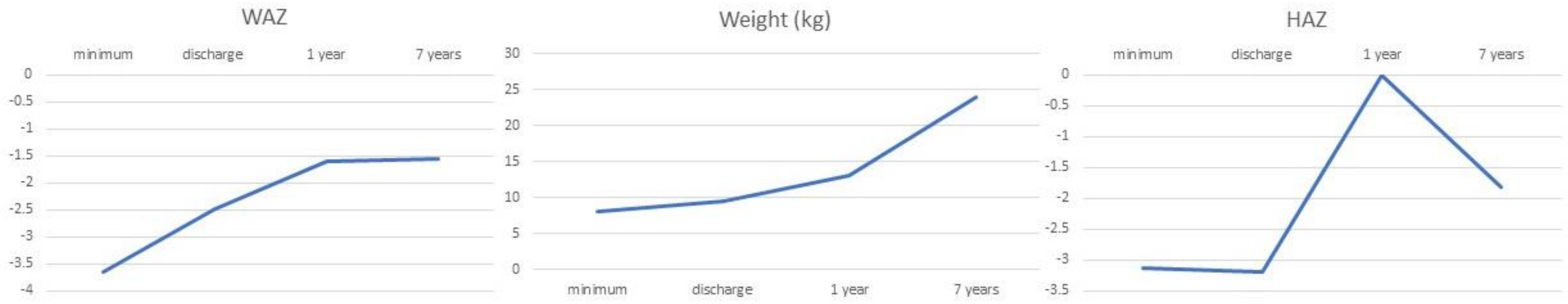

Annex Figure 2: Mean quintile weight-for-age z-scores (WAZ) according to five post-malnutrition growth rate definition (PMGr) at four (non-uniformly spaced) time point

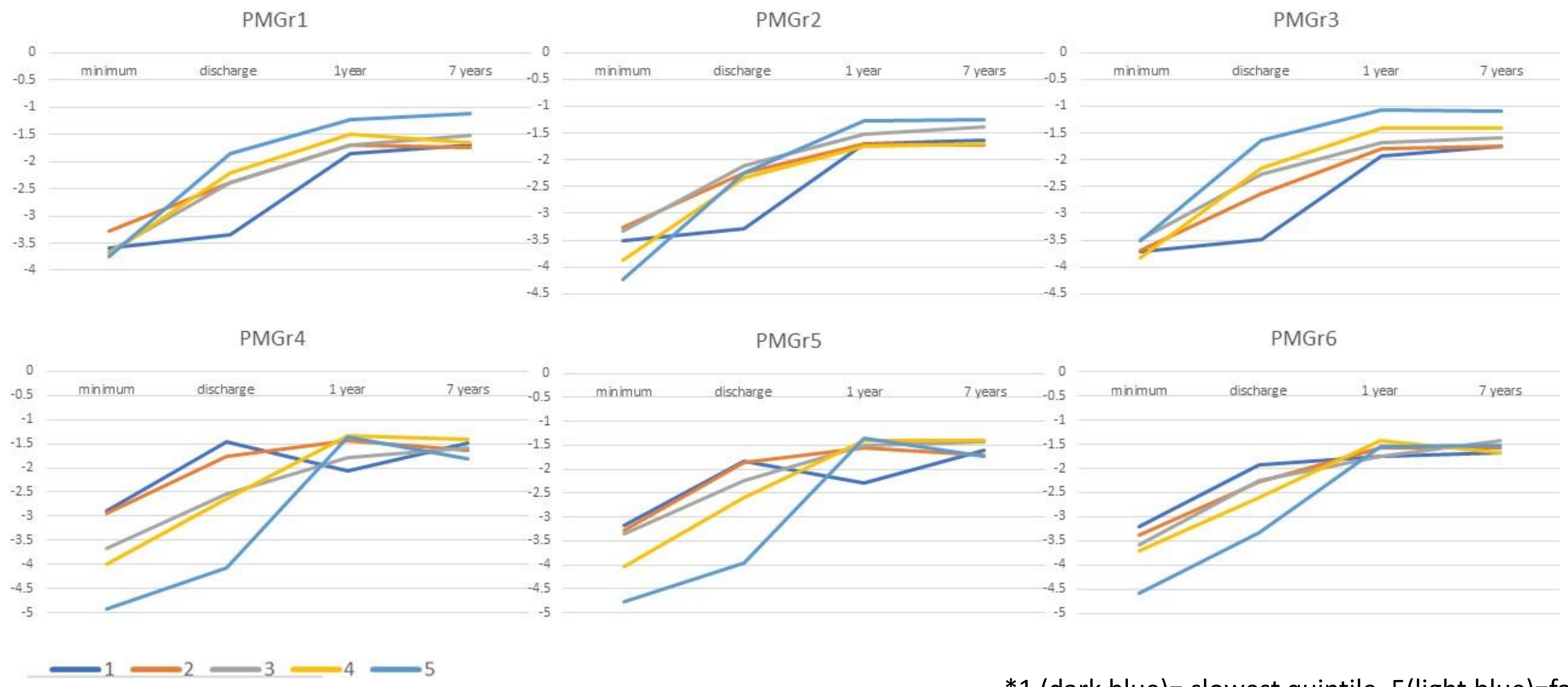

*1 (dark blue)= slowest quintile, 5(light blue)=fastest quintile

Annex Table 3: Association between PMGr definition quintiles and NCD outcomes

NCD indicator	PMGr definition	Unadjusted coefficient	P-value	95% CI	Adjusted coefficient	P-value	95% CI
Systolic BP	1	0.368	0.349	(-0.40, 1.14)	-0.061	0.877	(-0.84, 0.72)
	2	0.010	0.979	(-0.77, 0.79)	-0.072	0.847	(-0.80, 0.66)
	3	0.629	0.115	(-0.15, 1.41)	-0.158	0.682	(-0.92, 0.60)
	4	-1.081	0.012	(-1.92, -0.24)	-0.703	0.113	(-1.57, 0.17)
	5	-0.919	0.030	(-1.75, -0.09)	-0.373	0.400	(-1.25, 0.49)
	6	-0.164	0.703	(-1.01, 0.68)	-0.360	0.390	(-1.18, 0.46)
Diastolic BP	1	-0.247	0.506	(-098, 0.48)	-0.349	0.373	(-1.12, 0.42)
	2	-0.272	0.446	(-0.97, 0.43)	-0.281	0.440	(-0.99, 0.43)
	3	0.026	0.943	(-0.67, 0.73)	-0.234	0.535	(-0.97, 0.51)
	4	-0.936	0.023	(-1.74, -0.13)	-0.864	0.053	(-1.74, 0.01)
	5	-0.685	0.073	(-1.44, 0.07)	-0.689	0.117	(-1.55, 0.17)
	6	0.242	0.558	(-0.57, 1.05)	-0.024	0.954	(-0.86, 0.81)
Hand grip	1	0.106	0.488	(-0.19, 0.41)	0.037	0.797	(-0.25, 0.32)
	2	-0.100	0.543	(-0.43, 0.22)	0.000	0.999	(-0.28, 0.28)
	3	0.365	0.027	(0.04, 0.69)	0.098	0.508	(-0.19, 0.39)
	4	-0.155	0.329	(-0.47, 0.16)	0.093	0.562	(-0.22, 0.41)
	5	-0.459	0.010	(-0.81, -0.11)	0.079	0.643	(-0.26, 0.42)
	6	0.295	0.064	(-0.12, 0.61)	0.278	0.069	(-0.02, 0.58)
Waist circumference	1	0.332	0.042	(0.01, 0.65)	0.316	0.047	(0.00, 0.63)
	2	0.109	0.534	(-0.24, 0.45)	0.253	0.099	(-0.05, 0.55)
	3	0.604	0.001	(0.27, 0.94)	0.369	0.018	(0.06, 0.68)
	4	-0.375	0.022	(-0.69, -0.06)	-0.249	0.137	(-0.58, 0.08)
	5	-0.474	0.008	(-0.82, -0.12)	0.059	0.733	(-0.28, 0.39)
	6	-0.214	0.189	(-0.54, 0.11)	-0.299	0.059	(-0.61, 0.01)

NCD indicator	PMGr definition	Unadjusted coefficient	P-value	95% CI	Adjusted coefficient	P-value	95% CI
Waist:Hip ratio	1	-0.000	0.923	(-0.01, 0.01)	0.001	0.796	(-0.00, 0.01)
	2	0.002	0.441	(-0.00, 0.01)	0.002	0.382	(-0.00, 0.01)
	3	-0.002	0.409	(-0.01, 0.00)	0.000	0.993	(-0.01, 0.01)
	4	0.004	0.155	(-0.00, 0.01)	-0.000	0.989	(-0.01, 0.01)
	5	0.005	0.061	(-0.00, 0.01)	0.001	0.672	(-0.00, 0.01)
	6	-0.004	0.182	(-0.01, 0.00)	-0.004	0.147	(-0.01, 0.00)
Lean mass index (BIA)	1	0.138	0.056	(-0.00, 0.28)	0.109	0.143	(-0.04, 0.26)
	2	0.039	0.577	(-0.10, 0.18)	0.041	0.559	(-0.09, 0.18)
	3	0.136	0.057	(-0.00, 0.28)	0.067	0.356	(-0.08, 0.21)
	4	-0.111	0.168	(-0.27, 0.05)	-0.071	0.423	(-0.24, 0.10)
	5	-0.141	0.067	(-0.29, 0.01)	-0.015	0.860	(-0.18, 0.15)
	6	-0.049	0.543	(-0.21, 0.11)	-0.026	0.755	(-0.19, 0.14)
Fat mass index (BIA)	1	-0.012	0.800	(-0.11, 0.08)	0.005	0.923	(-0.09, 0.11)
	2	-0.024	0.623	(-0.12, 0.07)	0.006	0.901	(-0.09, 0.09)
	3	0.082	0.094	(-0.01, 0.18)	0.065	0.186	(-0.03, 0.16)
	4	-0.019	0.711	(-0.12, 0.08)	-0.033	0.568	(-0.15, 0.08)
	5	-0.061	0.227	(-0.16, 0.04)	-0.018	0.746	(-0.13, 0.09)
	6	0.051	0.337	(-0.05, 0.16)	0.006	0.910	(-0.10, 0.11)
HAZ	1	0.115	0.019	(0.02, 0.21)	0.091	0.066	(-0.01, 0.19)
	2	0.083	0.090	(-0.01, 0.18)	0.051	0.281	(-0.04, 0.14)
	3	0.049	0.310	(-0.05, 0.15)	0.097	0.05	(0.00, 0.19)
	4	-0.023	0.663	(-0.12, 0.08)	-0.013	0.819	(-0.12, 0.09)
	5	0.141	0.007	(0.04, 0.24)	0.089	0.114	(-0.02, 0.20)
	6	-0.027	0.599	(-0.13, 0.07)	0.016	0.757	(-0.09, 0.12)

Annex Figures 3a-1h

Scatterplots showing associations between ‘no categorised’ PMGr indicators and NCD risk outcomes

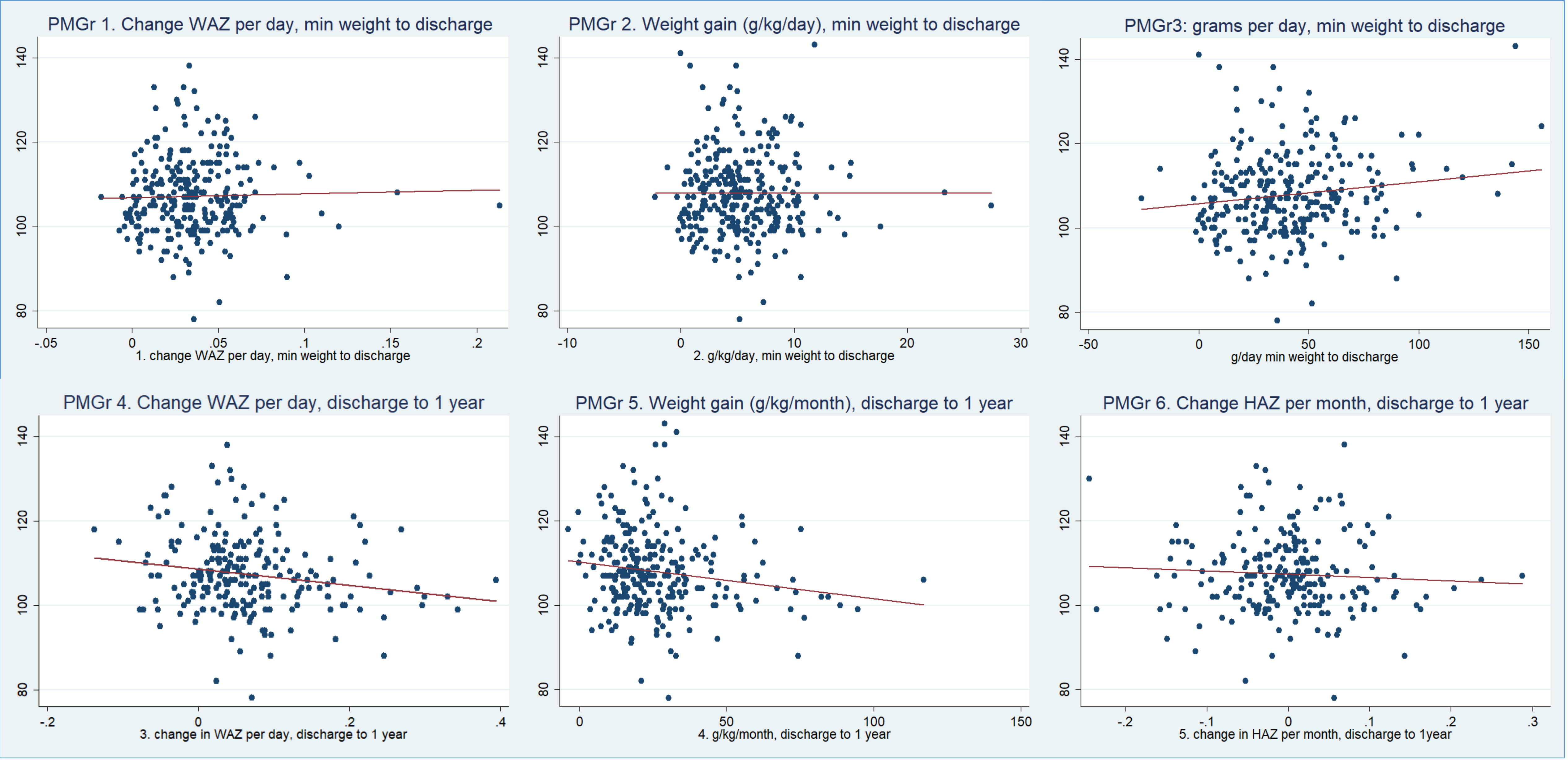

Systolic Blood Pressure

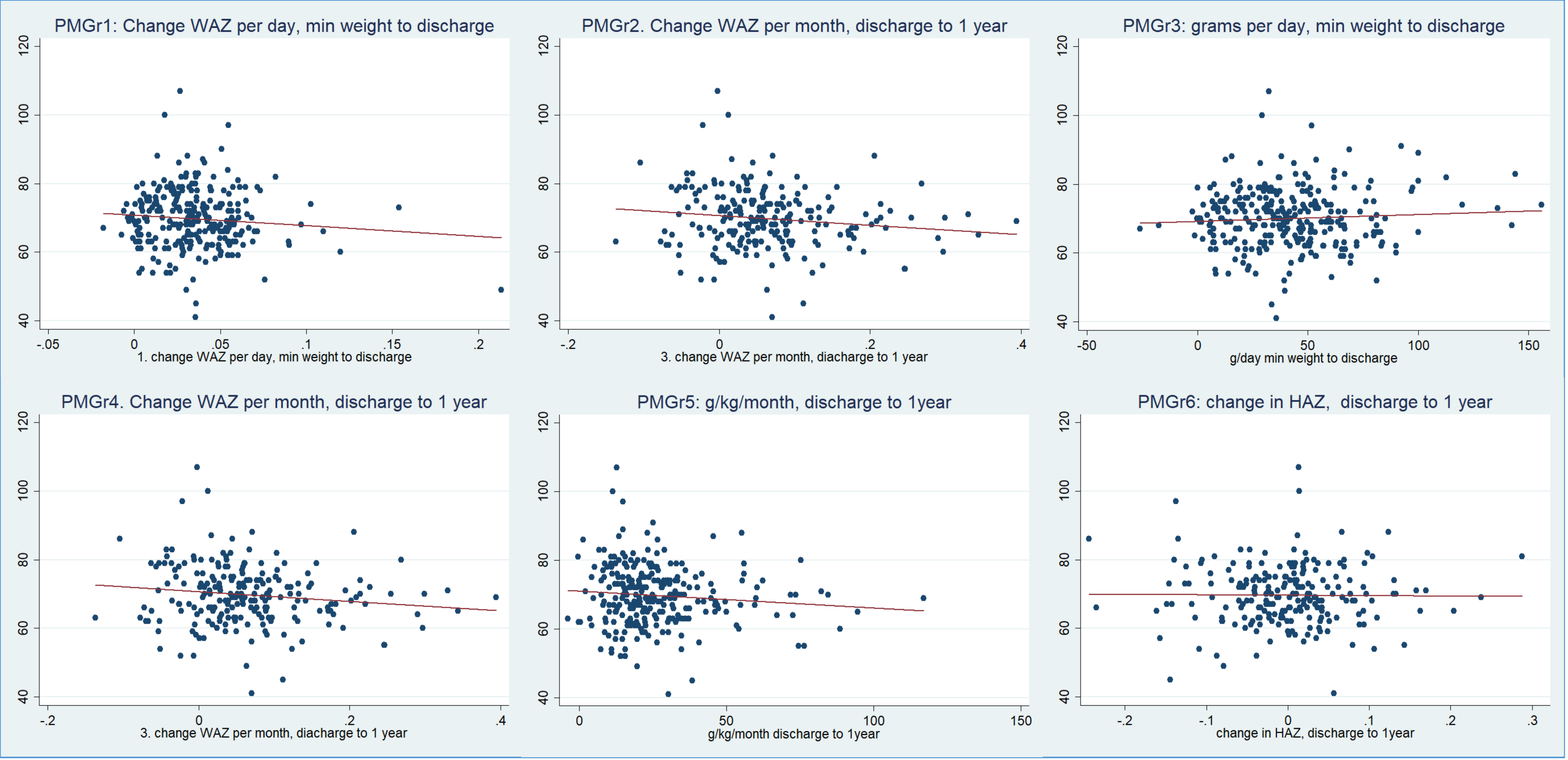

Diastolic Blood Pressure

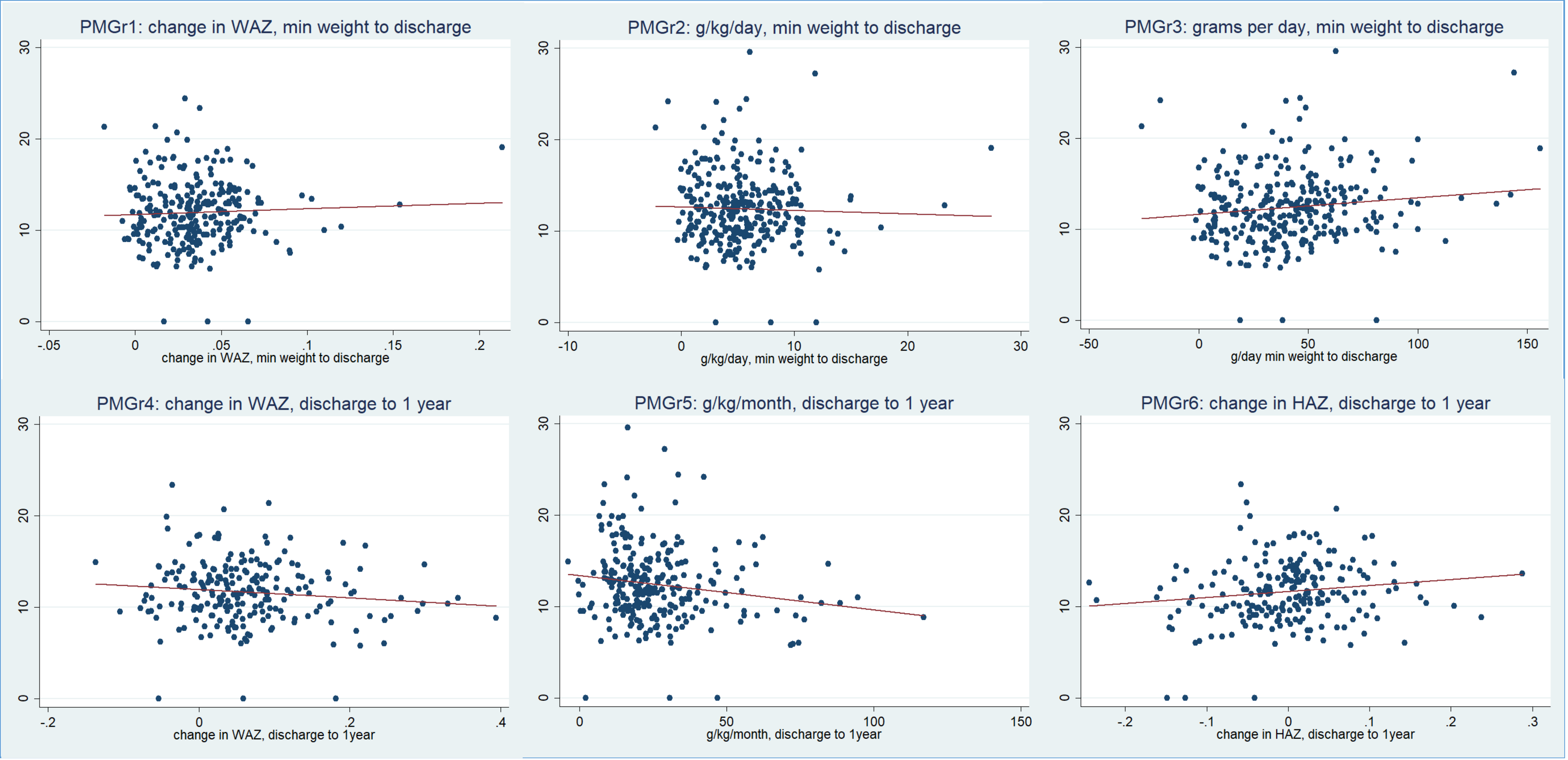

Hand grip strength

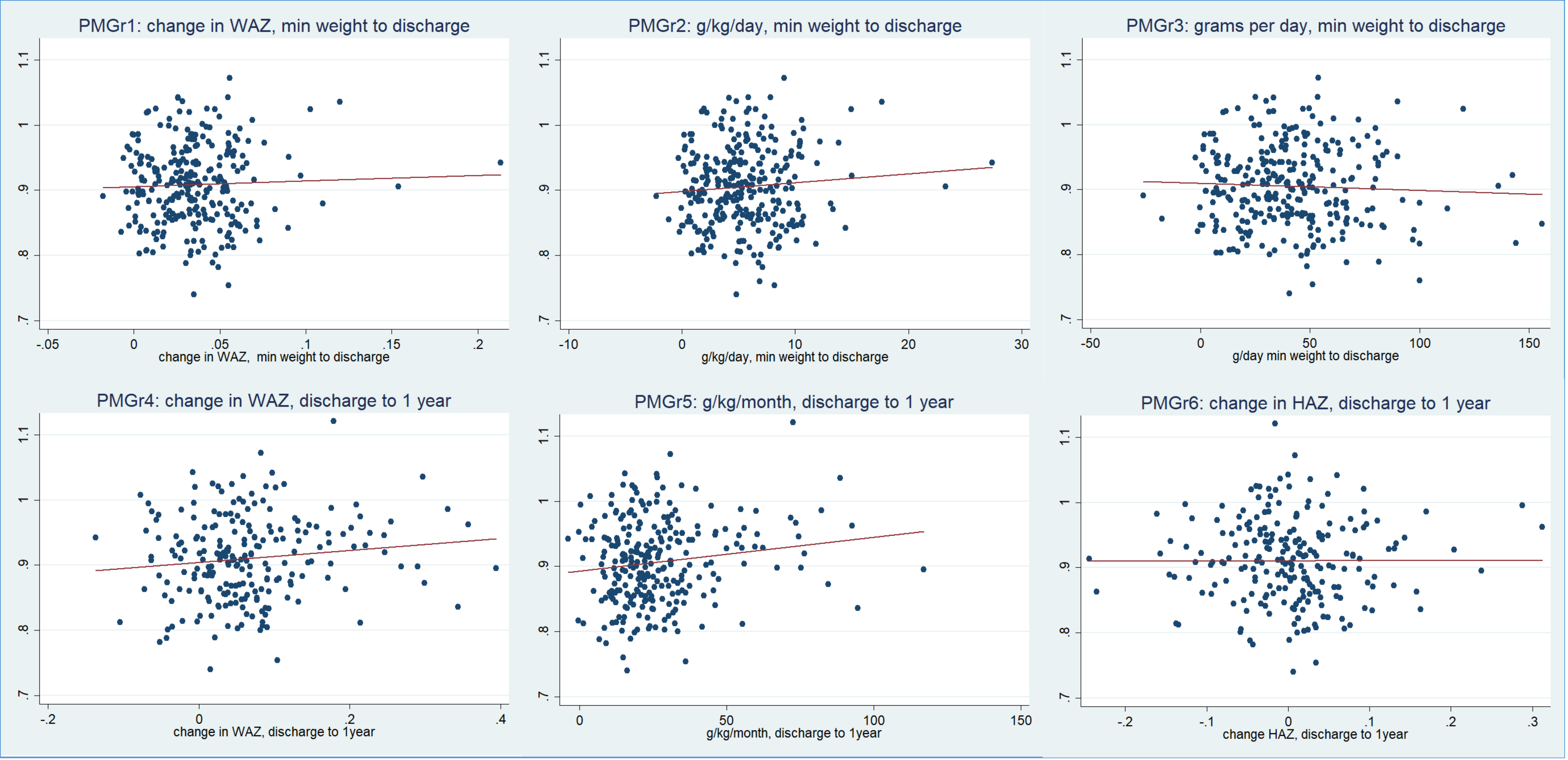

Waist/Hip ratio

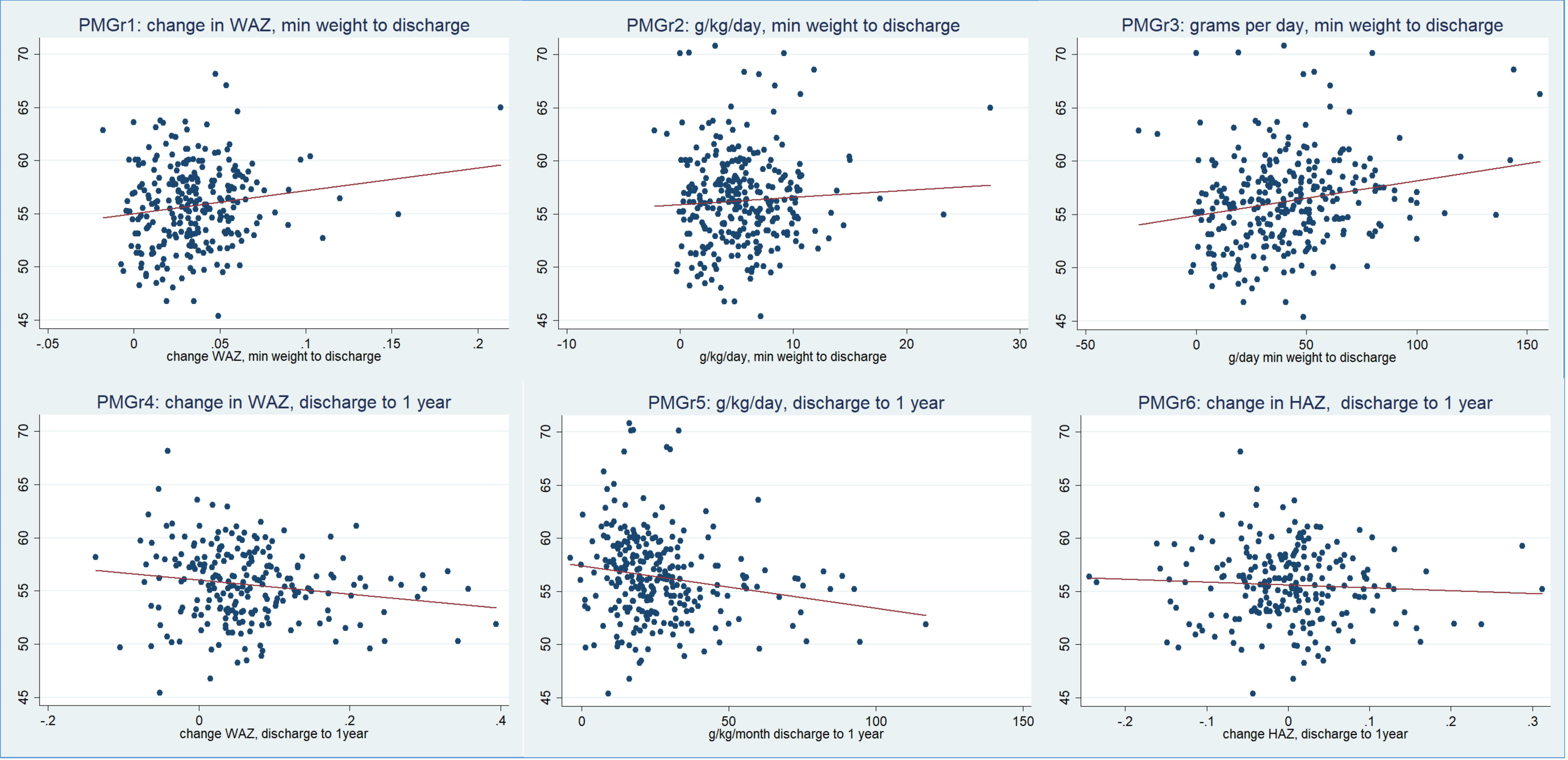

Waist circumference

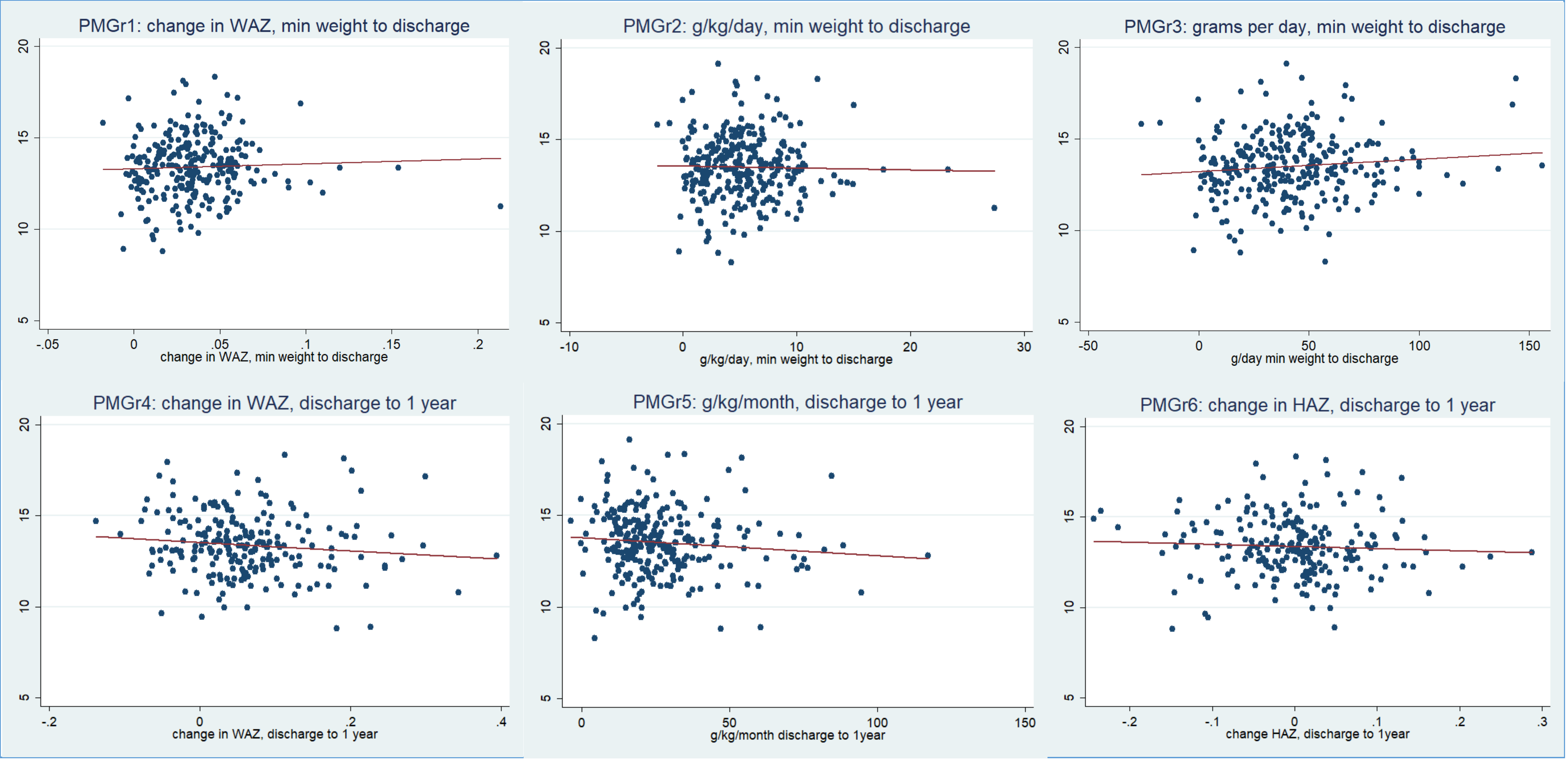

Lean Mass Index

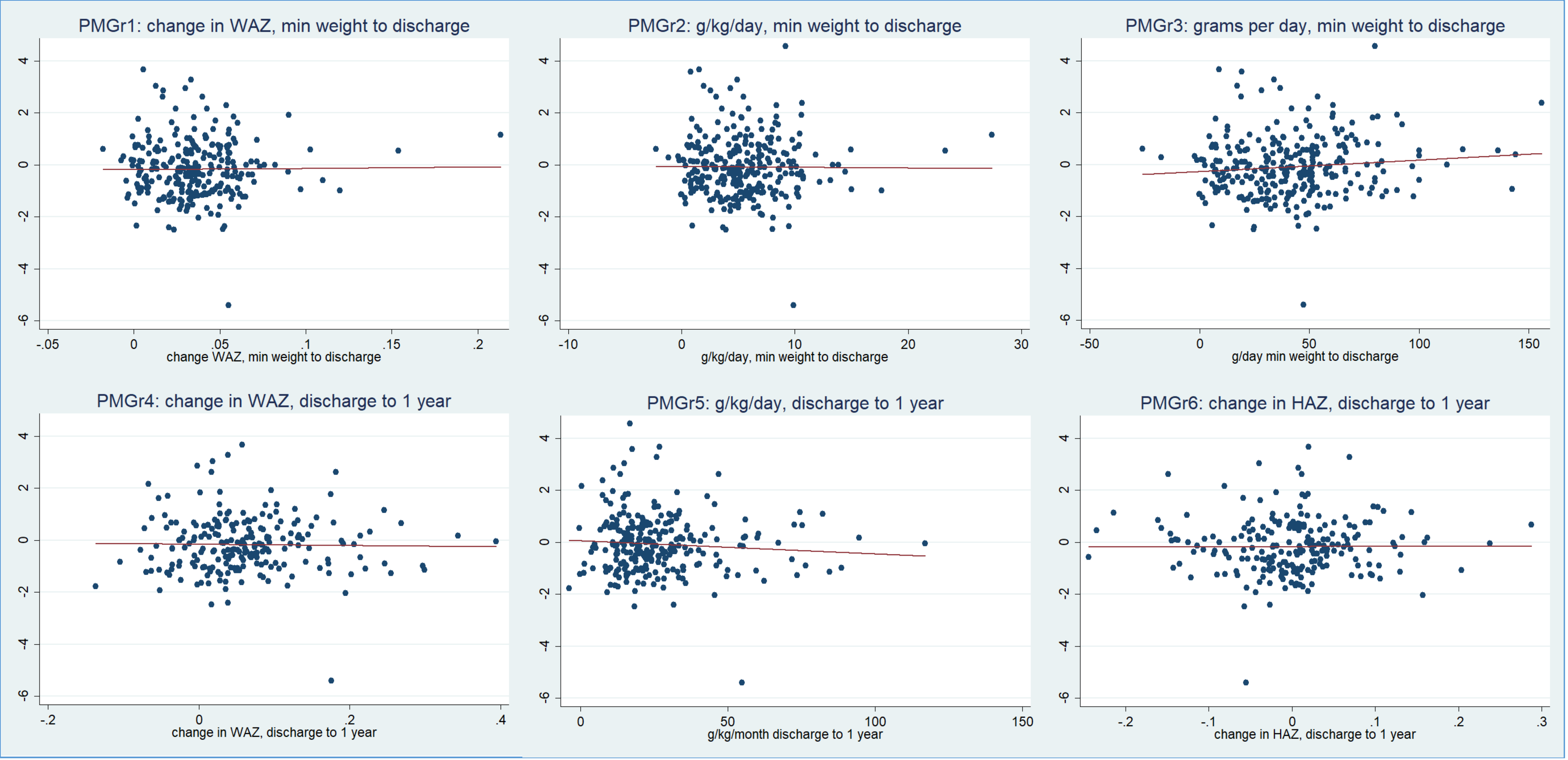

Fat Mass Index

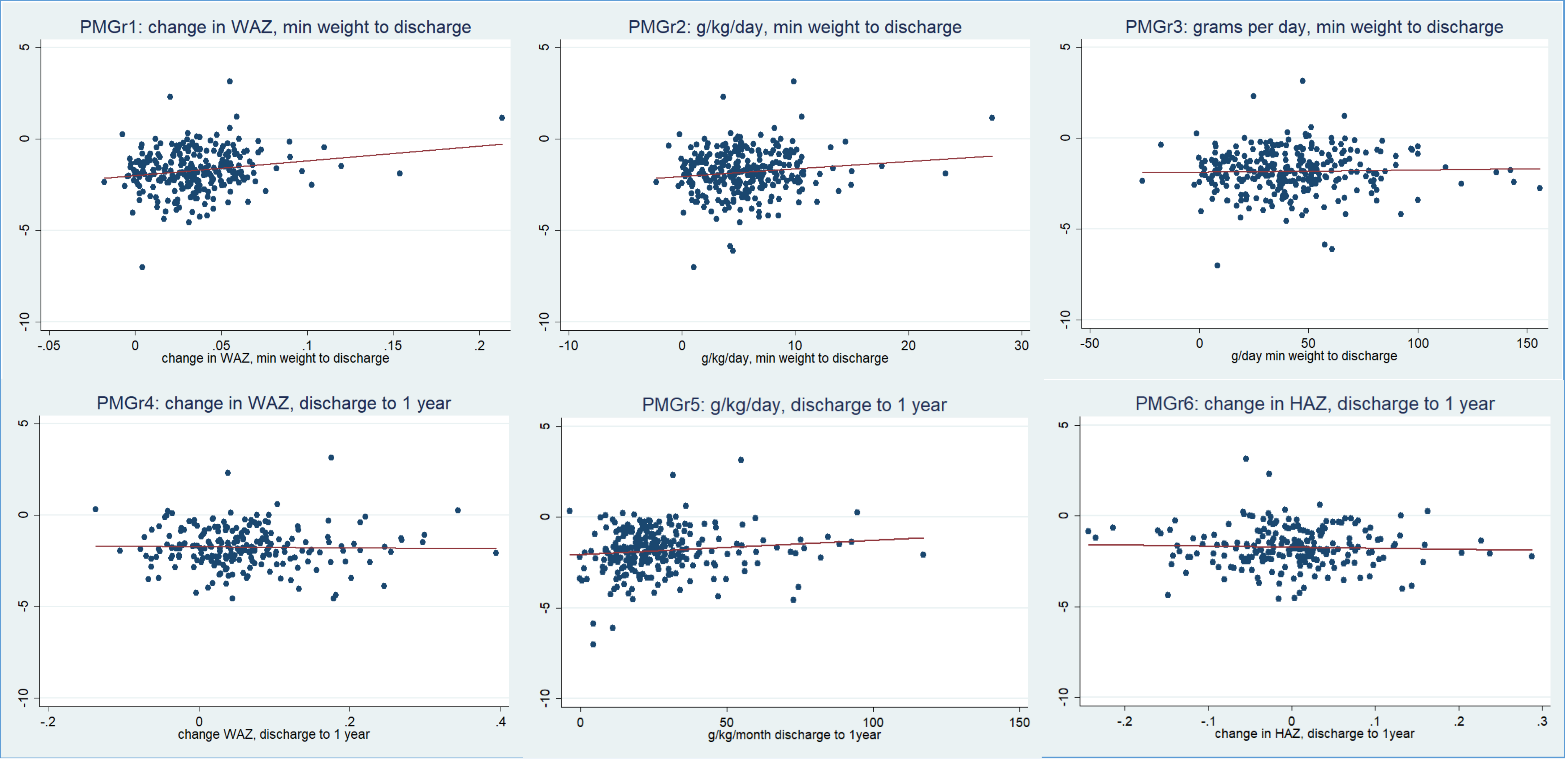
PMGr1: change in WAZ, min weight to discharge
change in WAZ, min weight to discharge
PMGr2: g/kg/day, min weight to discharge
g/kg/day, min weight to discharge
PMGr3: grams per day, min weight to discharge
g/day min weight to discharge
PMGr4: change in WAZ, discharge to 1 year
change WAZ, discharge to 1 year
PMGr5: g/kg/day, discharge to 1 year
g/kg/month discharge to 1year
PMGr6: change in HAZ, discharge to 1 year
change in HAZ, discharge to 1year

HAZ

Annex Figures 4a-2h

Boxplots showing associations between PMGr quintiles and NCD risk outcomes

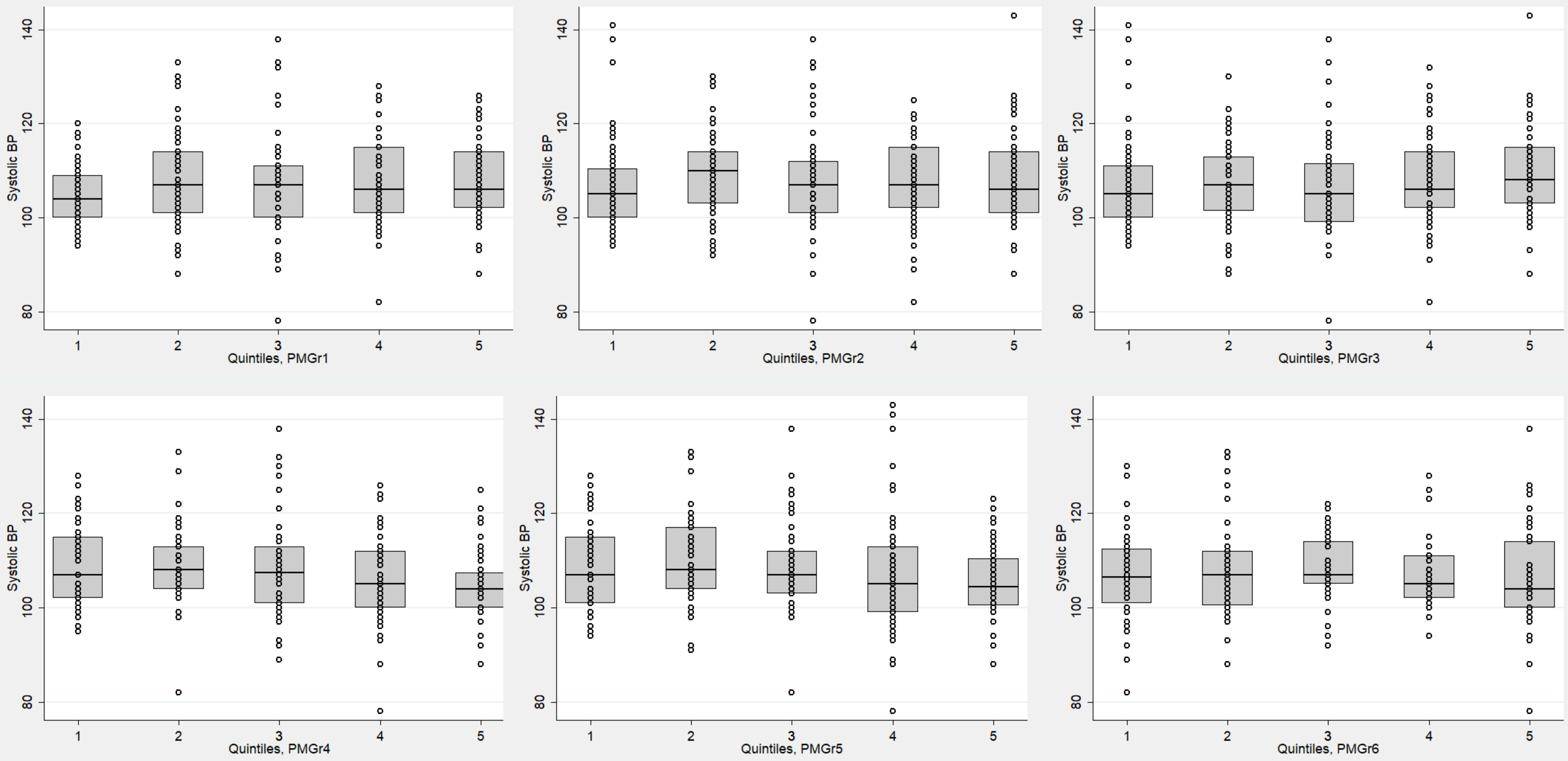
Systolic BP
140
120
100
80
1
2
3
4
5
Quintiles, PMGr1
Quintiles, PMGr2
Quintiles, PMGr3
Quintiles, PMGr4
Quintiles, PMGr5
Quintiles, PMGr6

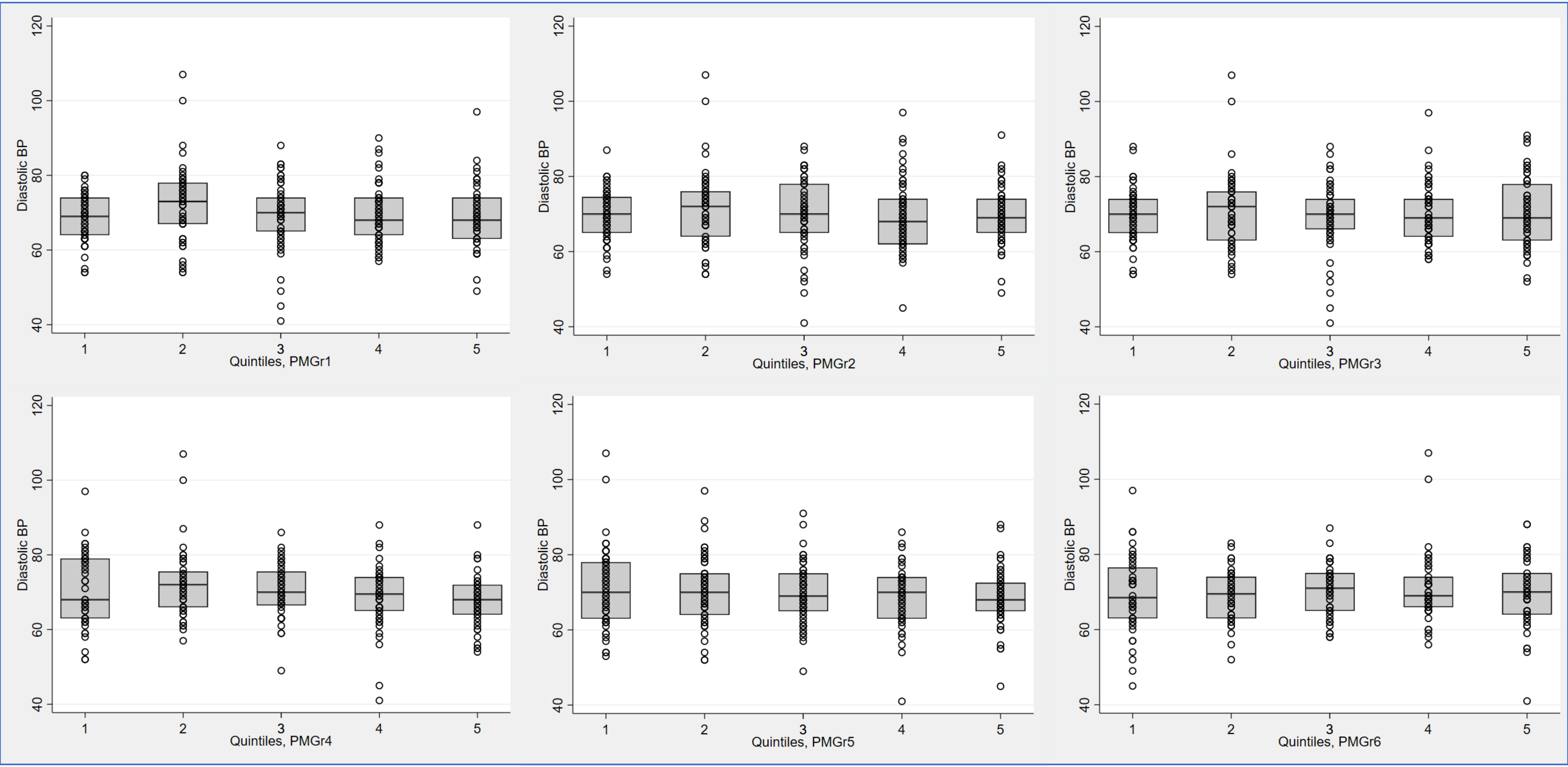

Diastolic BP
120
100
80
60
40
1
2
3
4
5
Quintiles, PMGr1
Quintiles, PMGr2
Quintiles, PMGr3
Quintiles, PMGr4
Quintiles, PMGr5
Quintiles, PMGr6

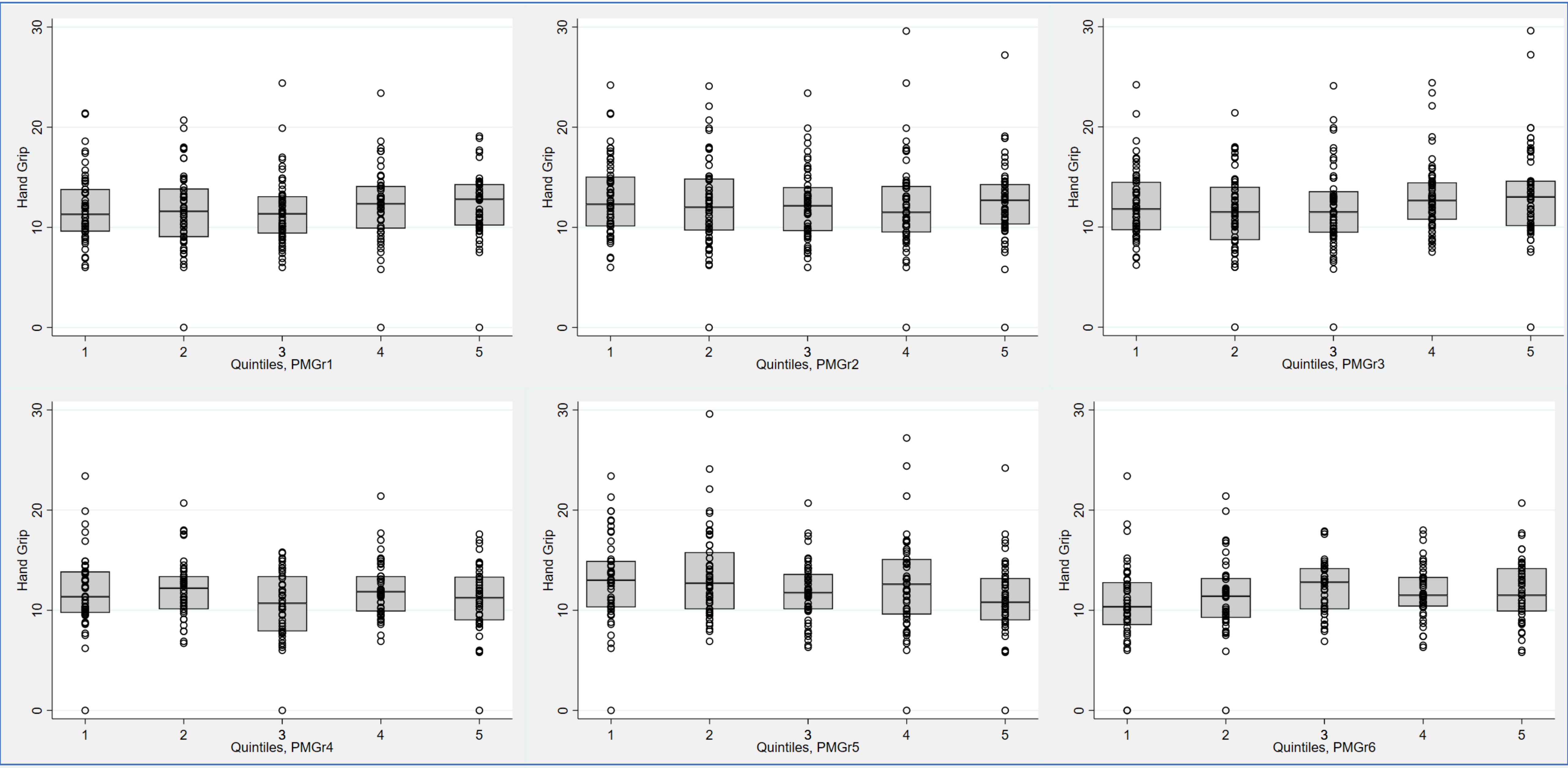

Hand Grip
Quintiles, PMGr1
Quintiles, PMGr2
Quintiles, PMGr3
Quintiles, PMGr4
Quintiles, PMGr5
Quintiles, PMGr6

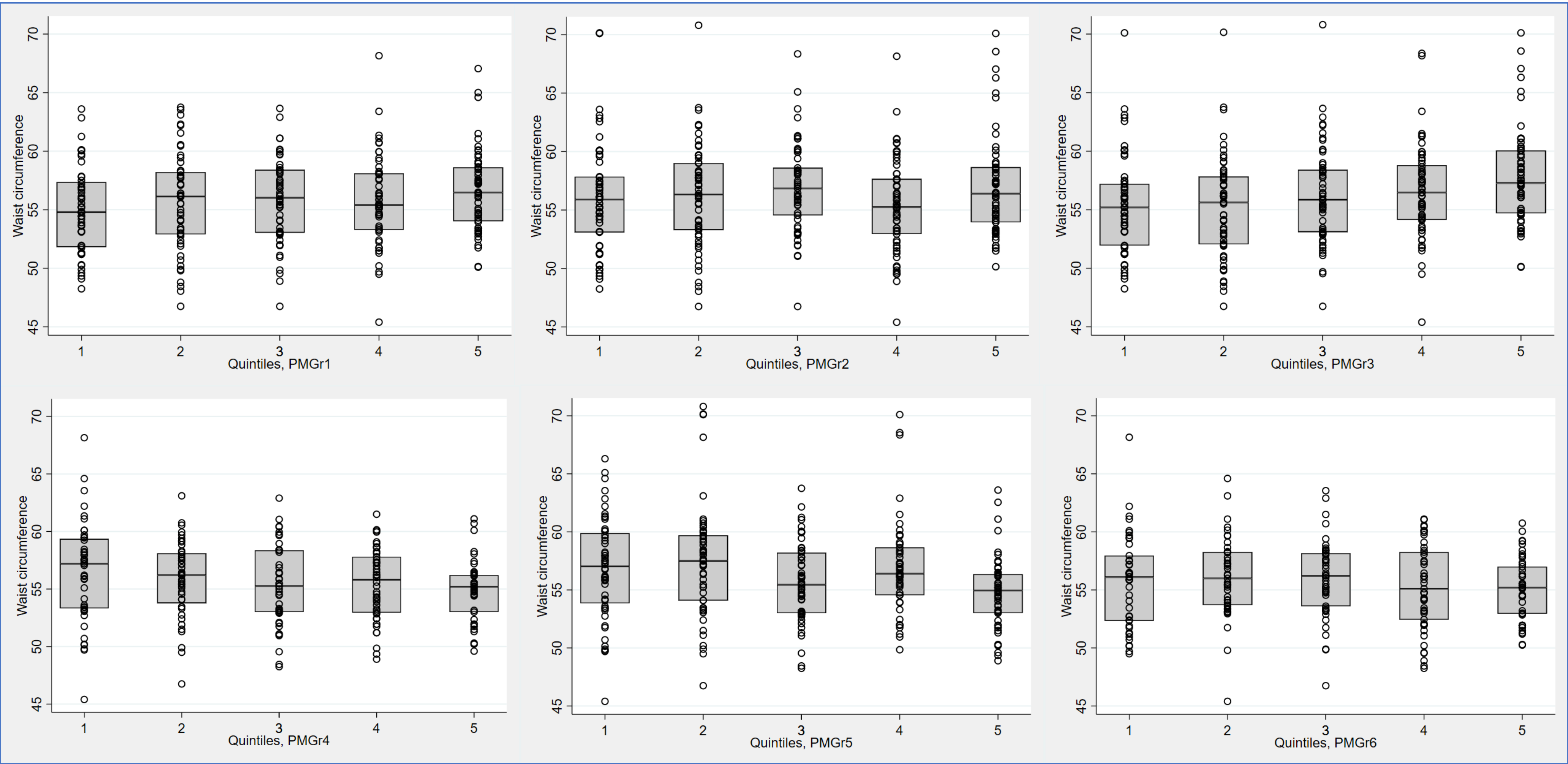
Waist circumference
Quintiles, PMGr1
Quintiles, PMGr2
Quintiles, PMGr3
Quintiles, PMGr4
Quintiles, PMGr5
Quintiles, PMGr6
45
50
55
60
65
70
1
2
3
4
5

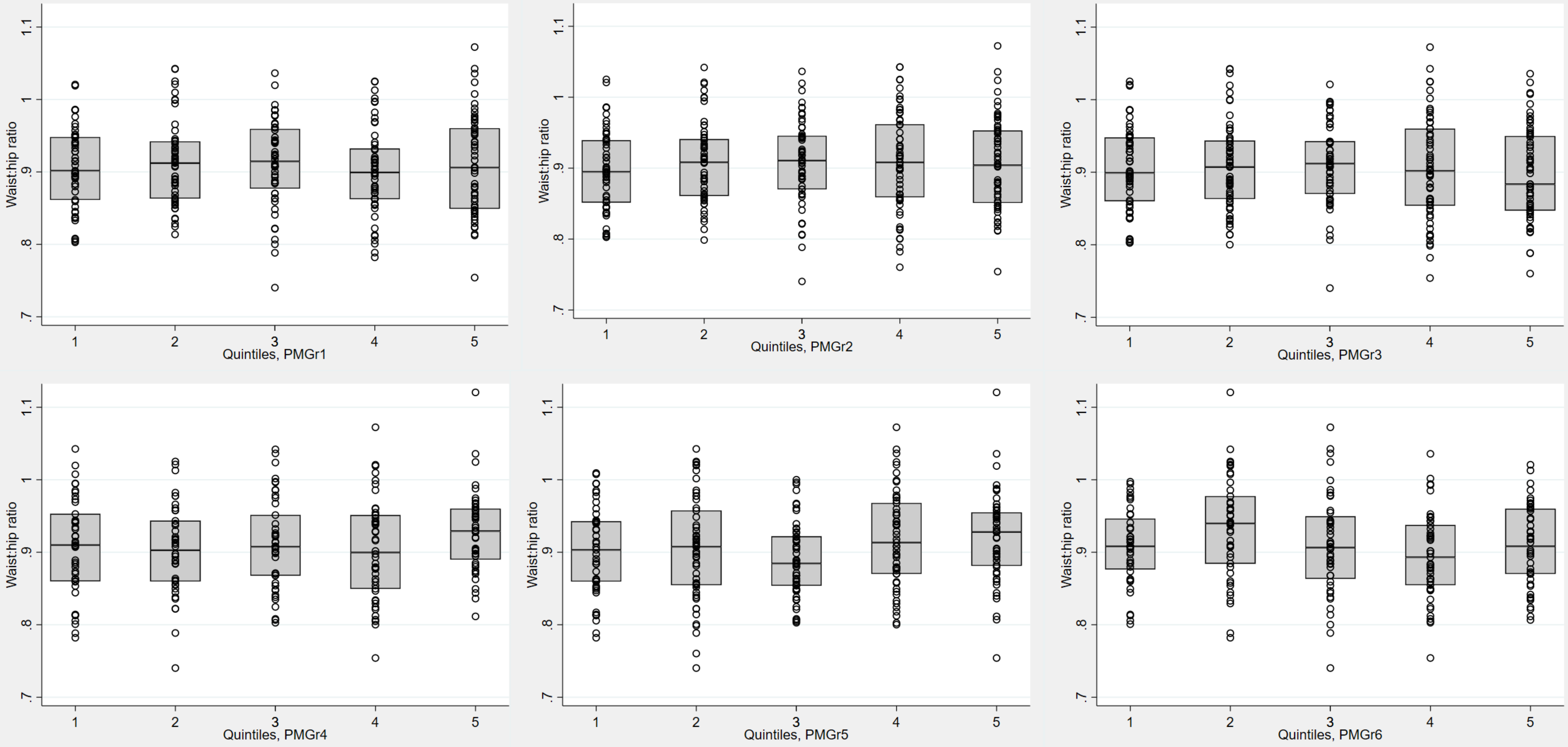

Waist:hip ratio
.7
.8
.9
1
1.1
1
2
3
4
5
Quintiles, PMGr1
Quintiles, PMGr2
Quintiles, PMGr3
Quintiles, PMGr4
Quintiles, PMGr5
Quintiles, PMGr6

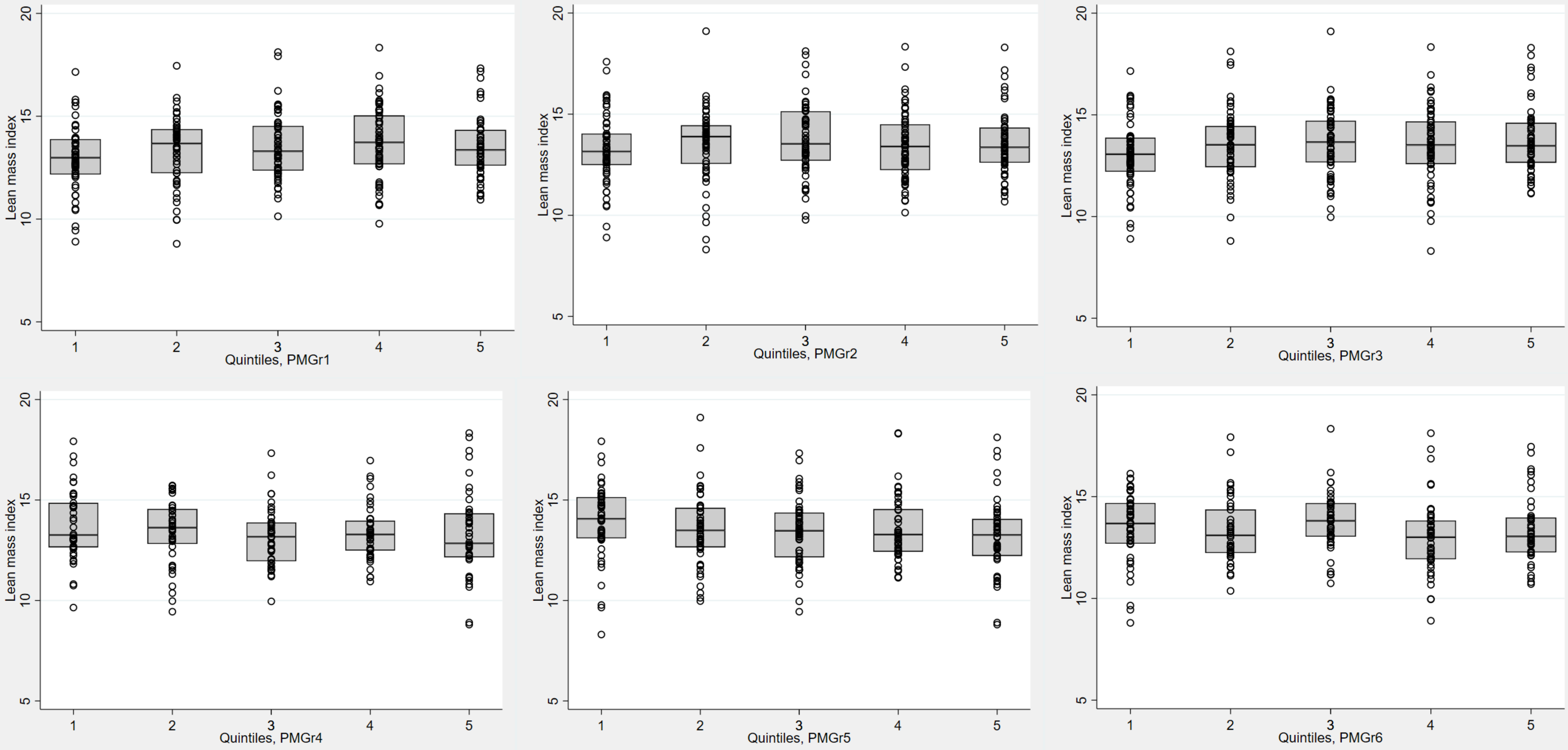
Lean mass index
Quintiles, PMGr1
Quintiles, PMGr2
Quintiles, PMGr3
Quintiles, PMGr4
Quintiles, PMGr5
Quintiles, PMGr6

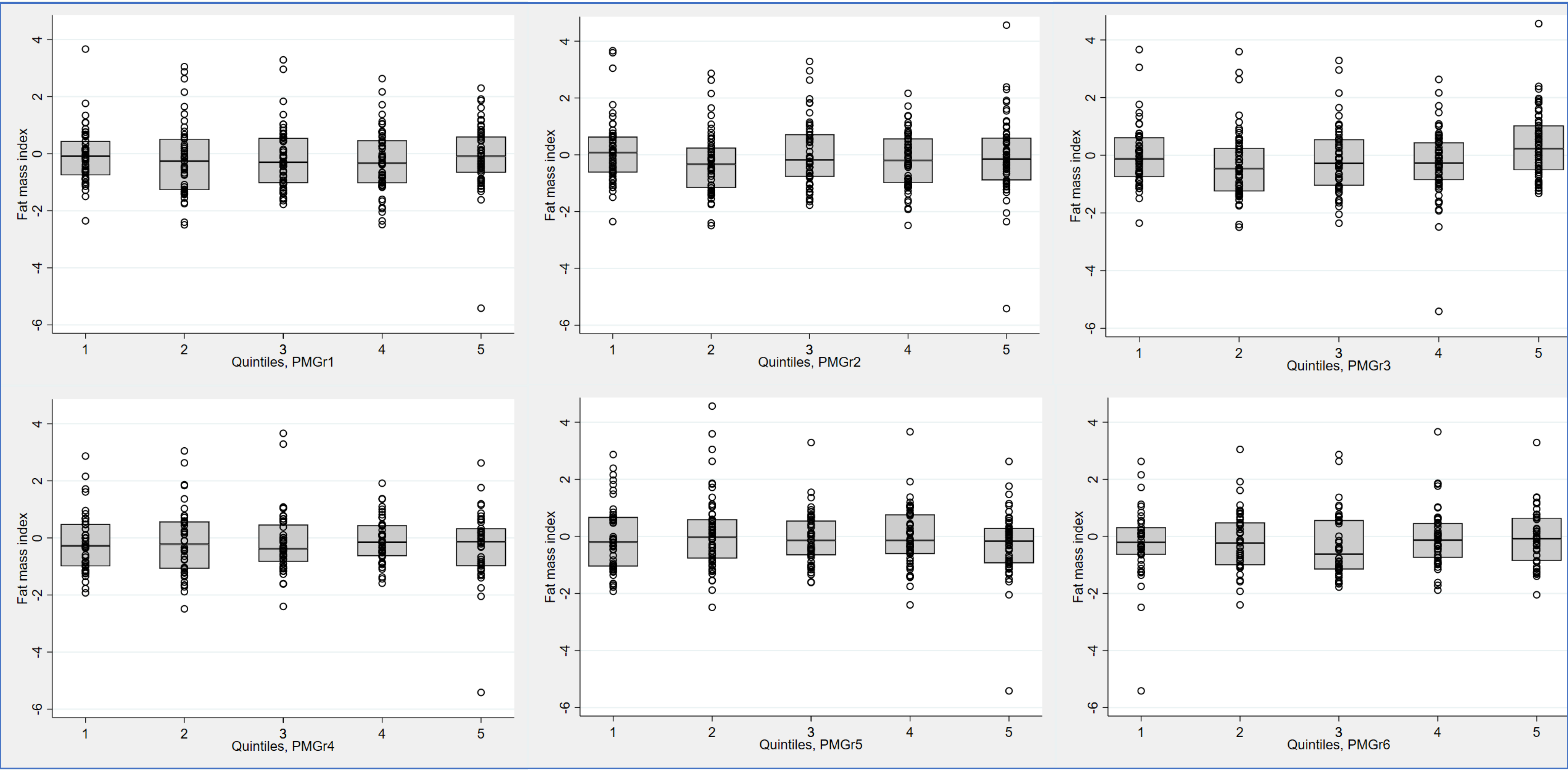
Fat mass index
Quintiles, PMGr1
Quintiles, PMGr2
Quintiles, PMGr3
Quintiles, PMGr4
Quintiles, PMGr5
Quintiles, PMGr6

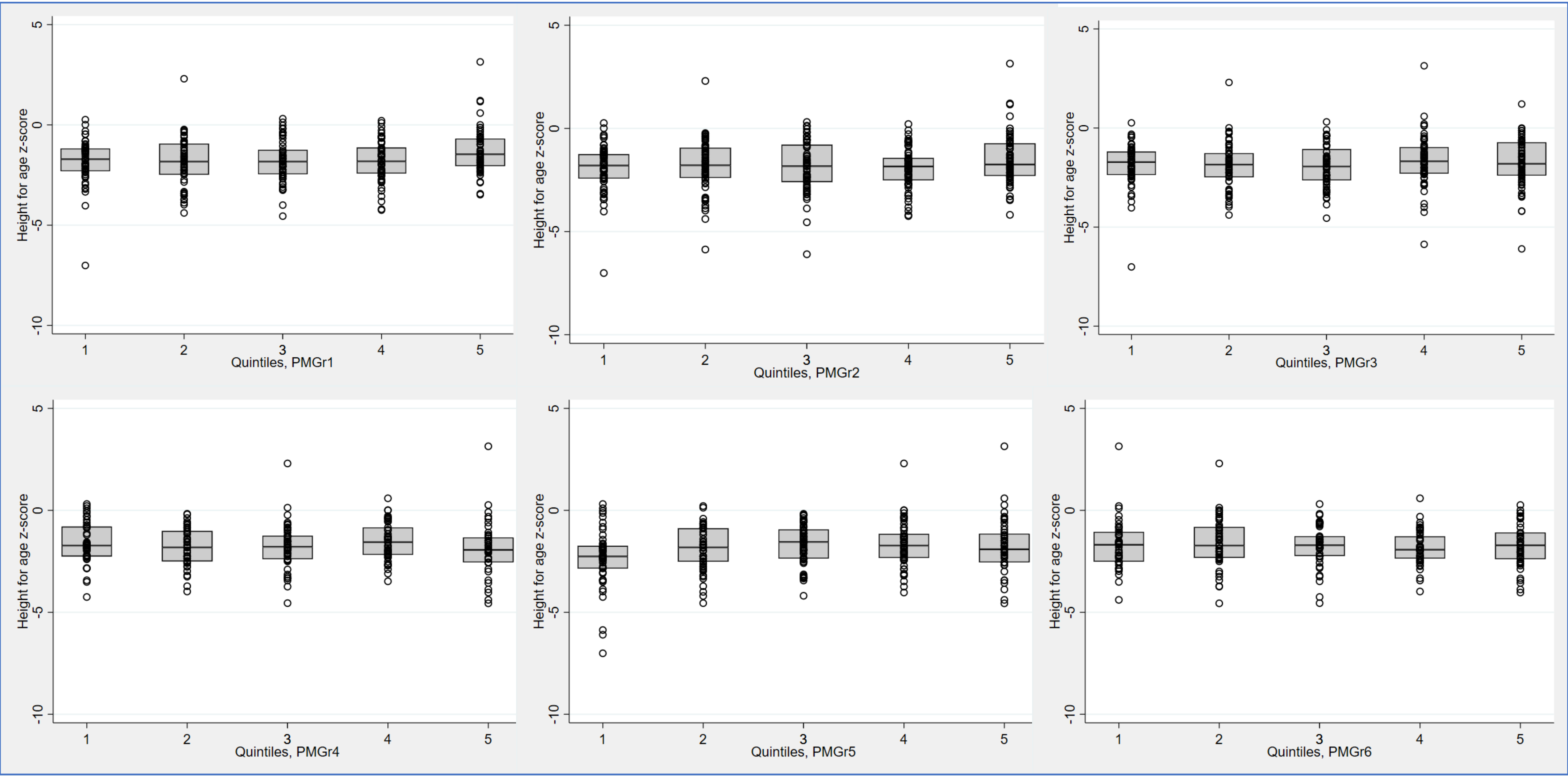
Height for age z-score
Quintiles, PMGr1
Quintiles, PMGr2
Quintiles, PMGr3
Quintiles, PMGr4
Quintiles, PMGr5
Quintiles, PMGr6

Annex Table 4: Association between LCA classes and NCD outcomes

NCD indicator	PMGr definition	Unadjusted difference	P-value	95% CI	Adjusted difference	P-value	95% CI
Systolic BP	WAZ	0.6414	0.140	(-0.21, 1.49)	-0.2037	0.645	(-1.07, 0.67)
	Weight (kg)	2.3708	<0.001	(1.38, 3.36)	-0.3867	0.593	(-1.81, 1.04)
	HAZ	1.1814	0.049	(0.01, 2.36)	0.0577	0.930	(-1.24, 1.36)
Diastolic BP	WAZ	0.2809	0.478	(-0.49, 1.06)	0.3101	0.478	(-0.55, 1.17)
	Weight (kg)	0.9327	0.049	(0.00, 1.86)	0.5185	0.469	(-0.89, 1.92)
	HAZ	-0.3271	0.548	(-1.39, 0.74)	-0.4877	0.453	(-1.77, 0.79)
Hand grip	WAZ	0.5679	0.002	(0.21, 0.82)	0.5780	<0.001	(0.27, 0.89)
	Weight (kg)	1.9069	<0.001	(1.54, 2.28)	0.9918	<0.001	(0.17, 1.51)
	HAZ	0.6217	0.014	(0.13, 1.12)	0.9560	<0.001	(0.53, 1.38)
Waist circumference	WAZ	0.8542	<0.001	(0.49, 1.21)	0.9525	<0.001	(0.63, 1.27)
	Weight (kg)	2.0531	0.000	(1.66, 2.44)	1.1631	<0.001	(0.62, 1.71)
	HAZ	0.7906	0.003	(0.28, 1.31)	1.2171	<0.001	(0.78, 1.66)

NCD indicator	PMGr definition	Unadjusted difference	P-value	95% CI	Adjusted difference	P-value	95% CI
Waist:Hip ratio	WAZ	-0.0105	<0.001	(-0.02, -0.01)	-0.0094	0.001	(-0.01, -0.00)
	Weight (kg)	-0.0152	<0.001	(-0.02, -0.01)	-0.0135	0.004	(-0.02, -0.00)
	HAZ	-0.0136	<0.001	(-0.02, -0.01)	-0.0150	<0.001	(-0.02, -0.01)
Lean mass index (BIA)	WAZ	0.2927	<0.001	(0.14, 0.44)	0.2889	<0.001	(0.13, 0.44)
	Weight (kg)	0.4648	<0.001	(0.29, 0.64)	0.4182	0.002	(0.16, 0.68)
	HAZ	0.1614	0.139	(-0.05, 0.38)	0.1949	0.076	(-0.02, 0.41)
Fat mass index (BIA)	WAZ	0.1300	0.014	(0.03, 0.23)	0.1667	0.002	(0.06, 0.27)
	Weight (kg)	0.2919	<0.001	(0.17, 0.41)	0.1933	0.032	(0.02, 0.37)
	HAZ	0.0369	0.621	(-0.11, 0.18)	0.1105	0.135	(-0.03, 0.26)
HAZ	WAZ	0.3202	<0.001	(0.22, 0.42)	0.3144	<0.001	(0.21, 0.41)
	Weight (kg)	-0.0746	0.263	(-0.21, 0.06)	0.3719	<0.001	(0.19, 0.54)
	HAZ	0.6768	<0.001	(0.55, 0.81)	0.6358	<0.001	(0.51, 0.76)

Annex Figures 5a-2h

Boxplots showing associations between LCA classes for weight, WAZ and HAZ, and NCD risk outcomes

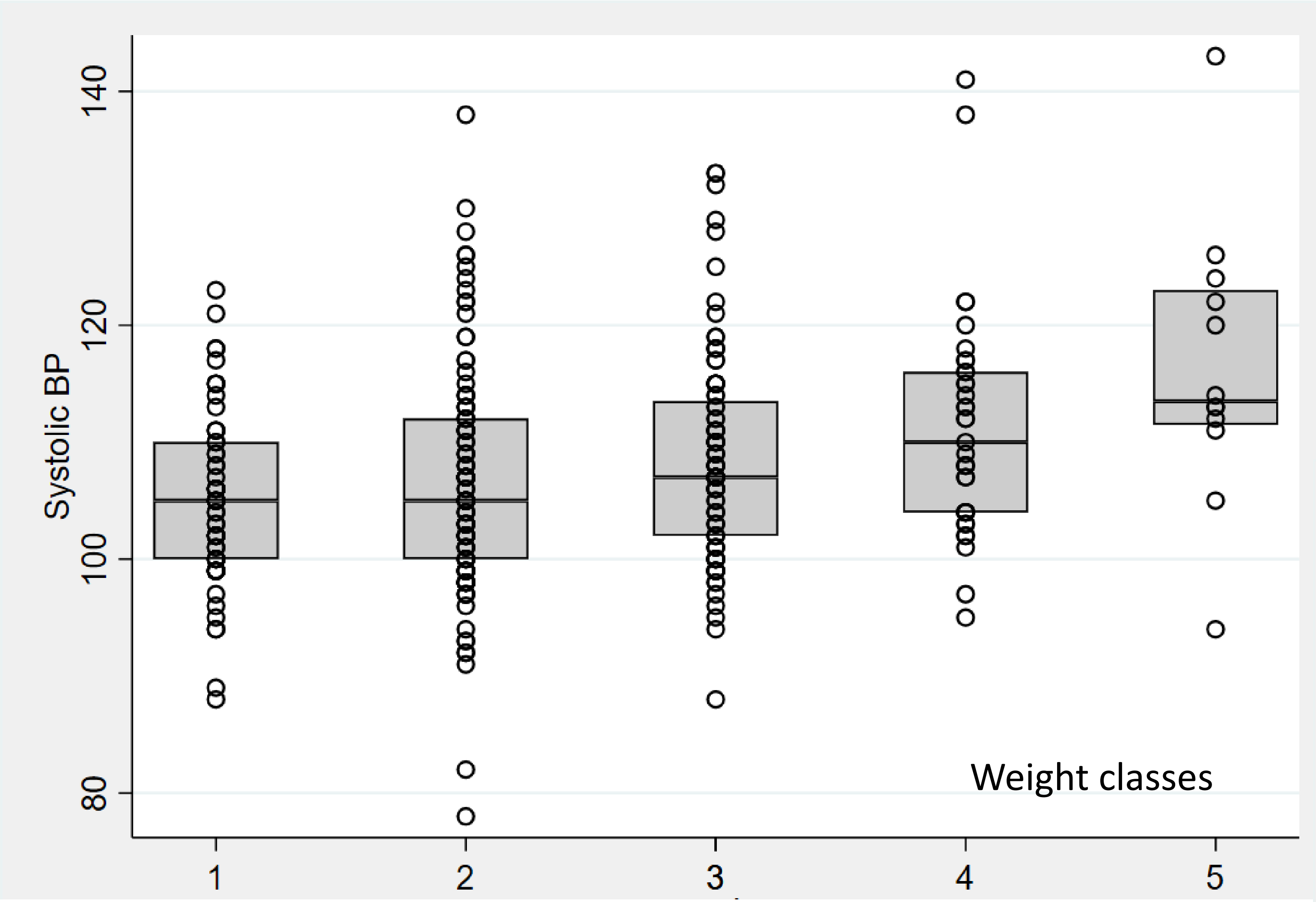
Systolic BP
140
120
100
80
1
2
3
4
5
Weight classes

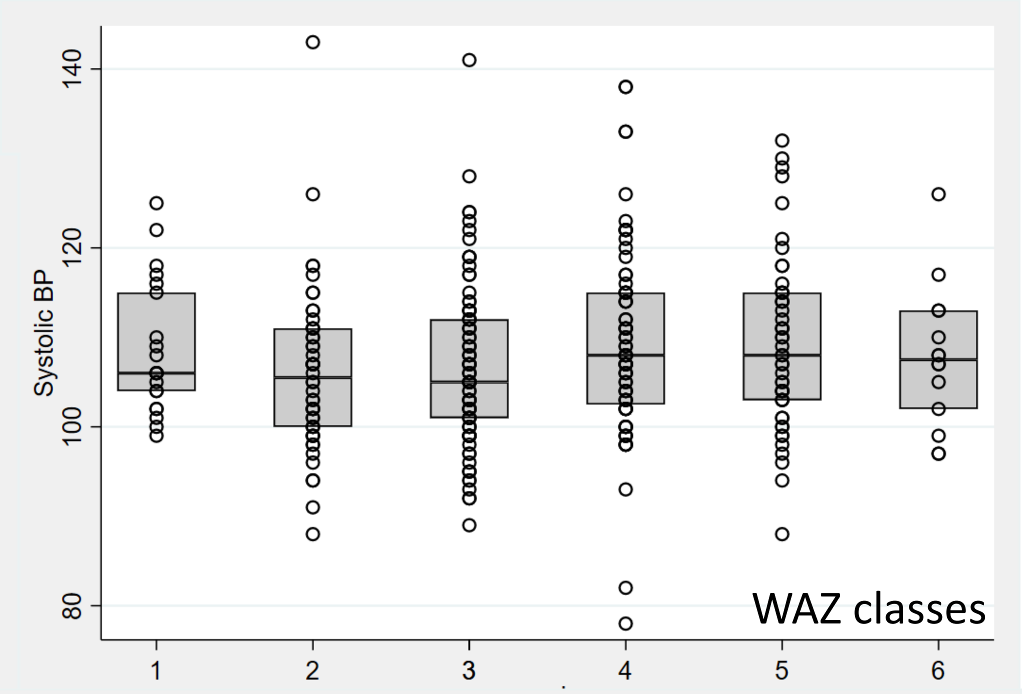
Systolic BP
140
120
100
80
1
2
3
4
5
6
WAZ classes

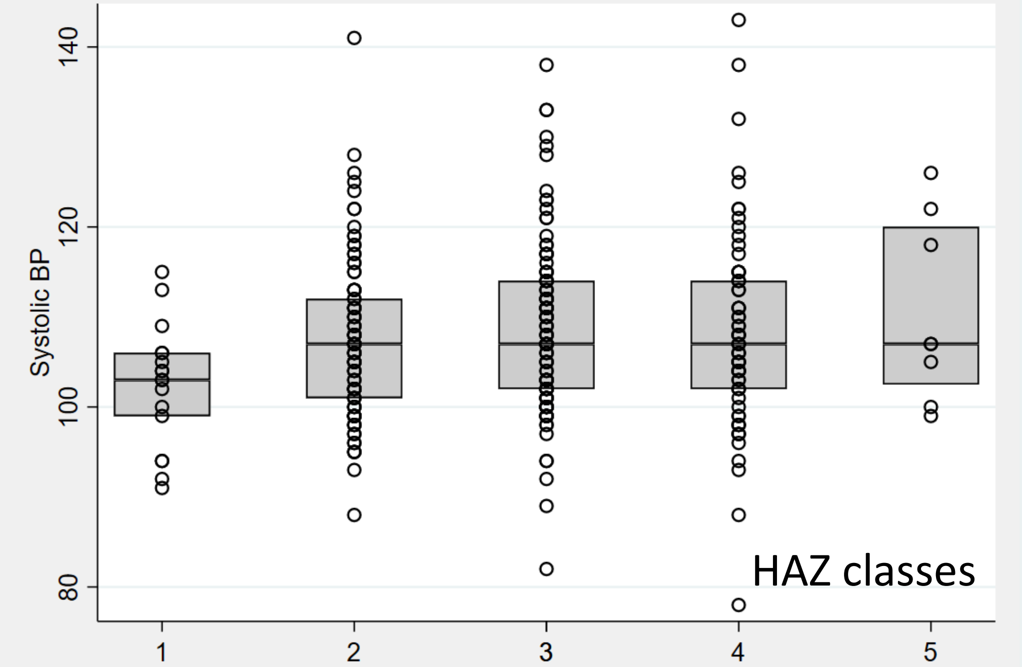
Systolic BP
140
120
100
80
1
2
3
4
5
HAZ classes

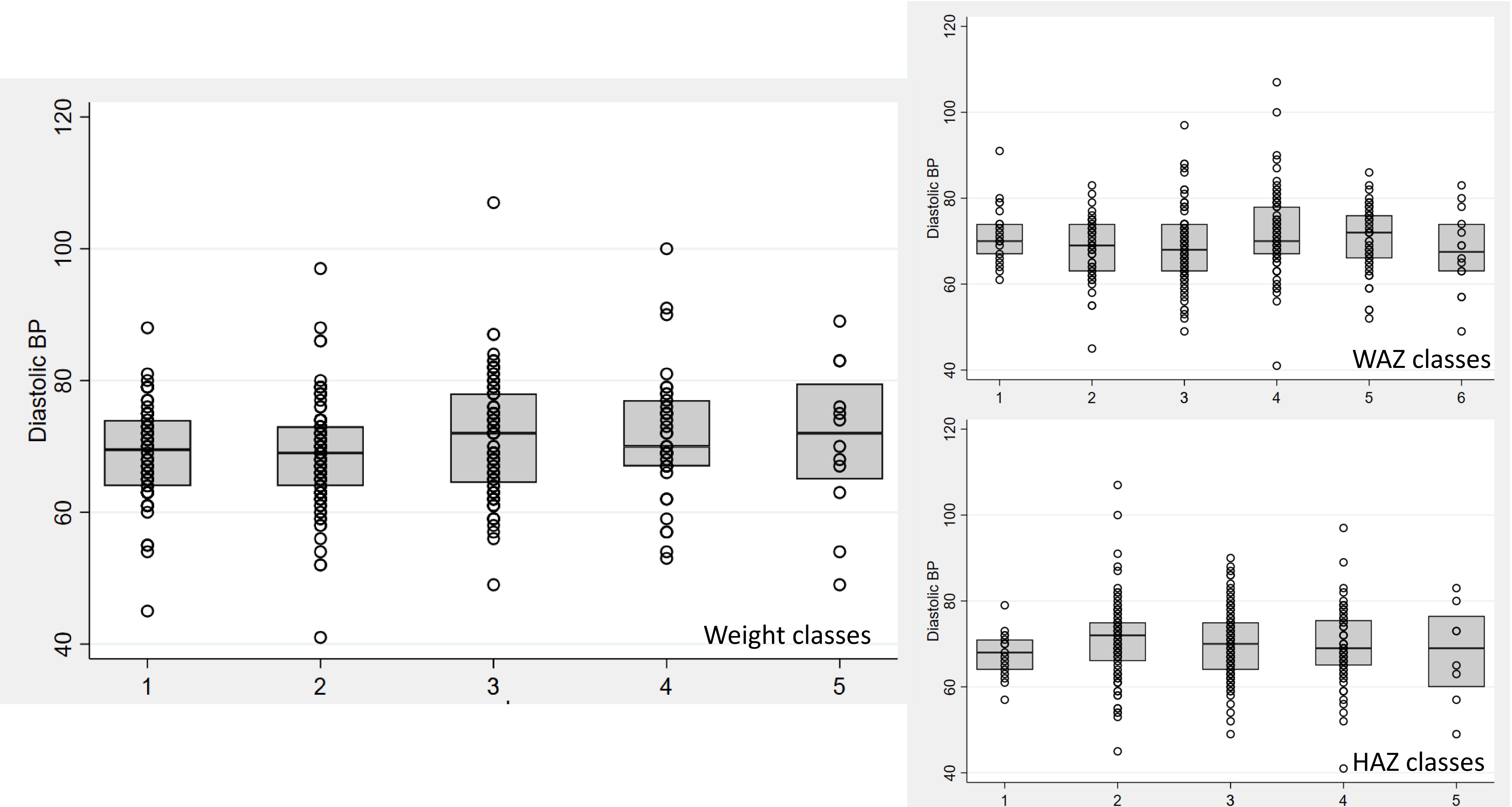

Diastolic BP
Weight classes
WAZ classes
HAZ classes

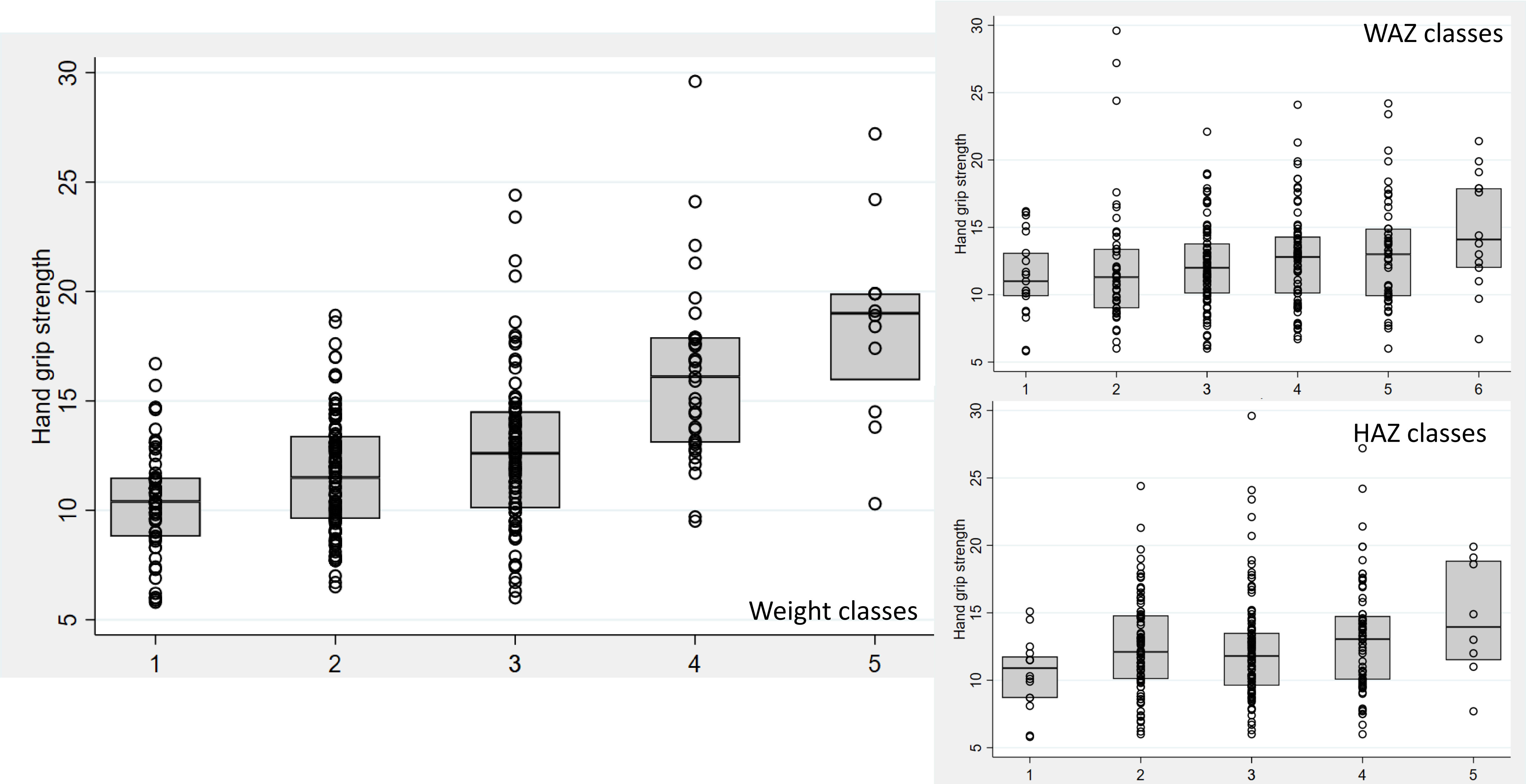

Hand grip strength
Weight classes
WAZ classes
HAZ classes

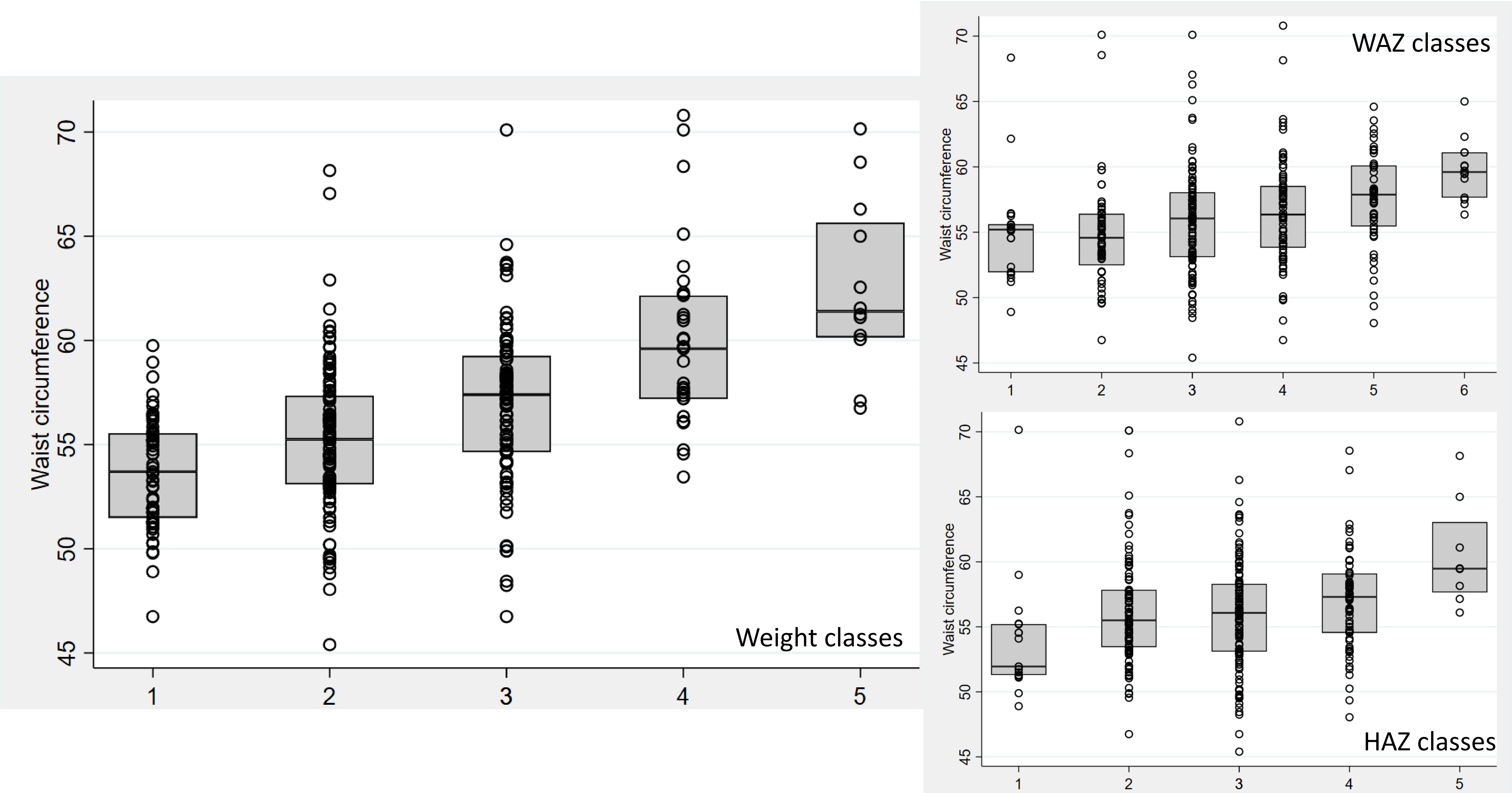

Waist circumference
70
65
60
55
50
45
1
2
3
4
5
Weight classes
Waist circumference
70
65
60
55
50
45
1
2
3
4
5
6
WAZ classes
Waist circumference
70
65
60
55
50
45
1
2
3
4
5
HAZ classes

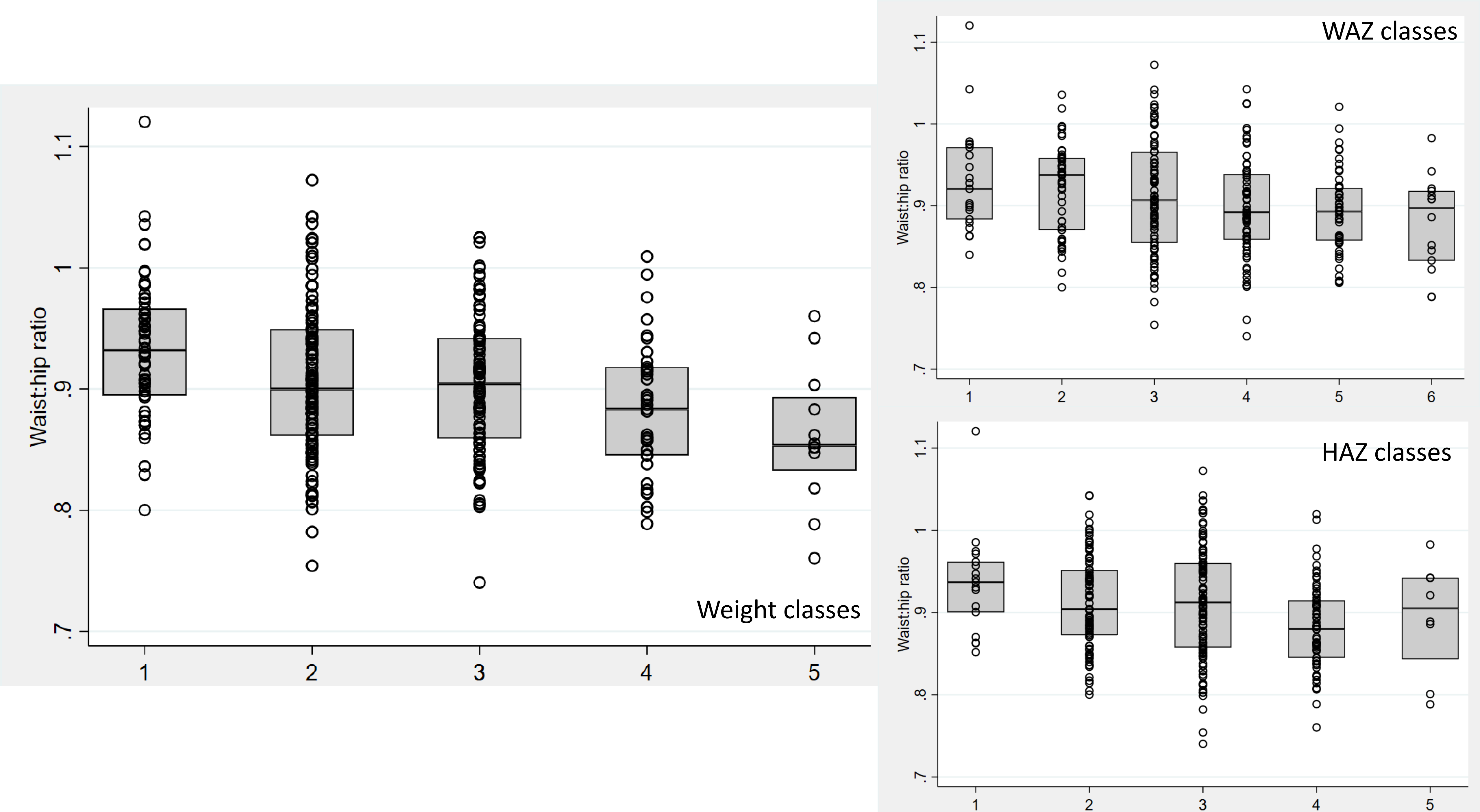
Waist:hip ratio
Weight classes
WAZ classes
HAZ classes
.7
.8
.9
1
1.1
1
2
3
4
5
6

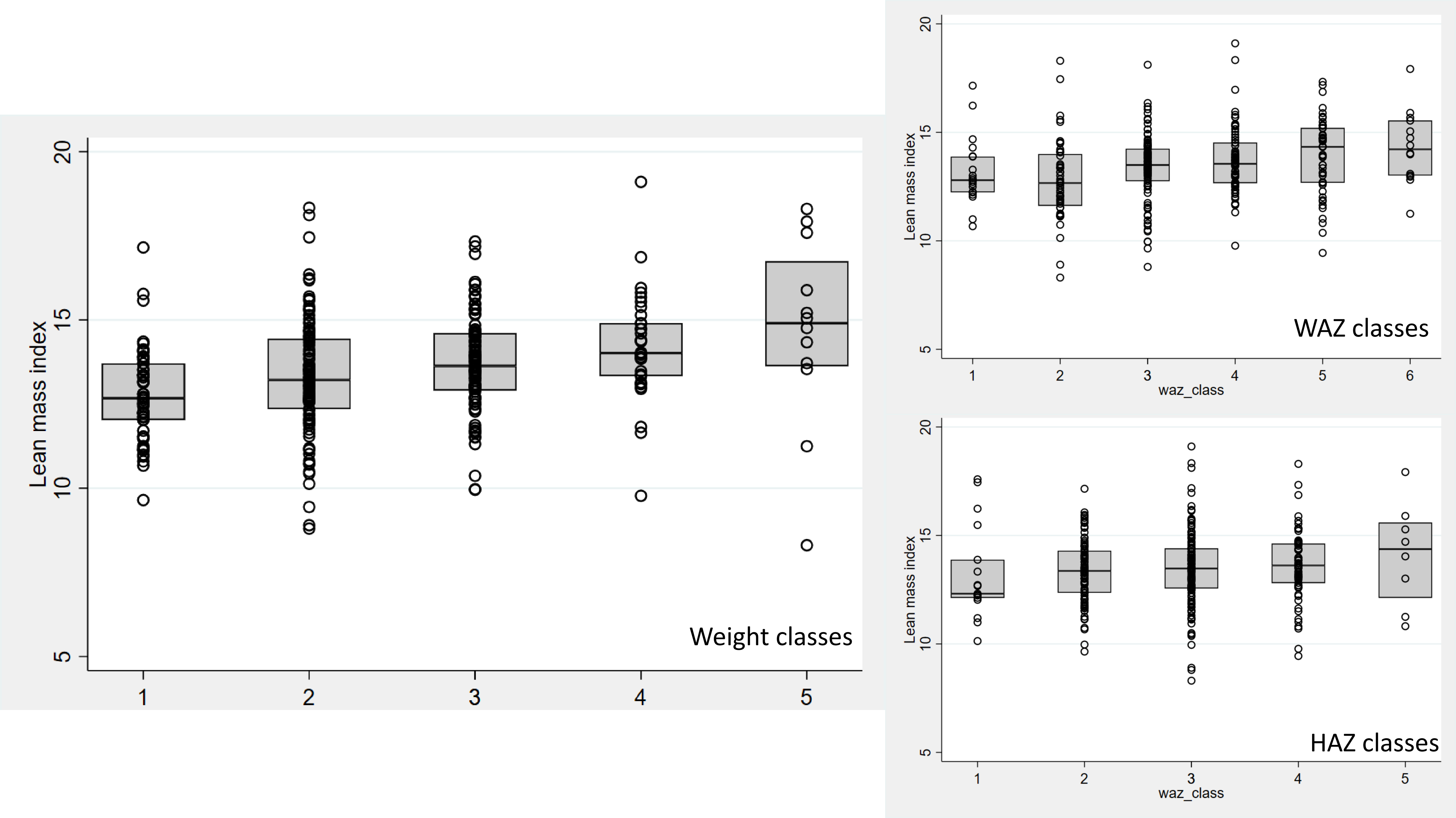
Lean mass index
20
15
10
5
1
2
3
4
5
Weight classes
Lean mass index
20
15
10
5
1
2
3
4
5
6
waz_class
WAZ classes
Lean mass index
20
15
10
5
1
2
3
4
5
waz_class
HAZ classes

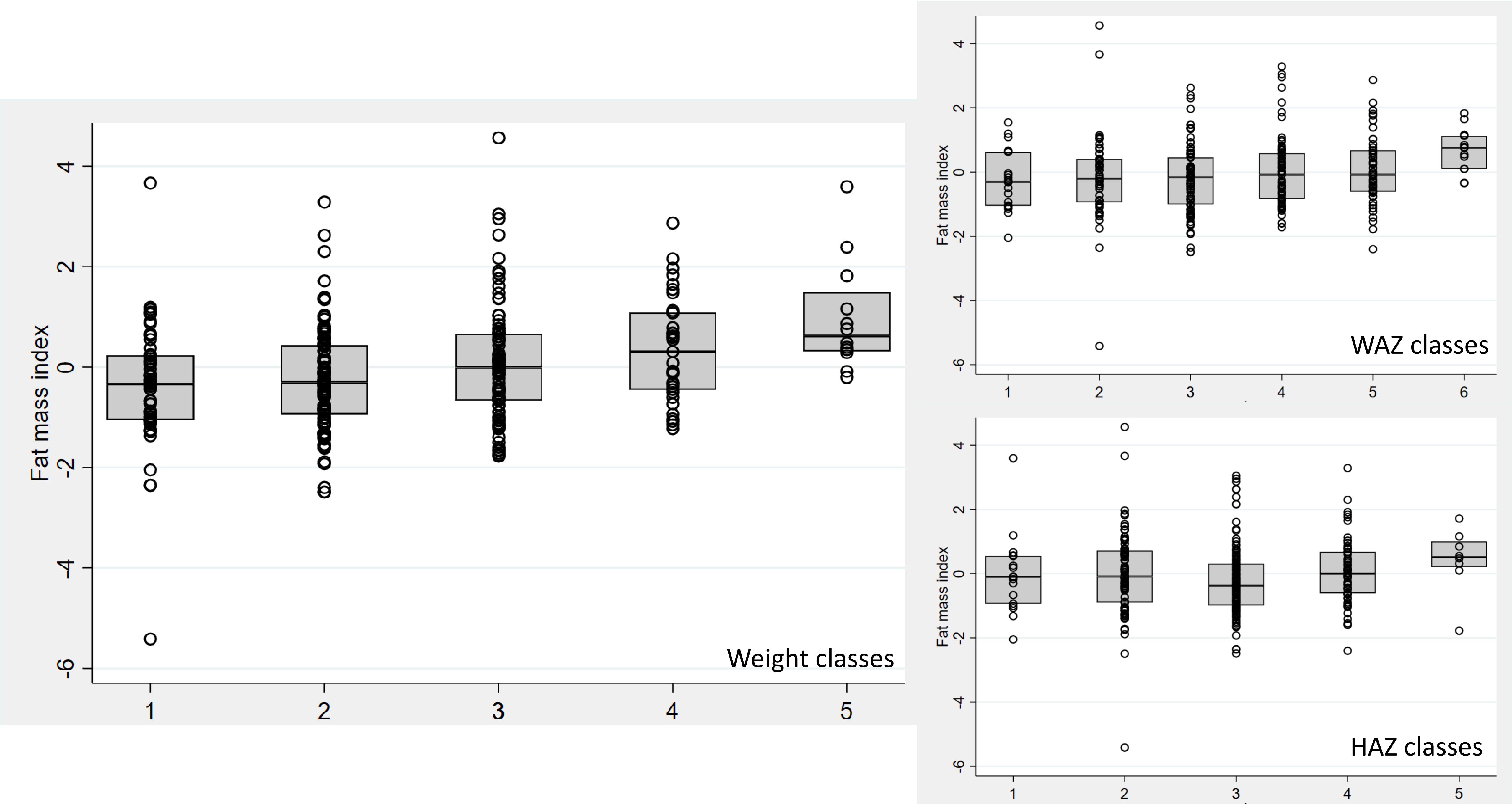

Fat mass index
4
2
0
-2
-4
-6
1
2
3
4
5
Weight classes
Fat mass index
4
2
0
-2
-4
-6
1
2
3
4
5
6
WAZ classes
Fat mass index
4
2
0
-2
-4
-6
1
2
3
4
5
HAZ classes

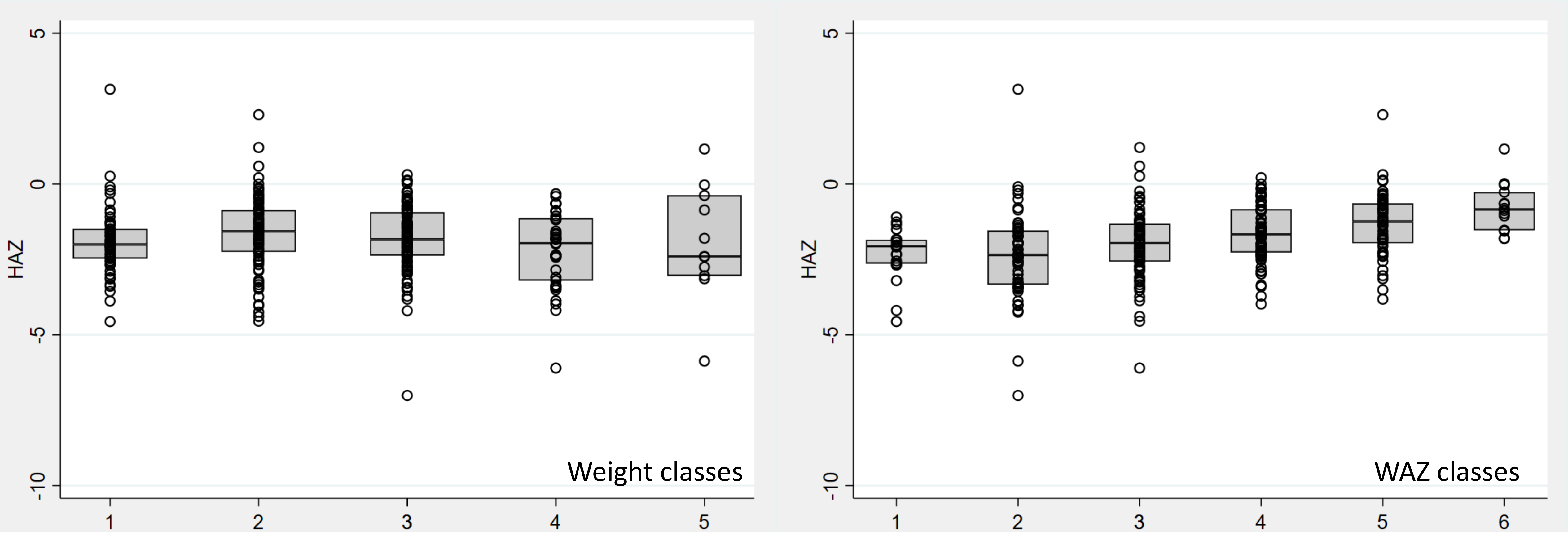

HAZ
5
0
-5
-10
1
2
3
4
5
Weight classes
HAZ
5
0
-5
-10
1
2
3
4
5
6
WAZ classes

Annex Table 5: Association between Admission anthropometry and NCD outcomes

NCD indicator	PMGr definition	Unadjusted difference	P-value	95% CI	Adjusted difference	P-value	95% CI
**Systolic BP**	Admission WAZ	**0.82**	**0.016**	**(0.15, 1.50)**	0.01	0.986	(-0.73, 0.74)
	Admission weight (kg)	**0.88**	**<0.001**	**(0.50, 1.27)**	-0.14	0.624	(-0.72, 0.43)
	Admission HAZ	0.68	0.061	(-0.03, 1.40)	-0.19	0.657	(-1.02, 0.64)
**Diastolic BP**	Admission WAZ	0.52	0.114	(-0.13, 1.17)	0.55	0.140	(-0.18, 1.28)
	Admission weight (kg)	0.25	0.159	(-0.10, 0.61)	0.07	0.797	(-0.49, 0.65)
	Admission HAZ	-0.15	0.661	(-0.84, 0.54)	-0.12	0.775	(-0.95, 0.71)
**Hand grip**	Admission WAZ	**0.63**	**<0.001**	(0.37, 0.89)	**0.49**	**<0.000**	**(0.24, 0.74)**
	Admission weight (kg)	**0.71**	**<0.001**	**(0.57, 0.85)**	**0.31**	**0.005**	**(0.09, 0.53)**
	Admission HAZ	**0.42**	**0.004**	**(0.14, 0.70)**	**0.53**	**<0.001**	**(0.28, 0.79)**
**Waist circumference**	Admission WAZ	**0.89**	**<0.001**	**(0.62, 1.15)**	0.87	**<0.001**	(0.60, 1.13)
	Admission weight (kg)	**0.82**	**<0.001**	**(0.68, 0.97)**	**0.52**	**<0.001**	**(0.30, 0.75)**
	Admission HAZ	**0.68**	**<0.001**	**(0.39, 0.98)**	0.85	**<0.001**	**(0.57, 1.12)**

NCD indicator	PMGr definition	Unadjusted difference	P-value	95% CI	Adjusted difference	P-value	95% CI
**Waist:Hip ratio**	Admission WAZ	**-0.01**	**<0.001**	**(-0.01, -0.00)**	**-0.01**	**0.004**	**(-0.01, -0.00)**
	Admission weight (kg)	-0.01	**<0.001**	**(-0.01, -0.00)**	**-0.01**	**0.008**	**(-0.01, -0.00)**
	Admission HAZ	**-0.01**	**0.001**	**(-0.01, -0.00)**	**-0.08**	**0.001**	(-0.01, -0.00)
**Lean mass index (BIA)**	Admission WAZ	**0.25**	**<0.001**	**(0.12, 0.37)**	**0.24**	**<0.001**	**(0.11, 0.38)**
	Admission weight (kg)	**0.18**	**<0.001**	**(0.11, 0.25)**	**0.18**	**0.001**	**(0.07, 0.29)**
	Admission HAZ	0.09	0.206	(-0.05, 0.22)	0.09	0.210	(-0.05, 0.23)
**Fat mass index (BIA)**	Admission WAZ	**0.13**	**0.003**	**(0.05, 0.21)**	**0.14**	**0.002**	**(0.06, 0.24)**
	Admission weight (kg)	**0.12**	**<0.001**	**(0.08, 0.17)**	**0.10**	**0.005**	**(0.03, 0.18)**
	Admission HAZ	0.08	0.097	(-0.01, 0.17)	**0.11**	**0.024**	**(0.01, 0.19)**
**HAZ**	Admission WAZ	**0.22**	**<0.001**	**(0.13, 0.29)**	**0.25**	**<0.001**	**(0.16, 0.33)**
	Admission weight (kg)	-0.03	0.313	(-0.08, 0.03)	**0.15**	**<0.001**	**(0.08, 0.22**
	Admission HAZ	**0.40**	**<0.001**	**(0.32, 0.48)**	**0.39**	**<0.001**	**(0.31, 0.47)**

Annex Figure 6

Growth patterns for WAZ, weight and HAZ described based on LCA classes which have been standardised by admission anthropometry

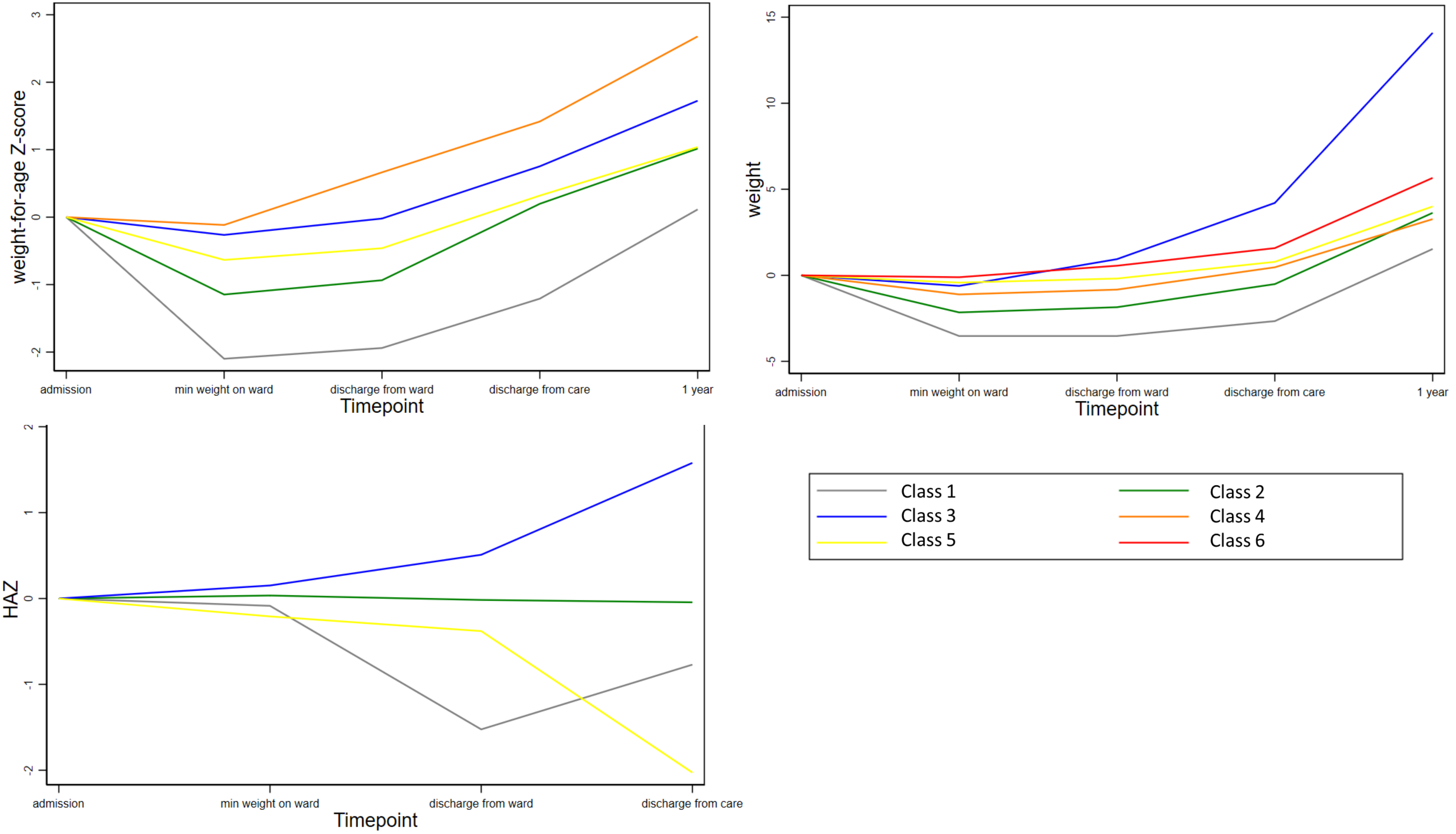
weight-for-age Z-score
3
2
1
0
-1
-2
admission
min weight on ward
discharge from ward
discharge from care
1 year
Timepoint
weight
15
10
5
0
-5
admission
min weight on ward
discharge from ward
discharge from care
1 year
Timepoint
HAZ
2
1
0
-1
-2
admission
min weight on ward
discharge from ward
discharge from care
Timepoint
Class 1
Class 2
Class 3
Class 4
Class 5
Class 6

Annex Figures 7a-5g

Boxplots showing associations between LCA classes for weight, WAZ and HAZ (standardised for admission anthropometry), and NCD risk outcomes

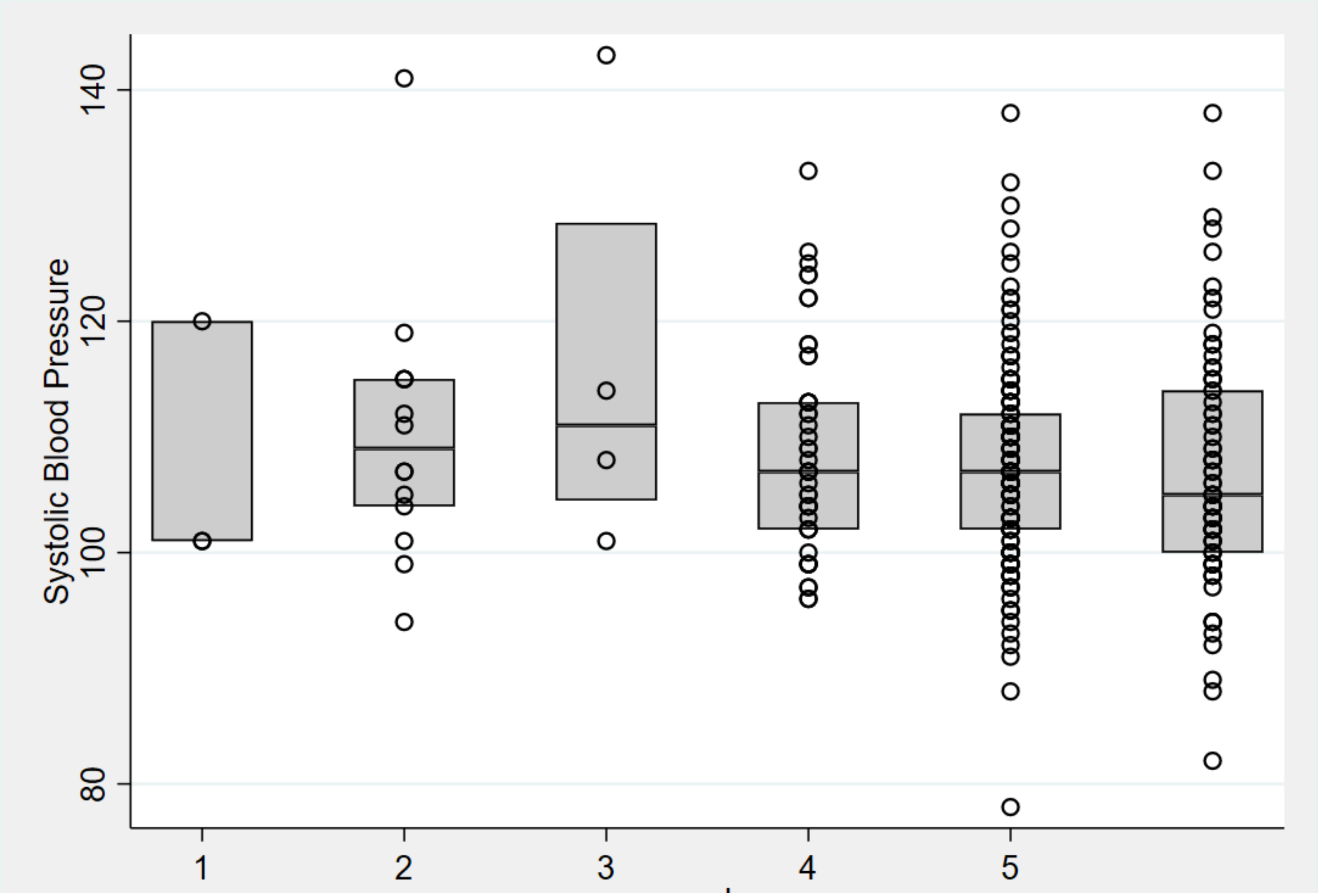
Systolic Blood Pressure
140
120
100
80
1
2
3
4
5

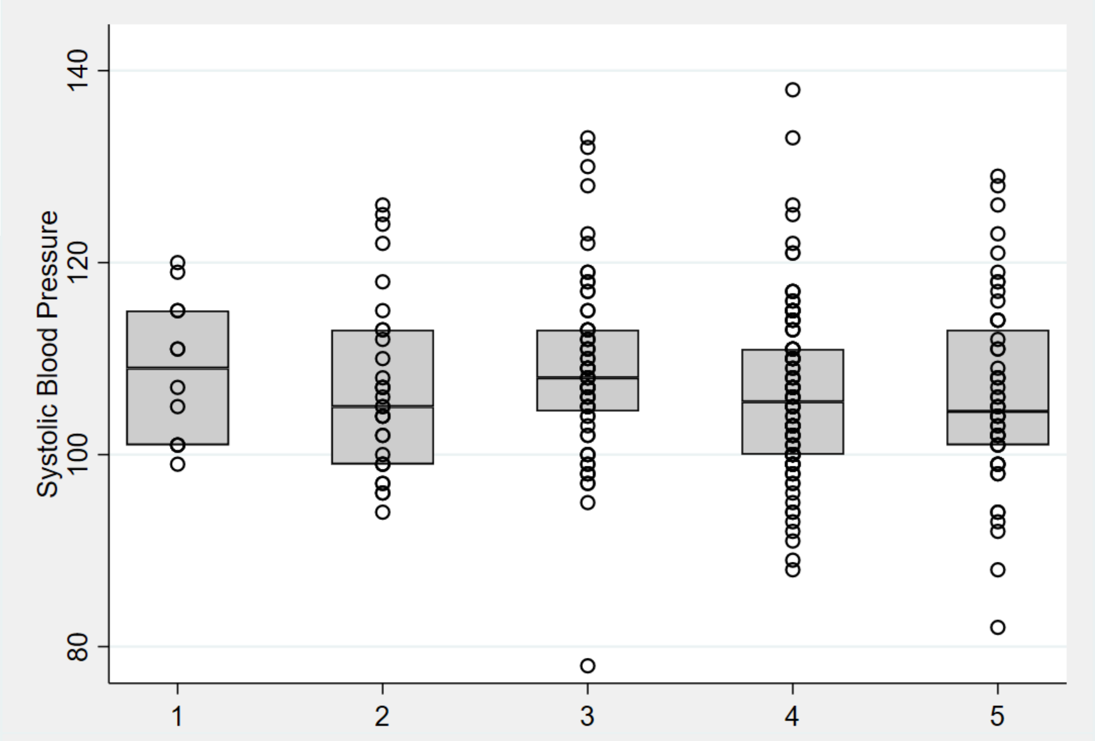
Systolic Blood Pressure
140
120
100
80
1
2
3
4
5

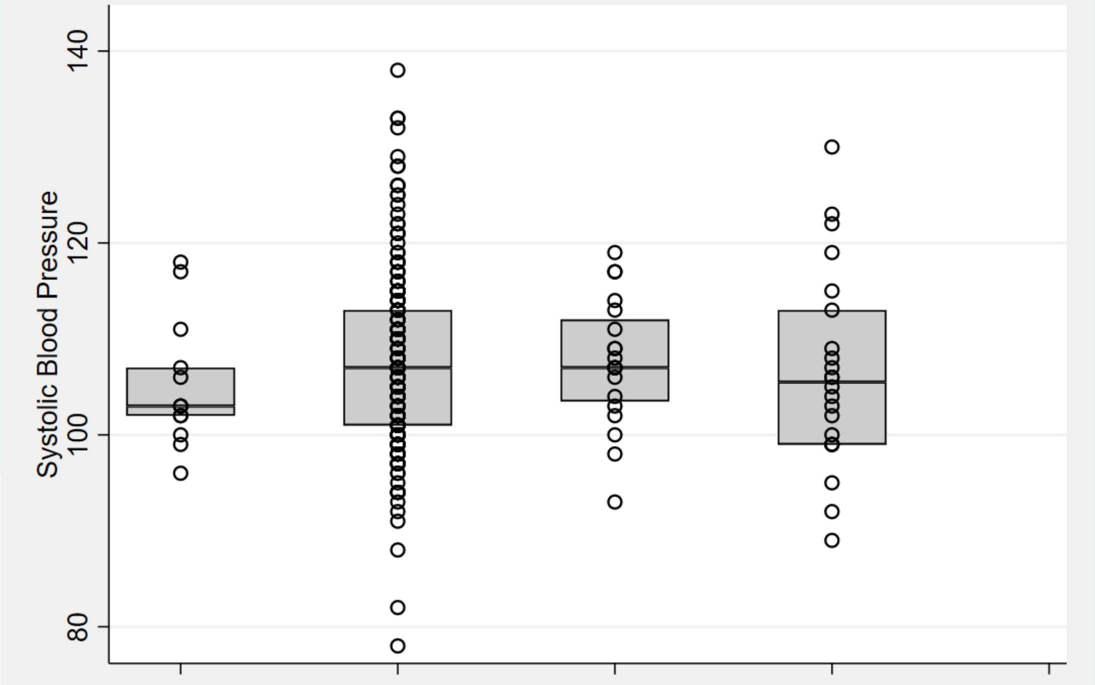
Systolic Blood Pressure
140
120
100
80

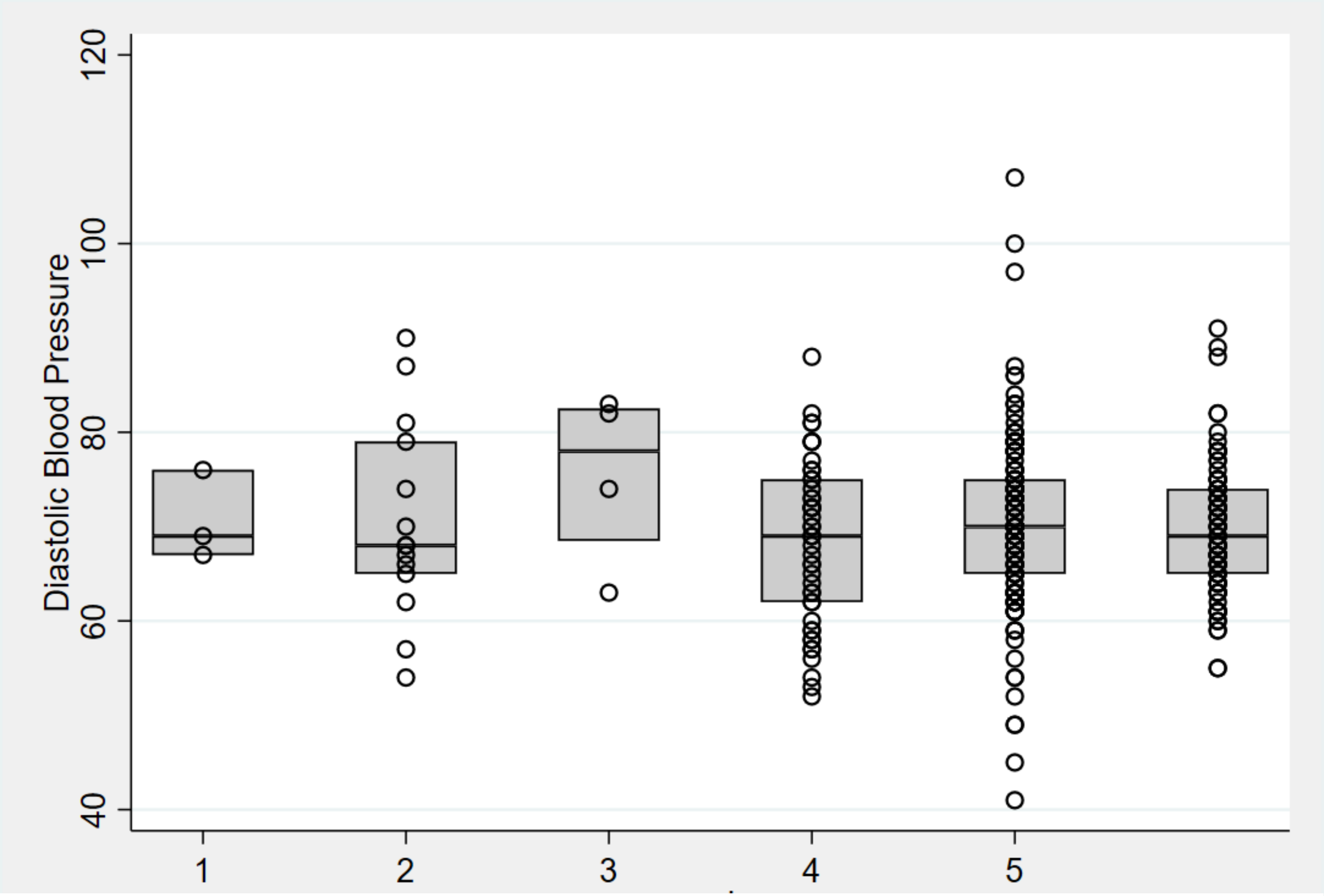
Diastolic Blood Pressure
40
60
80
100
120
1
2
3
4
5

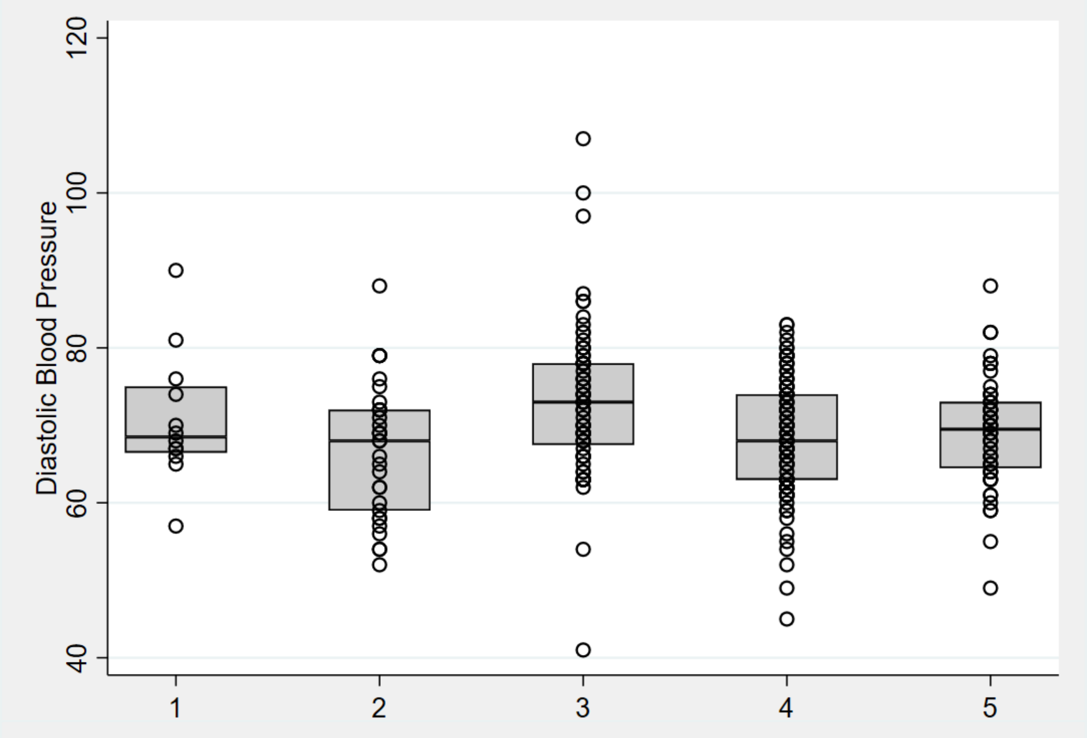
Diastolic Blood Pressure
40
60
80
100
120
1
2
3
4
5

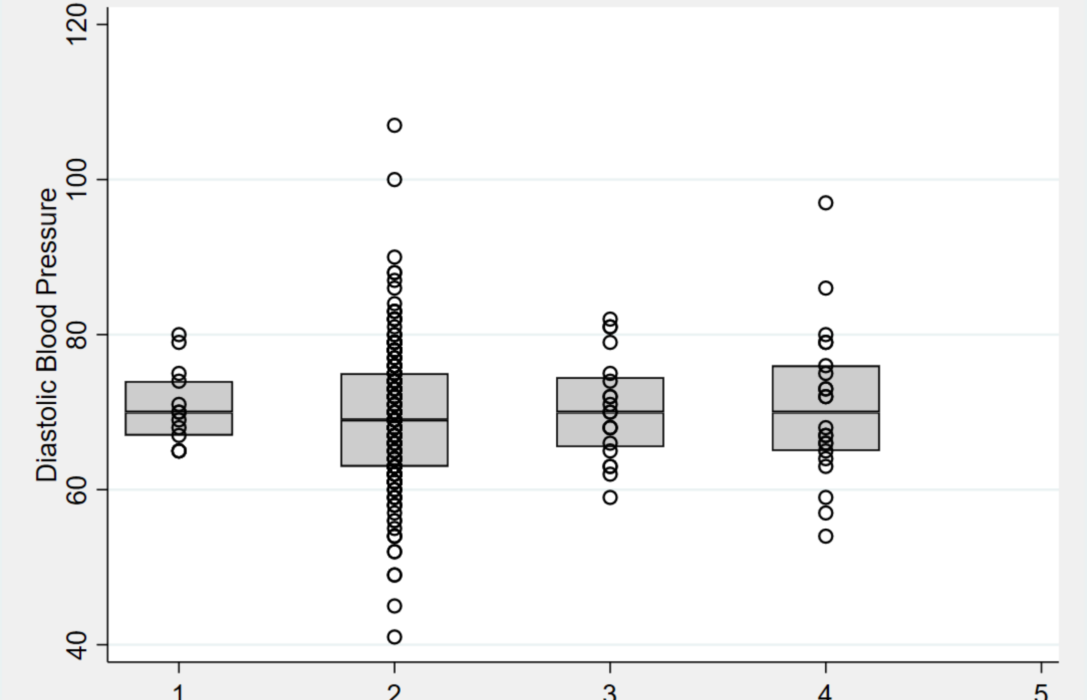
Diastolic Blood Pressure
40
60
80
100
120
1
2
3
4
5

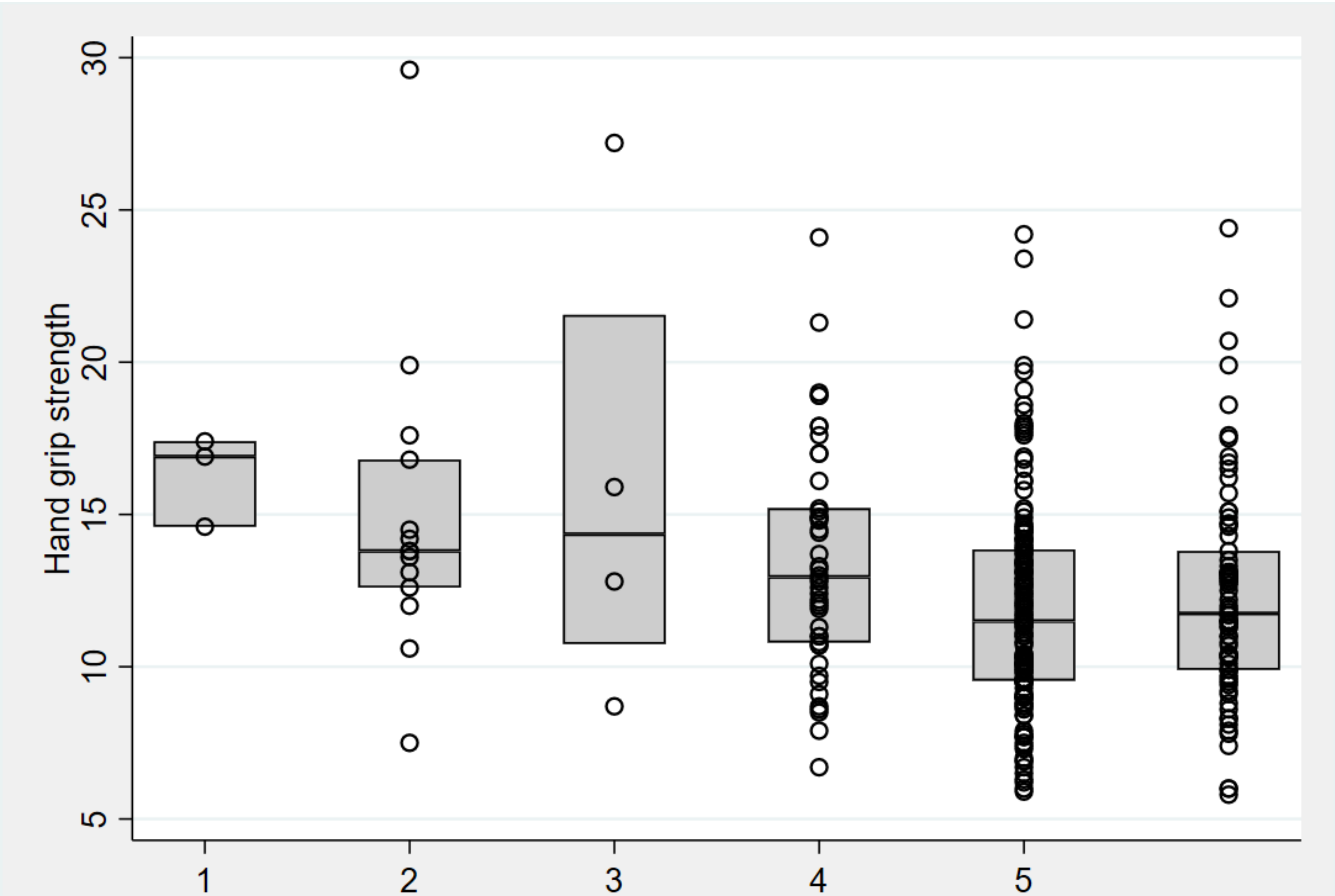
Hand grip strength
30
25
20
15
10
5
1
2
3
4
5

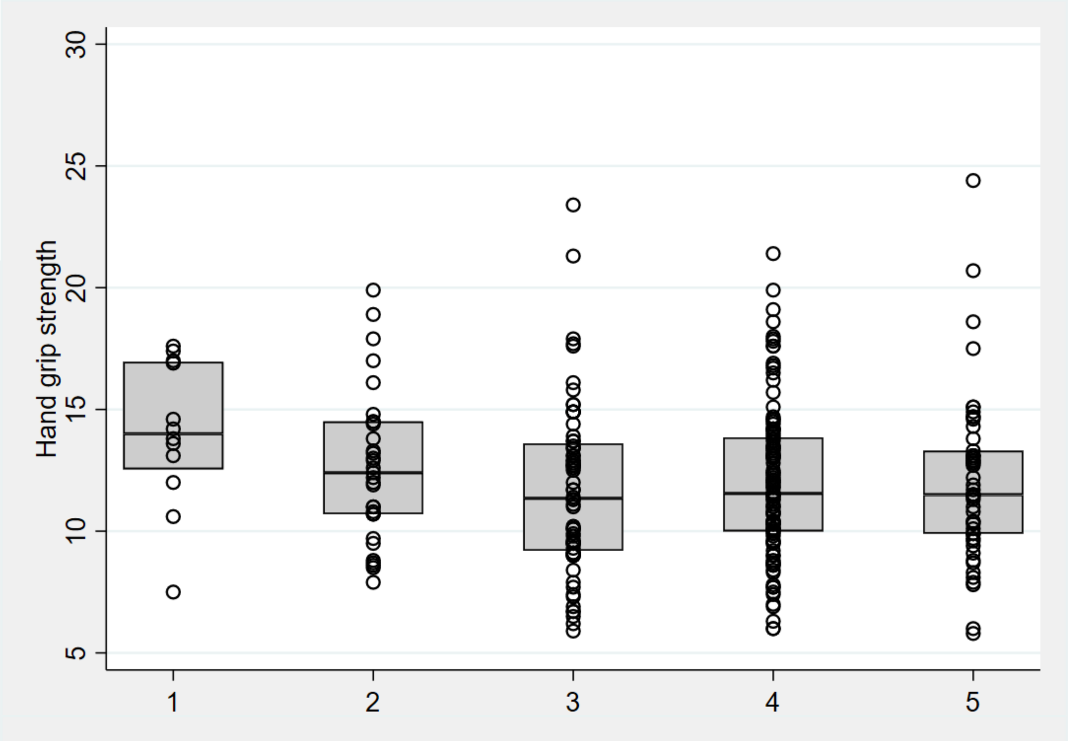
Hand grip strength
30
25
20
15
10
5
1
2
3
4
5

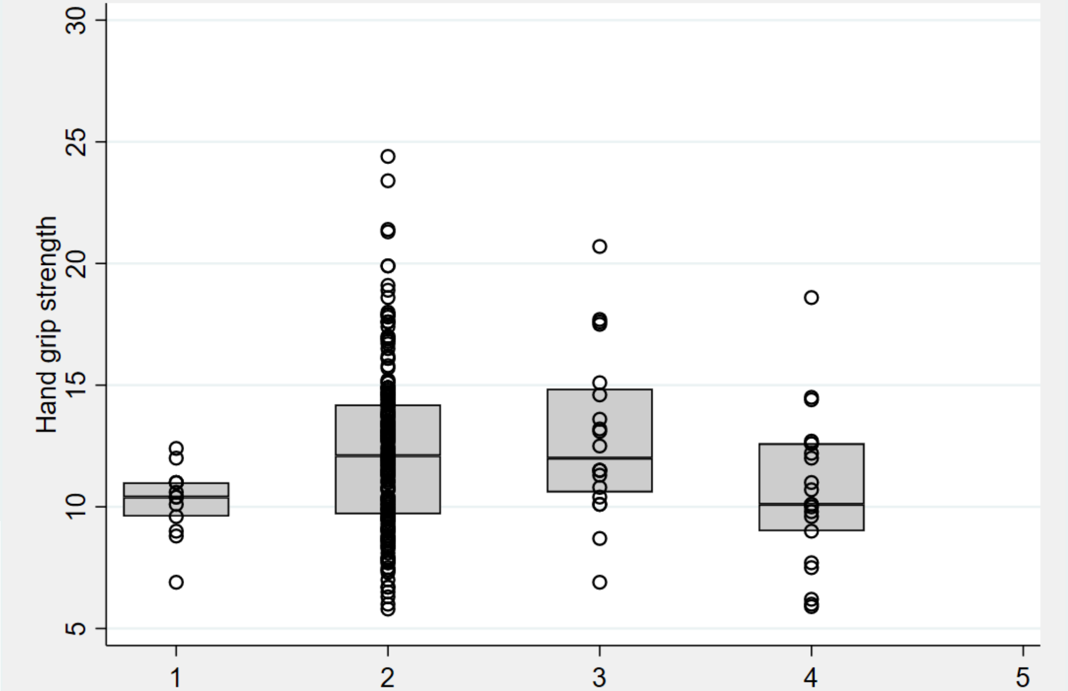
Hand grip strength
30
25
20
15
10
5
1
2
3
4
5

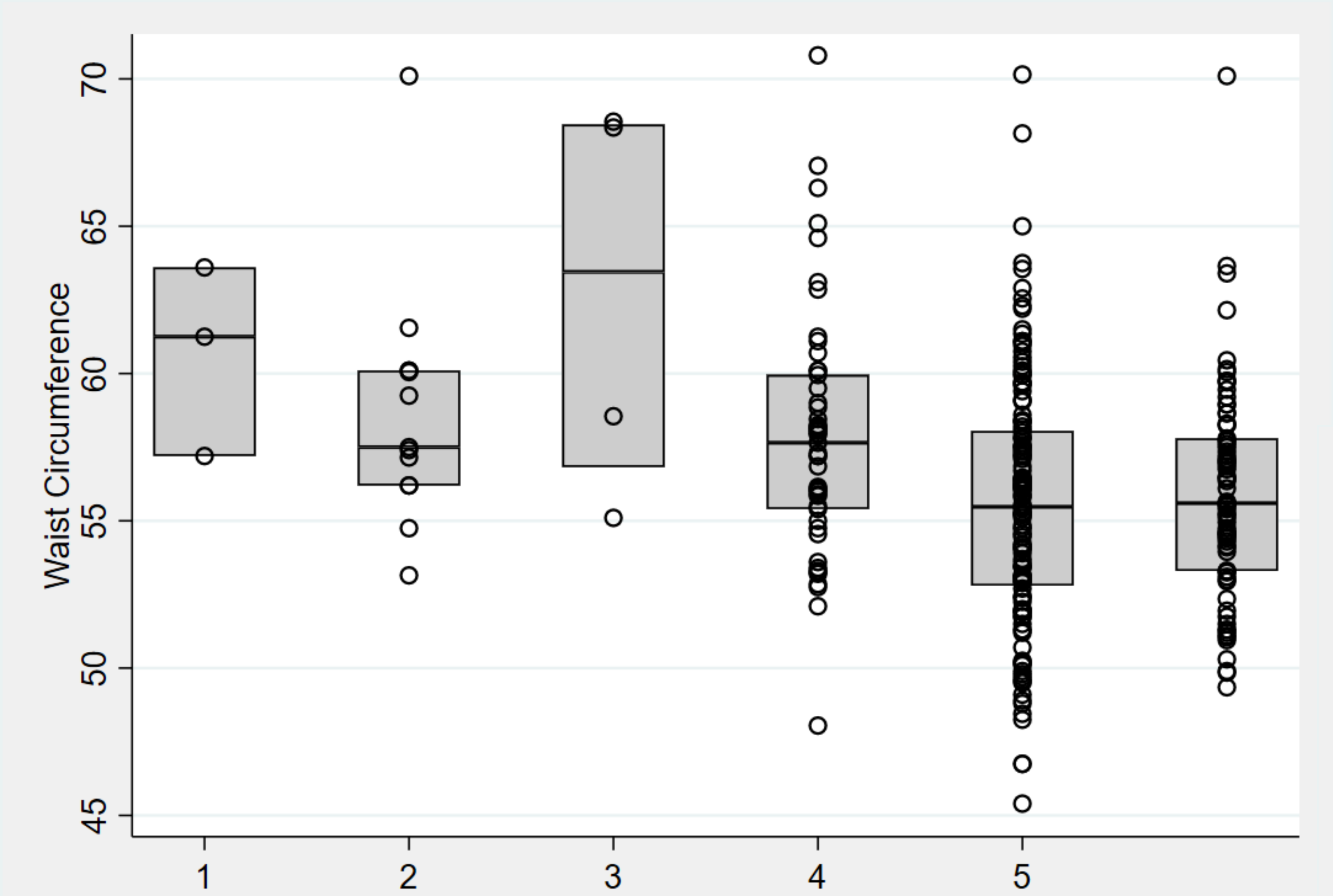
Waist Circumference
45
50
55
60
65
70
1
2
3
4
5

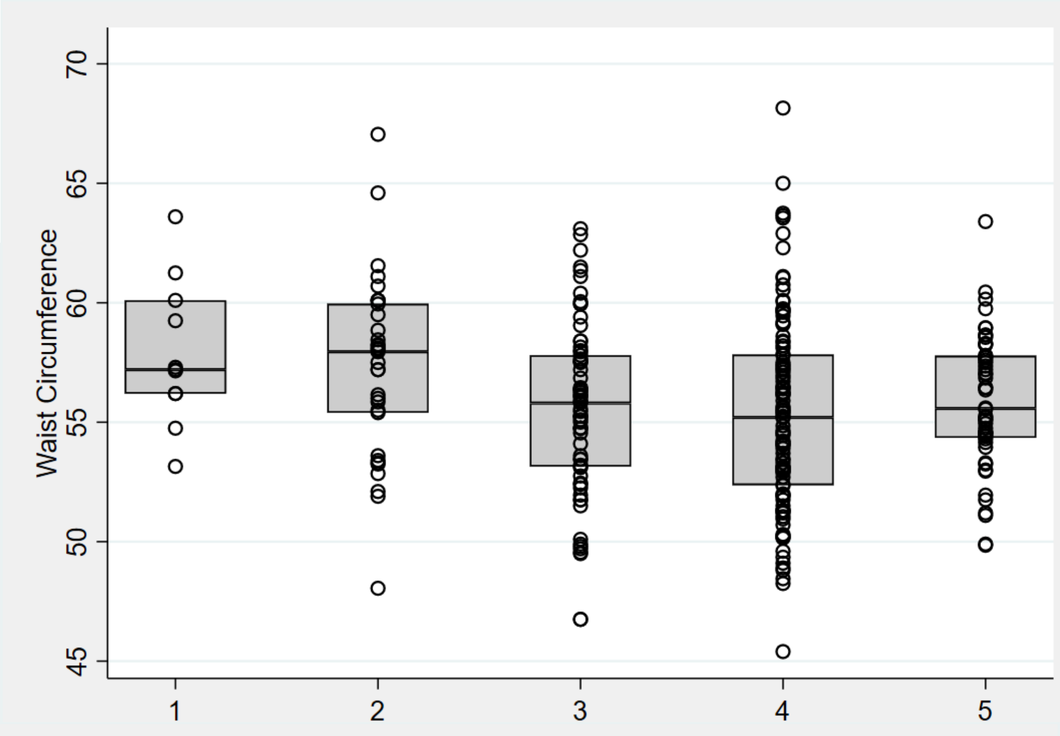
Waist Circumference
45
50
55
60
65
70
1
2
3
4
5

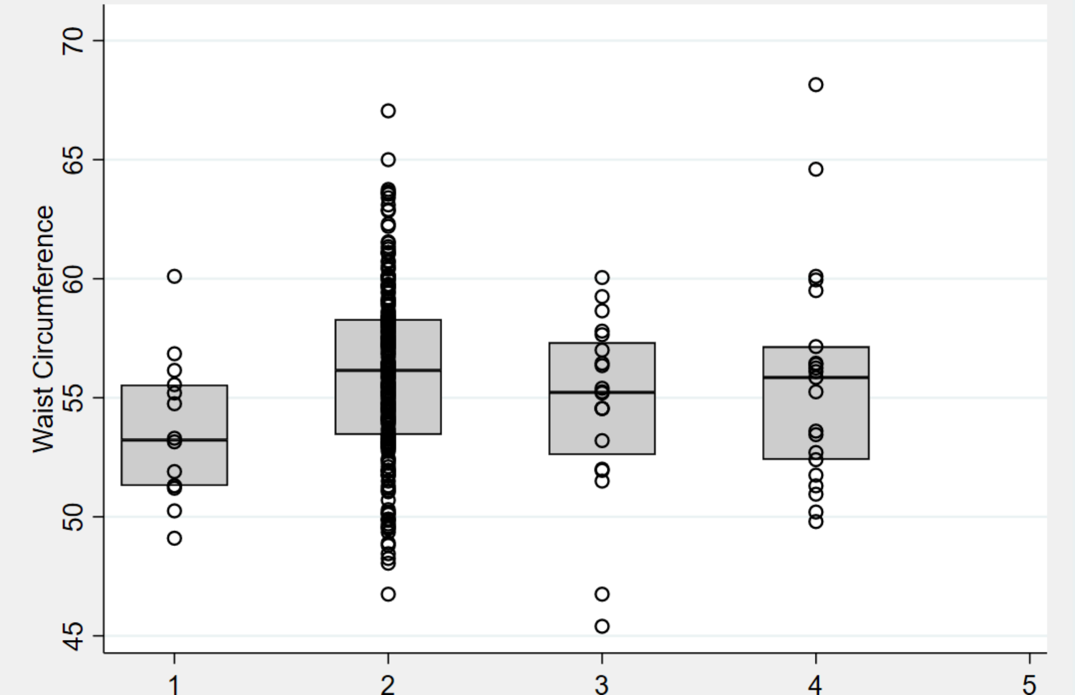
Waist Circumference
45
50
55
60
65
70
1
2
3
4
5

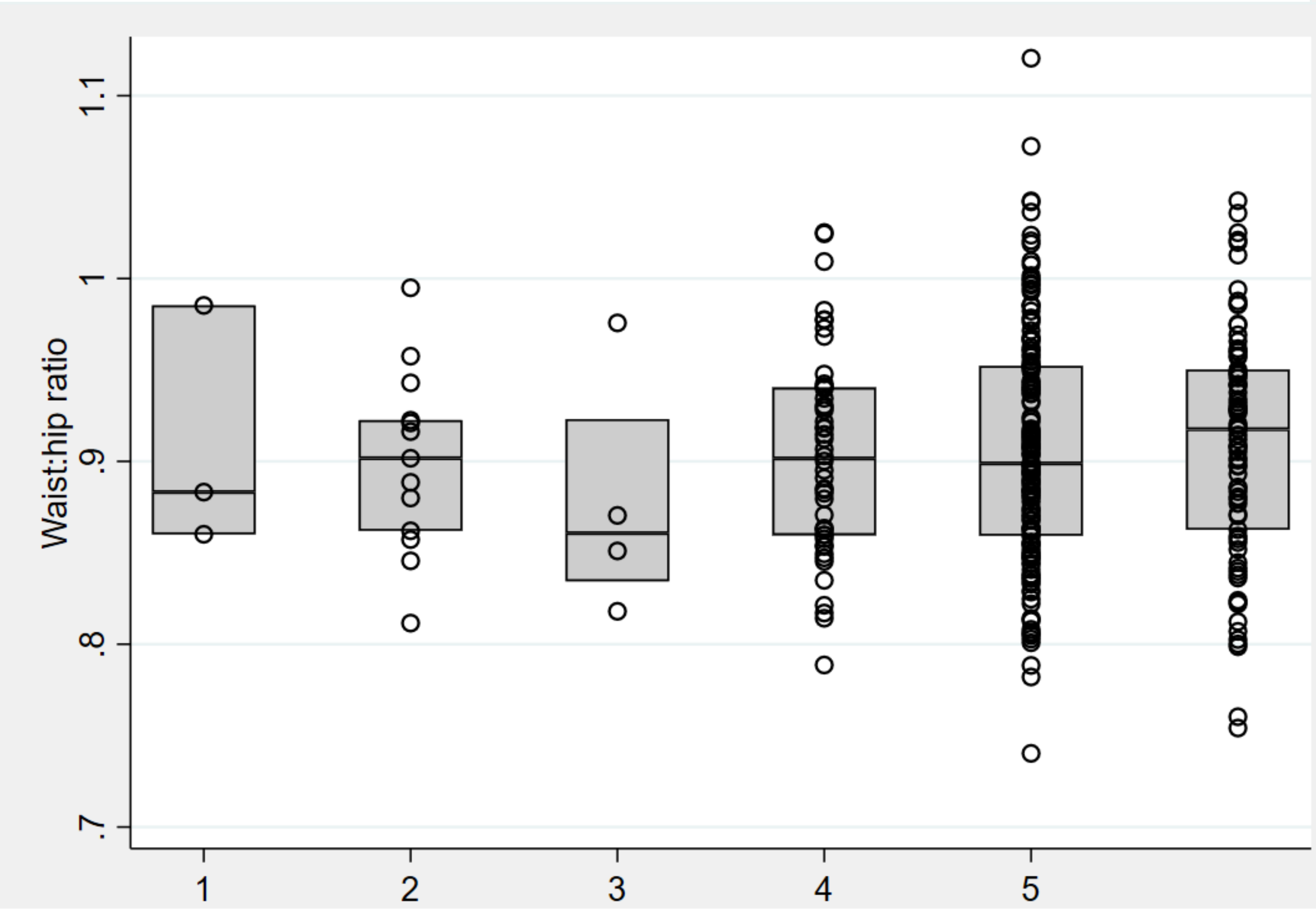
Waist:hip ratio
.7
.8
.9
1
1.1
1
2
3
4
5

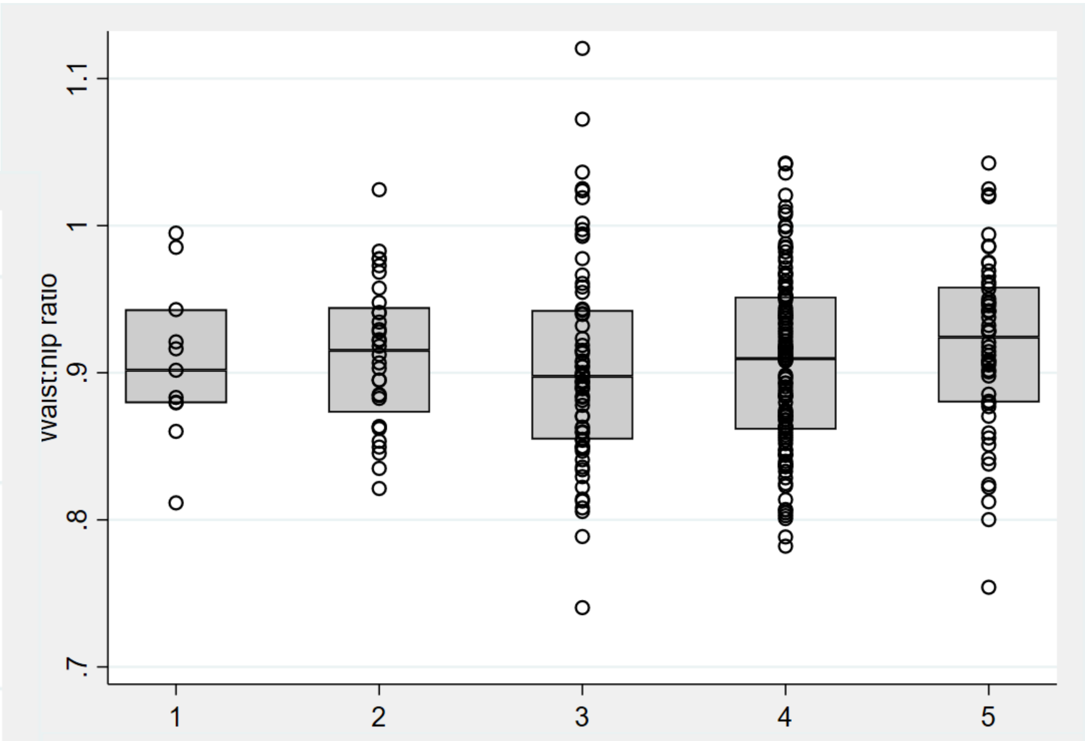
waist:hip ratio
.7
.8
.9
1
1.1
1
2
3
4
5

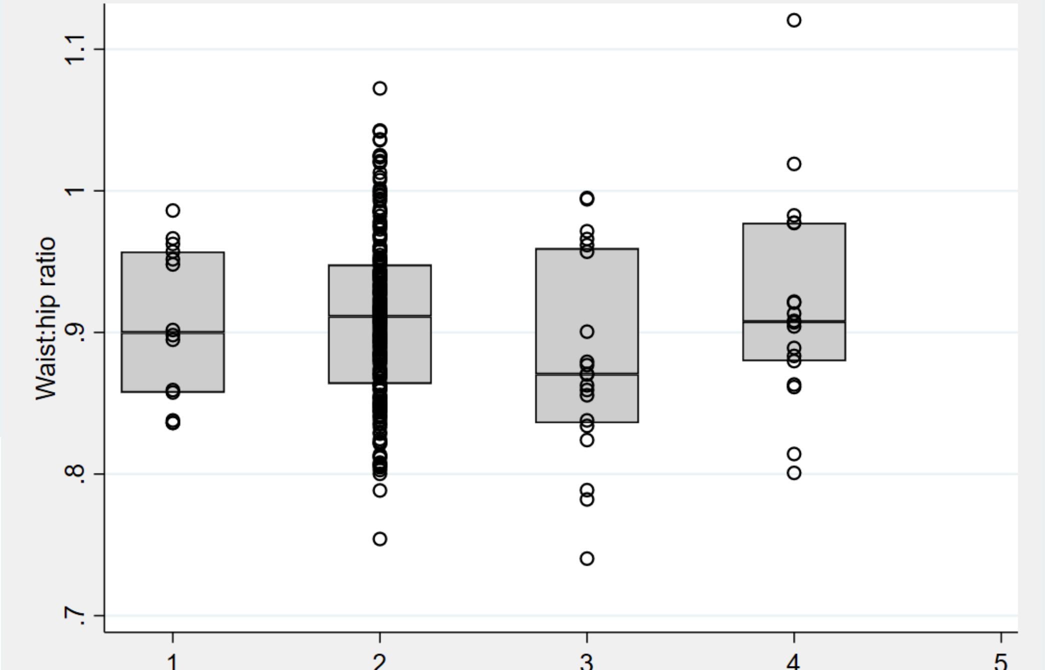
Waist:hip ratio
.7
.8
.9
1
1.1
1
2
3
4
5

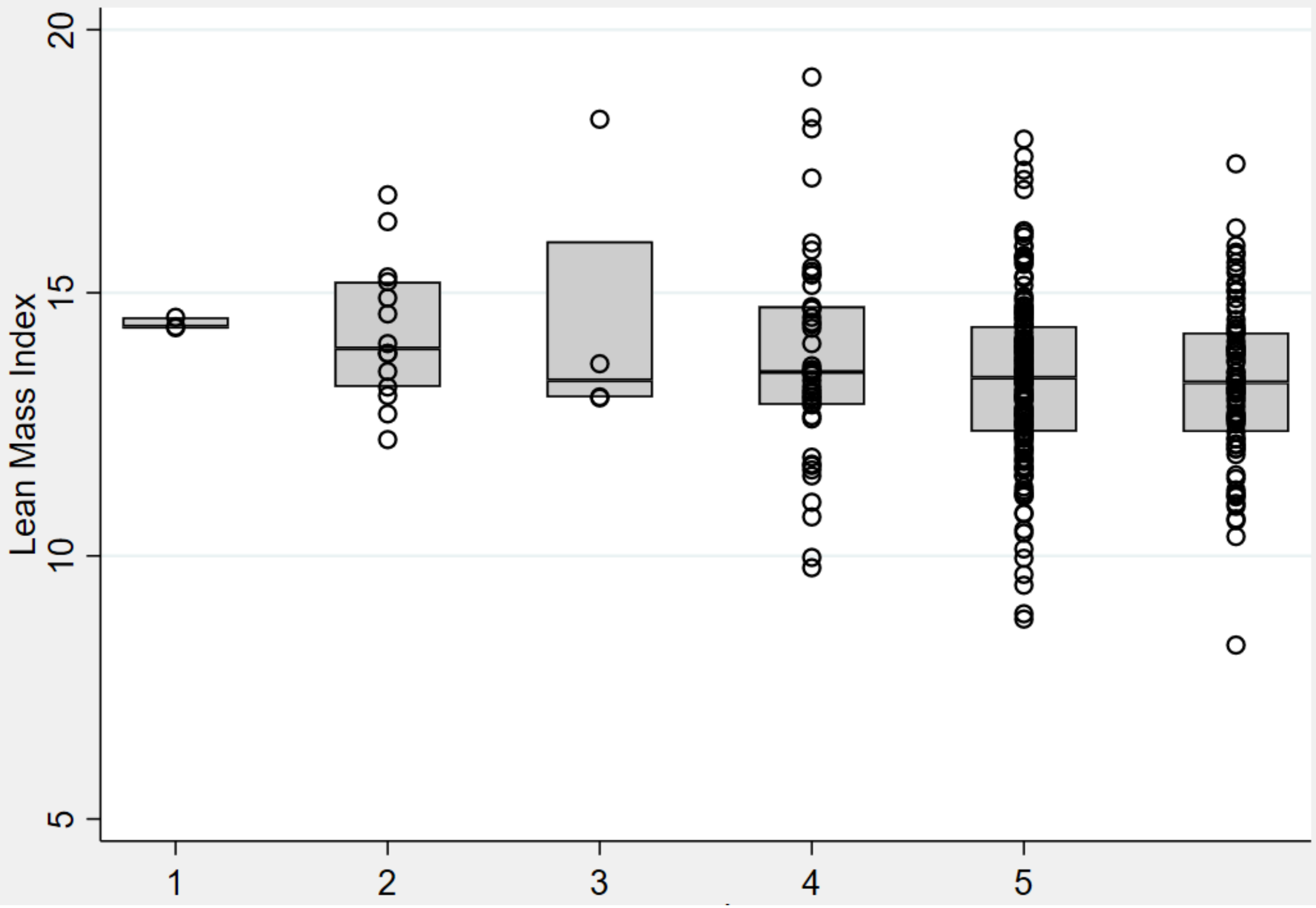
Lean Mass Index
20
15
10
5
1
2
3
4
5

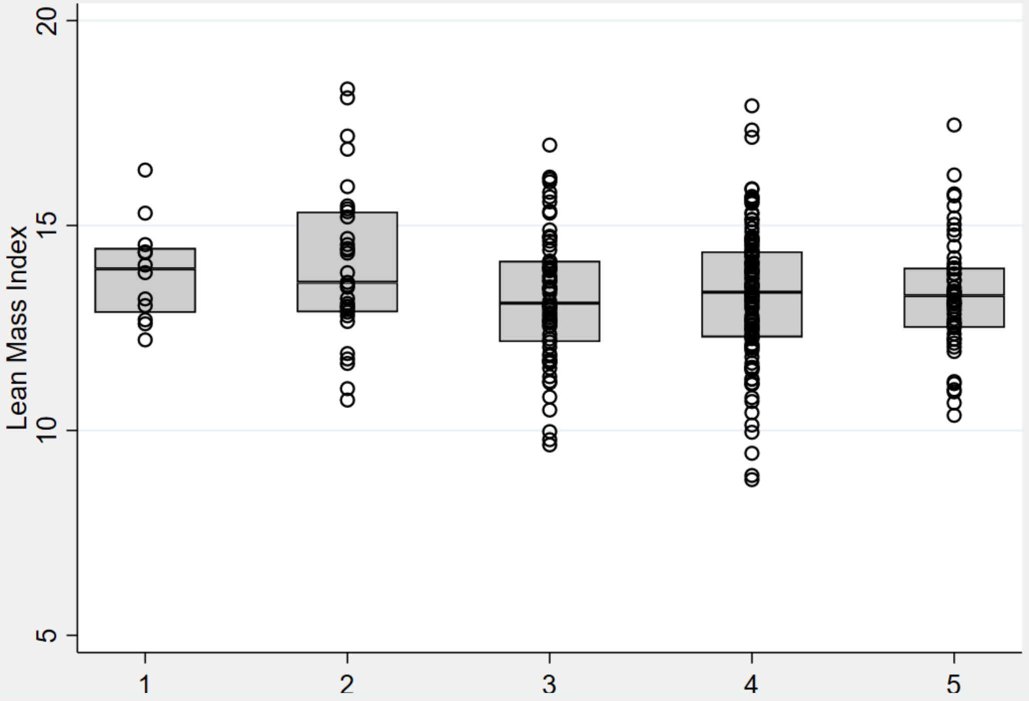
Lean Mass Index
20
15
10
5
1
2
3
4
5

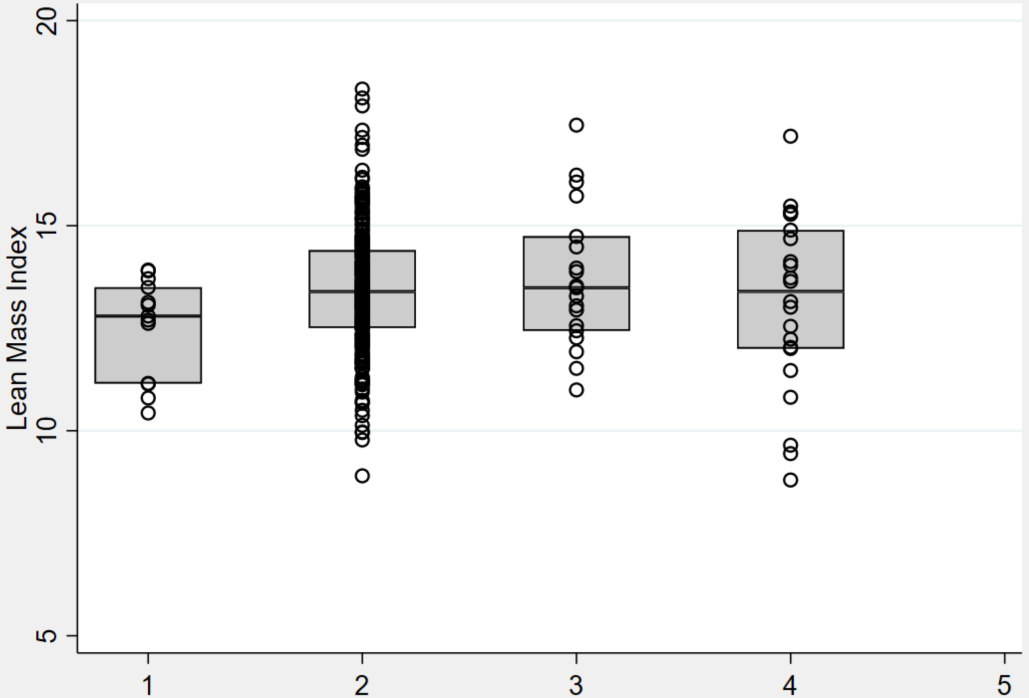
Lean Mass Index
20
15
10
5
1
2
3
4
5

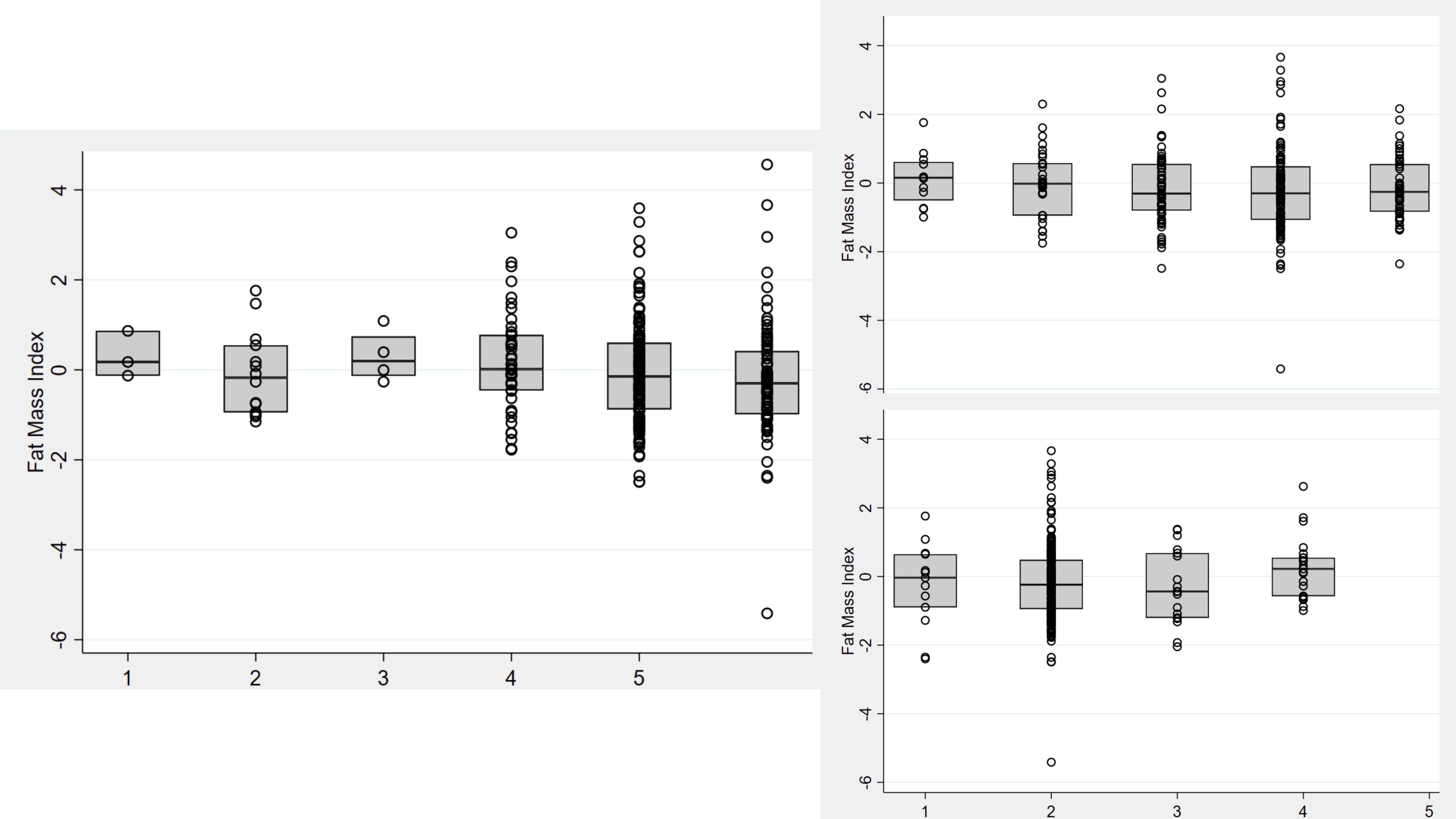

Fat Mass Index
4
2
0
-2
-4
-6
1
2
3
4
5
Fat Mass Index
4
2
0
-2
-4
-6
Fat Mass Index
4
2
0
-2
-4
-6
1
2
3
4
5
